# Gut-Brain Nexus: Mapping Multi-Modal Links to Neurodegeneration at Biobank Scale

**DOI:** 10.1101/2024.09.12.24313490

**Published:** 2024-09-13

**Authors:** Mohammad Shafieinouri, Samantha Hong, Artur Schuh, Mary B. Makarious, Rodrigo Sandon, Paul Suhwan Lee, Emily Simmonds, Hirotaka Iwaki, Gracelyn Hill, Cornelis Blauwendraat, Valentina Escott-Price, Yue A. Qi, Alastair J. Noyce, Armando Reyes-Palomares, Hampton L. Leonard, Malu Tansey, Anant Dadu, Faraz Faghri, Andrew Singleton, Mike A. Nalls, Kristin S. Levine, Sara Bandres-Ciga

**Affiliations:** 1 - Center for Alzheimer’s and Related Dementias, National Institutes of Health, Bethesda, MD, USA 20892.; 2 - Departamento de Farmacologia, Universidade Federal do Rio Grande do Sul, Porto Alegre, Brazil; 3 - Serviço de Neurologia, Hospital de Clínicas de Porto Alegre, Porto Alegre, Brazil; 4 - DataTecnica LLC, Washington, DC, USA 20037.; 5 - UK Dementia Research Institute (UK DRI) at Cardiff University, Cardiff, UK; 6 - Laboratory of Neurogenetics, National Institute on Aging, Bethesda, MD, USA; 7 - Division of Psychological Medicine and Clinical Neurosciences, School of Medicine, Cardiff University, Cardiff, UK; 8 - Centre for Preventive Neurology, Wolfson Institute of Population Health, Queen Mary University of London, London, UK.; 9 - Department of Molecular Biology and Biochemistry, Faculty of Sciences, University of Málaga, Málaga, Spain; 10 - Department of Neuroscience, Center for Translational Research in Neurodegenerative Disease, University of Florida College of Medicine, Gainesville, FL, USA.; 11 - Department of Neurology, Norman Fixel Institute for Neurological Diseases, University of Florida Health, Gainesville, FL, USA.

**Keywords:** Alzheimer’s disease, Parkinson’s disease, endocrine, nutritional, metabolic, digestive system, polygenic risk score, interaction, risk, multi-modal classification, proteomics, genetics, prediction

## Abstract

Alzheimer’s disease (AD) and Parkinson’s disease (PD) are influenced by genetic and environmental factors. Using data from UK Biobank, SAIL Biobank, and FinnGen, we conducted an unbiased, population-scale study to: 1) Investigate how 155 endocrine, nutritional, metabolic, and digestive system disorders are associated with AD and PD risk prior to their diagnosis, considering known genetic influences; 2) Assess plasma biomarkers’ specificity for AD or PD in individuals with these conditions; 3) Develop a multi-modal classification model integrating genetics, proteomics, and clinical data relevant to conditions affecting the gut-brain axis. Our findings show that certain disorders elevate AD and PD risk before AD and PD diagnosis including: insulin and non-insulin dependent diabetes mellitus, noninfective gastro-enteritis and colitis, functional intestinal disorders, and bacterial intestinal infections, among others. Polygenic risk scores revealed lower genetic predisposition to AD and PD in individuals with co-occurring disorders in the study categories, underscoring the importance of regulating the gut-brain axis to potentially prevent or delay the onset of neurodegenerative diseases. The proteomic profile of AD/PD cases was influenced by comorbid endocrine, nutritional, metabolic, and digestive systems conditions. Importantly, we developed multi-modal prediction models integrating clinical, genetic, proteomic and demographic data, the combination of which performs better than any single paradigm approach in disease classification. This work aims to illuminate the intricate interplay between various physiological factors involved in the gut-brain axis and the development of AD and PD, providing a multifactorial systemic understanding that goes beyond traditional approaches.

## Introduction

Alzheimer’s disease (AD) and Parkinson’s disease (PD) are the two most common neurodegenerative disorders (2),(3) and cumulatively affect over 400 million individuals worldwide (4),(5). Though significant genetic risk factors for AD and PD have been identified, sporadic and late-onset forms are thought to be caused by a complex interplay between genetic (6),(7) and environmental (8),(9) factors. This interplay underscores the imperative to explore a multitude of variables across bodily systems to comprehend their contributions to the etiology of AD/PD (10),(11).

Increasingly, research in neurodegeneration emphasizes the role of gut-brain axis health in neurodegeneration risk (12), (13). The gut-brain axis is a complex communication network that links the gastrointestinal tract and the central nervous system. This bidirectional system, including neural pathways, hormonal signaling, and immune mechanisms, facilitates constant interactions between the brain, digestive, endocrine, metabolic systems and nutritional status. Conditions that impact the gut-brain axis include digestive system disorders (14), endocrine pathway disorders (15), (16), nutritional deficiencies (17), (18), and metabolic traits (19).

Endocrine disorders, such as thyroid hormone imbalances, have been linked to AD and PD (16),(20), with conditions like hypothyroidism and subclinical hyperthyroidism being associated with dementia risk (21) and both hypo- and hyperthyroidism being associated with increasing PD risk (16). Metabolic disorders, particularly diabetes, are also related to neurodegenerative disease risk. An increased severity of diabetes is associated with a higher risk of PD (22),(23) and type 2 diabetes is a recognized risk factor for AD (24). Consequently, antidiabetic medications are being explored as potential treatments for AD and PD (25). Additionally, nutritional deficiencies such as low vitamin D levels are more prevalent in AD and PD patients (26). Digestive disorders have been observed to precede PD (27) or be significantly associated with an increased risk for dementia (28). These are just a few examples of factors contributing to the gut-brain axis health and their influence on neurodegeneration.

Understanding the connection between disorders of the gut-brain axis and neurodegeneration can provide useful insights into therapeutic interventions, with major implications for prevention and disease prognosis. In this study, we perform a large biobank-scale characterization of the impact that disorders affecting the gut-brain axis and related to endocrine, nutritional, metabolic, and digestive systems have on the risk of AD and PD. Utilizing data from the UK Biobank, SAIL, and FinnGen, we conducted a population-scale, unbiased assessment that aimed to: 1) Investigate the association between 155 diagnoses related to endocrine, nutritional, metabolic, and digestive system disorders and the risk of AD and PD, while also accounting for established genetic factors known to influence the development of these conditions; 2) Evaluate the specificity of plasma biomarkers associated with AD or PD when individuals have these co-occurring conditions; 3) Develop a multi-modal classification model combining all these data modalities.

## Methods

**Figure 1:**
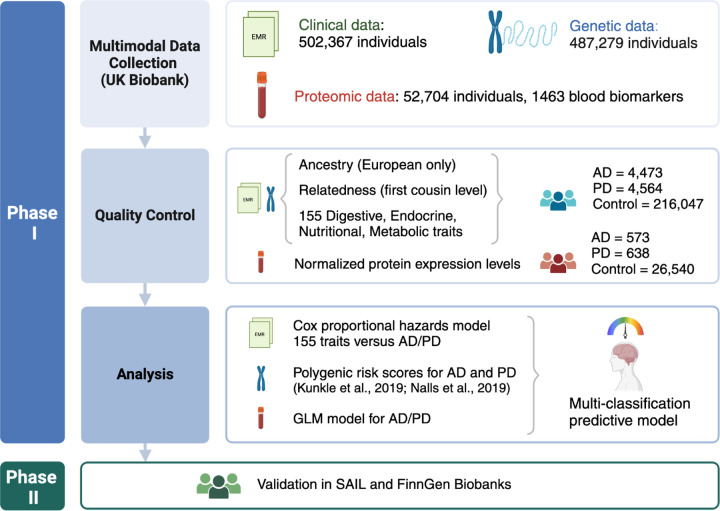
Study design The initial phase of our study utilized clinical data sourced from electronic medical records (EMR) alongside genetic and proteomic data obtained from the UK Biobank. Quality control procedures were rigorously applied to clinical and genetic datasets, including filtering for individuals of European ancestry, exclusion of related samples, and extraction of 155 ICD-10 codes representing diagnoses related to digestive, endocrine, nutritional, and metabolic disorders. Proteomic data underwent normalization of protein expression levels as part of quality control measures. The culmination of this phase involved the application of a Cox proportional hazards model, examination of polygenic risk scores, and development of a generalized linear model (GLM). These analyses collectively contributed to the construction of a multi-classification predictive model for Alzheimer’s disease (AD) and Parkinson’s disease (PD). Phase II of our study entailed validating these findings using data from the Secure Anonymised Information Linkage (SAIL) and FinnGen biobanks.

This study utilizes data from three biobanks: the UK Biobank (UKB), FinnGen, and the Secure Anonymized Information Linkage (SAIL) (See Figure 1 for an workflow rationale of this study).

### UK Biobank

The UKB data, accessed via DNAnexus under application number 33601, includes electronic health records of approximately 502,367 individuals, single nucleotide polymorphism (SNP) data of 487,279 individuals, and proteomic (Olink) data of 52,705 individuals (https://ukbiobank.dnanexus.com) (accessed on May 2023). The control group for the UKB dataset consisted of a subset of 352,610 individuals who have not been diagnosed with any neurodegenerative disease condition (Supplementary Table 1) and have no family history AD and PD diagnosis. For our approach, related individuals were filtered out from further analyses based on a kinship coefficient greater than 0.0884, and only individuals of European descent were selected for this study, based on field ID 22006, who self-identified as ‘White British’ and confirmed through principal component (PC) analysis of the genotyping data to avoid potential confounding effects. We lacked a sufficient number of individuals with non-European ancestry to conduct a meaningful assessment. Our final dataset included 409,520 individuals of European ancestry. Endocrine, nutritional, metabolic, and digestive disorders, as well as AD and PD diagnosis, were derived from ICD-10 (International Classification of Diseases, 10th Revision) codes. The AD cohort was obtained from the G30 and F00 ICD-10 codes in the UKB, and the PD cohort was obtained from the G20 ICD-10 code. We excluded any individuals who had received an ICD-10 diagnosis for endocrine, nutritional, metabolic, and digestive disorders before January 1st, 1999, and right-censored any individuals who received an ICD-10 diagnosis after being diagnosed with AD or PD. Furthermore, any ICD-10 code with fewer than 5 cases was excluded from the analysis. Demographic characteristics are shown in Supplementary Table 2.

### SAIL Databank

The Secure Anonymised Information Linkage (SAIL) databank is a virtual platform providing anonymized medical data of the population in Wales (29). Diagnoses in SAIL are sourced from the Patient Episode Database for Wales (PEDW), records from clinicians and hospital staff, the Welsh Longitudinal General Practitioner dataset (WLGP), records from primary care physicians of diagnoses, treatments, symptoms, and referrals. Demographic information such as sex, age, address, and death were obtained from the Welsh Demographic Services Database (WDSD) and WLGP. Individuals with missing age or sex data and those without a Welsh address were excluded from further analysis.

Diagnoses were identified in the PEDW using ICD-10 codes and in the WLGP using NHS read codes (CVT2,3). Neurodegenerative disorders from the outpatient data (OPDW) were excluded due to their minimal representation of dementia cases (only 0.1%) and the absence of reliable diagnosis dates. Similarly, dementia diagnoses from death records (ADDES) were not included due to inaccurate diagnosis dates. This study covered the period from January 1, 1999, to December 31, 2018. For inclusion, individuals were required to have been alive at the start of 1999 and to have been at least 45 years old on January 1, 1999.

### FinnGen Biobank

The FinnGen study is a large-scale genomics initiative that has analyzed over 500,000 Finnish biobank samples and correlated genetic variation with health data to understand disease mechanisms and predispositions. The project is a collaboration between research organisations and biobanks within Finland and international industry partners (1).

FinnGen provides survival analyses across numerous clinical endpoints. The hazard ratios are adjusted for sex and year of birth. FinnGen bases the calculation of these hazard ratios on a wide array of clinical endpoints defined through data from nationwide registries, including, but not limited to, Statistics Finland [https://finngen.gitbook.io/documentation/methods/endpoints]. We downloaded the hazard ratios for AD/PD from FinnGen’s Risteys R10 platform for AD using the G6_AD_WIDE category matching more closely the UKB grouping than G6_AD [https://r10.risteys.finngen.fi/endpoints/G6_AD_WIDE] and for PD [https://r10.risteys.finngen.fi/endpoints/G6_PARKINSON] to explore the putative impact of endocrine, digestive, metabolic, and nutritional disorders on the risk of of AD/PD prior to diagnosis. It is important to note that not all ICD-10 codes used in our discovery phase in the UKB are represented in the FinnGen dataset. This discrepancy is perhaps due to the differences in health registries and data collection methodologies between the UKB and FinnGen biobanks.

#### Polygenic Risk Score Analyses

Risk allele loci and beta values from GWAS summary statistics, specifically 23 risk predictors linked to AD risk from Kunkle 2019 and 90 risk predictors associated with PD from Nalls 2019, were used to estimate PRS for AD and PD respectively (30), (31). Each risk allele was assigned a weight based on the magnitude of its effect in the published studies, giving greater emphasis to alleles with higher risk estimates. Genetic variants were extracted for each individual from the UKB imputed data using the ‘bgenix’ package (32). The extracted variants were then converted to binary formats and used to compute the PRS score for each individual using PLINK 2.0 (33). Estimated profiles were then normalized to Z-scores based on a control reference.

#### Cox Proportional Hazard Model

A Cox proportional hazards model was used to calculate the hazard ratio between risk for incident AD and PD and endocrine, nutritional, metabolic, and digestive system disorders. The disorders under study are represented as 155 ICD-10 codes (Supplementary Table 3). In this model, January 1st, 1999 was used as the cutoff date for the diagnosis of these conditions. Consequently, any individual diagnosed with these traits before January 1st, 1999 was excluded from the analysis. Logistic regressions and Cox proportional hazards models were adjusted for age, sex, the Townsend deprivation index, and 5 PCs to account for population stratification (precomputed in UKB only). Additionally, we conducted Time-Stratified Cox Regression Analysis, for which cohorts were divided into three strata based on ICD-10 codes: 1–5 years, 5–10 years, and 10–15 years prior to NDD diagnosis. ICD-10 diagnoses within the specified time periods were retained, while any ICD-10 codes outside of these time frames were converted to NaN.

#### Polygenic Risk Score distribution for Alzheimer’s disease and Parkinson’s disease

A t-test was used to quantify the differences in the distribution of genetic alleles for AD or PD between individuals diagnosed only with AD or PD and individuals with an additional, significant ICD-10 diagnosis. This approach measures how much the cumulative effect of these alleles varies between the two groups. Additionally, it unveils the genetic risk profiles for AD and PD in both groups. By calculating and comparing PRSs based on alleles associated with AD or PD, the analysis evaluates how the genetic predisposition to AD or PD is increased or decreased among those individuals diagnosed with one of the ICD-10 codes in addition to AD or PD. This comparison aids in understanding whether genetic risk for AD or PD is similar or distinct in the compared subgroups of study.

#### Statistical Analysis accounting for the Impact of *APOE*, *LRRK2*, and *GBA1* risk variants.

PRS analyses were adjusted for major genetic risk factors associated with AD and PD to determine if observed similarities or differences between AD/PD and the assessed ICD-10 codes could be attributed to pleiotropic effects. For AD, individuals homozygous for the ‘C’ allele at both *APOE* rs429358 and rs7412 were identified as having two copies of the *APOE*-ε4 allele, coded as ‘2’ , and one copy of the *APOE*-ε4 allele coded as ‘1’ in our regression analysis to indicate a higher genetic risk. All other configurations were coded as ‘0’. For PD, we examined *LRRK2* at rs76904798, where homozygous ‘C’ alleles were coded as ‘0’, and heterozygous and homozygous for ‘T’ alleles as ‘1’. Similarly, at rs34637584 (*LRRK2* G2019S), we applied ‘0’ for homozygous ‘G’ alleles, and ‘1’ for heterozygous and homozygous ‘A’ alleles. For *GBA1*, at rs35749011 (proxy for *GBA1* E326K), ‘0’ was assigned to homozygous ‘G’ alleles, ‘1’ to heterozygous and homozygous ‘A’ alleles, and at rs76763715 (*GBA1* N370S), ‘0’ for homozygous ‘T’, and ‘1’ for heterozygous and homozygous ‘C’ alleles.

#### Interaction model for genetic risk across endocrine, metabolic, digestive system disorders and nutritional status

We aimed to understand how the interplay between genetics underlying AD and PD risk and a clinical diagnosis for any investigated ICD-10 code could eventually influence an AD or PD diagnosis. A Generalized Linear Model (GLM) was used to account for more complex relationships, where the impact of genetic risk for AD or PD (as measured by PRS) might interact differently across the diagnoses under study represented by ICD-10 codes. The interaction term used in this model was: Z-score * ICD-10 terms adjusted by sex, age and Townsend deprivation index.

#### Proteomic Biomarker Data Analyses

We aimed to explore differences in the levels of proteomic biomarkers associated with AD or PD between individuals with and without co-occurring ICD-10 code diagnoses related to endocrine, nutritional, metabolic, and digestive system disorders among UKB participants. For this purpose, we utilized data from the Pharma Proteome Project, which provides thousands of plasma protein biomarkers in blood samples (https://olink.com/news/uk-biobank-pharma-proteomics-project/). In a cross-sectional analysis, we focused on baseline (Instance_0) proteomics data, as it includes measurements for the largest number of individuals, encompassing data for 52,705 individuals (at the time of data access) and a total of 1,463 proteins (Supplementary Table 4). These biomarkers span across cardiometabolics, inflammation, neurology, and oncology markers.

We applied GLM to analyze 1,463 available biomarkers on AD and PD risk. The regressions were adjusted for age at recruitment, Townsend deprivation index, sex, and 5 PCs. Subsequently, we selected False Discovery Rate (FDR)-corrected significant proteomic biomarkers from the GLM results. Additionally, we compared the average levels of proteomic biomarkers in isolated cases of AD/PD with cases of AD/PD co-occurring with selected ICD-10 codes (found to be significantly associated with AD or PD risk before AD/PD diagnosis). We selected FDR-corrected significant proteomic biomarkers with an OR greater than 1 and applied a t-test to compare their average levels in the isolated cases of AD or PD and cases co-occurring with specific ICD-10 codes.

#### Multi-modal classification model for clinical, genetics and proteomics on Alzheimer’s disease and Parkinson’s disease risk

To evaluate the role of endocrine, metabolic, digestive and nutritional status-related diagnoses in predicting AD or PD status, we developed a multi-modal classification model on a subset of the UK Biobank dataset. This subset included individuals with variables encompassing clinical diagnosis for endocrine, metabolic, digestive and nutritional status; biomarkers; demographic factors (age at recruitment, sex, and Townsend deprivation index); derived variables (Z score for the PRS); PCs; and *APOE* status for AD, or *LRRK2*/*GBA1* status for the PD model. The clinical diagnoses used in these models include the ICD-10 codes found to have significant HRs in UKB. We employed a machine learning approach to compare the predictive performance of various feature sets on AD/PD outcomes. These included genetics, clinical, proteomic, demographics (including sex, age at recruitment), and combinations thereof. Each dataset was independently analyzed using a Gradient Boosting Classifier, an ensemble learning method for classification tasks (https://scikit-learn.org/stable/modules/generated/sklearn.ensemble.GradientBoostingClassifier.html). We conducted hyperparameter tuning through GridSearchCV, optimizing for the number of estimators, learning rate, and maximum depth. For AD classification, the grid search adjusts XGBClassifier parameters including n_estimators (2, 3, 5, 10, 15), learning_rate (0.001, 0.01, 0.1), and max_depth (3, 4, 5). For PD, it adjusts n_estimators (300, 500), learning_rate (0.005, 0.01), and max_depth (1, 2, 3). Both models use nested cross-validation, optimizing based on ROC AUC. The dataset was downsampled to have an equal number of cases and controls, and then scaled using StandardScaler to ensure uniformity and prevent bias due to variance in measurement scales before classification training. Model performance was evaluated based on ROC AUC and balanced accuracy (BA) scores.

## Results

### A prior diagnosis of certain endocrine, nutritional, metabolic, and digestive system related disorders is associated with increased risk for Alzheimer’s disease and Parkinson’s disease

Cox regression models unraveled a total of 16 ICD-10 diagnoses to be significantly associated with the risk of AD (Supplementary Table 5 and Supplementary Figure 1) in the UKB after correction for multiple comparisons (discovery cohort) that were also found to be significant in either SAIL, FinnGen biobanks, or both (Table 1). A diagnosis of hemorrhoids and perianal venous thrombosis was found to have a HR<1 for AD in all three datasets. This observation could potentially be due to the fact that a hospitalized diagnosis of hemorrhoids and perianal venous thrombosis could be an indication of other, more serious conditions linked to a high mortality rate, thus explaining the protective observed effect (34). The 15 other ICD-10 codes that were found to be significant have a HR>1, suggesting that being diagnosed with these conditions increases the risk for AD; these include amyloidosis; diseases of pulp and periapical tissues; disorders of lipoprotein metabolism and other lipidemias; disorders of mineral metabolism; gastritis and duodenitis; insulin-dependent, non-insulin-dependent, and unspecified diabetes mellitus; oesophagitis; other bacterial intestinal infections; other disorders of fluid, electrolyte and acid-base balance; other functional intestinal disorders; other noninfective gastro-enteritis and colitis; and volume depletion.

Of note, our analyses indicate significant associations between 7 disorders and the risk for PD (Supplementary Table 6 and Supplementary Figure 2) that were replicated in either SAIL or FinnGen biobanks (Table 2). For PD, diverticular disease of the intestine, other diseases of the intestine, and other disorders of the peritoneum have HR<1 replicated in two datasets. Similarly to AD, individuals diagnosed with these conditions are not representative of the entire population due to the severity of these conditions and differential survival rates (36–39). The other 7 ICD-10 codes that had a HR>1 include dyspepsia; insulin-dependent and non-insulin-dependent diabetes mellitus; and other functional intestinal disorders.

Additionally, we conducted a time-stratified cox regression analysis to evaluate whether the timing of diagnosis for the ICD-10 codes under study impacts HR values for AD or PD. We split the samples from UKB into three strata: 1–5 years, 5–10 years, and 10–15 years prior to AD/PD diagnosis. We then re-evaluated the HRs for AD and PD for the significant ICD-10 codes identified in the previous analysis (Table 3 and Table 4). The values and directions of the HRs in the stratified analysis show that the risk associated with each ICD-10 code is consistent across the three strata.

### Survival analysis indicates increased Alzheimer’s disease and Parkinson’s disease incidence in individuals with significant diagnosis of endocrine, nutritional, metabolic, and digestive system related disorders

Using UKB data, we conducted survival analyses to explore the probabilities of an AD and PD diagnosis at a certain time interval. We generated Kaplan-Meier plots for the significantly associated ICD-10 codes as depicted in Supplementary Figure 3 and Supplementary Figure 4, respectively. At the beginning of the observation period, the survival probability starts at 1.0, indicating that all individuals diagnosed with an ICD-10 code are initially free from AD and PD. Over time, this probability diminishes as more individuals are diagnosed with AD or PD. The curves demonstrate the impact of specific ICD-10 code diagnosis on the likelihood of developing these neurodegenerative diseases. Notably, individuals with the significantly associated ICD-10 code diagnoses exhibited a higher incidence of AD and PD.

### Genetic susceptibility for Alzheimer’s disease and Parkinson’s disease is higher in isolated cases compared to those with co-occurring endocrine, nutritional, metabolic, and digestive disorders

We compared the distribution of PRS in individuals diagnosed only with AD or PD versus those having concurrent AD/PD and any ICD-10 code diagnosis under study for samples from UKB only. Of note, some significant ICD-10 codes associated with AD included those pertaining to diabetes mellitus; other disorders of fluid, electrolyte, and acid-base balance; and obesity. Similarly, there were significant associations between diabetes mellitus, disorders of the peritoneum, and vitamin B group deficiencies, and PD. A comprehensive summary of these results is shown in Supplementary Figure 5 and Supplementary Figure 6.

For all significant t-tests, a lower average PRS was observed in individuals with co-occurring AD and another condition compared to individuals with only AD. For instance, in AD patients with a non-insulin-dependent diabetes mellitus diagnosis, our analysis revealed lower average PRS scores compared to individuals with only AD, either including or excluding *APOE* (t-test = −4.26, P = 2.3e-5 and t-test = −2.88, P = 4.01e-3, respectively). Similar trends were observed in individuals with AD and other bacterial intestinal infections (including *APOE* t-test = −2.31, P = 2.24e-02; excluding *APOE* t-test = −2.26, P = 2.54e-02), other disorders of pancreatic internal secretion (including *APOE* t-test = −2.17, P = 3.12e-02), oesophagitis (including *APOE* t-test = −3.08, P = 2.33e-02; excluding *APOE* t-test = −2.39, P = 1.78e-02), as well as gastritis and duodenitis (including *APOE* t-test = −2.83, P = 4.76e-03).

Of note, lower average PRS was observed for all significant t-tests in individuals with co-occurring PD and another condition compared to individuals with only PD. For instance, PD patients with other disorders of the peritoneum had a lower average PRS score than those with only PD (t-test = −3.00, P = 3.70e-03). Additionally, individuals with PD and other functional intestinal disorders (t-test = −2.08, P = 3.80e-02), insulin-dependent diabetes (t-test = −2.55, P = 1.29e-02), non-insulin dependent diabetes (t-test = −3.68, P = 2.52e-4), or a deficiency of other B group vitamins (t-test = −2.54, P = 1.23e-02) also had lower average PRS scores than individuals with only PD.

This result must be interpreted with caution, as there is potential for collider bias in our analysis. By selecting individuals with AD/PD, we are conditioning on these disorders, which are influenced by both the PRS for AD/PD and other risk factors, such as co-occurring endocrine, nutritional, metabolic, and digestive disorders. This conditioning can induce a spurious association between the PRS for AD/PD and these other risk factors, leading to the appearance of a lower average PRS in the group with AD/PD and co-occurring disorders. Essentially, this bias might make it seem as though lower genetic risk is linked to AD/PD in the presence of ICD-10 diagnoses, when in fact the relationship may be driven by the complex interplay of risk factors.

### The interplay between AD and PD cumulative known genetic effects and the risk for endocrine, nutritional, metabolic, and digestive systems disorders did not display any synergistic impact

We did not identify significant synergistic interaction terms at a nominal P<0.05 and exhibiting an OR>1 in either AD (see Supplementary Table 7 and Supplementary Table 8) or PD (see Supplementary Table 9). This suggests that the combined occurrence of endocrine, nutritional, metabolic, or digestive system related disorders does not lead to a synergic effect on AD/PD risk that surpasses the sum of their individual impacts.

### The proteomic profile of individuals diagnosed with Alzheimer’s disease and Parkinson’s disease is influenced by comorbid endocrine, nutritional, metabolic, and digestive systems conditions

Our analysis showed 22 proteomic biomarkers with notable differences in AD cases versus controls, alongside 156 proteins exhibiting significant distinctions in PD cases compared to controls after controlling for multiple comparisons (Supplementary Table 10 and Supplementary Table 11, respectively). We delved deeper into the levels of proteomic biomarkers, applying a t-test to juxtapose average levels between standalone cases of AD/PD and cases co-occurring with specific ICD-10 codes. After implementing FDR corrections, we found significant differences in proteomic biomarker levels in individuals with only AD/PD compared to individuals with AD/PD and co-occurring ICD-10 conditions. Specifically, we observed that 37 biomarkers displayed increased levels in AD cases with co-occurring ICD-10 diagnosis compared to isolated AD cases (see Table 5), while a total of five biomarkers demonstrated significant elevated levels in PD cases with accompanying conditions compared to standalone PD cases (Table 6). These findings hint at distinct associations between specific biomarkers and AD/PD, while others may be influenced by concurrent comorbidities.

### Multi omics integration models based on clinical, genetic, and proteomic data improve accuracy to predict Alzheimer’s disease and Parkinson’s disease risk versus a single paradigm

**Figure 2:**
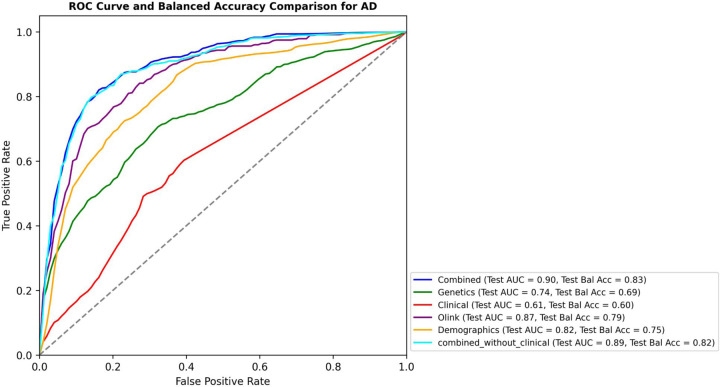
Performance evaluation of multi-omics integration models using clinical, genetic, proteomic, and demographic data for Alzheimer’s disease

In our analysis, the combination of genetic, clinical, proteomic (controlling for multiple comparisons using Bonferroni correction), and demographic factors (including age at recruitment and gender) exhibited superior predictive performance for AD risk when compared to the individual data sets, with a test AUC of 0.9 and test balanced accuracy (BA) of 0.83. Curiously, the model combining genetic, proteomic, and demographic features (i.e. no clinical information) had a very similar test AUC of 0.89 and a test BA of 0.82. The best classifier using only one data attribute was the model that used only proteomic data for prediction with a test AUC of 0.87 and test BA of 0.79. The performance of each predictive model is shown in Figure 2 and the metrics are listed in Supplementary Table 12.

**Figure 3:**
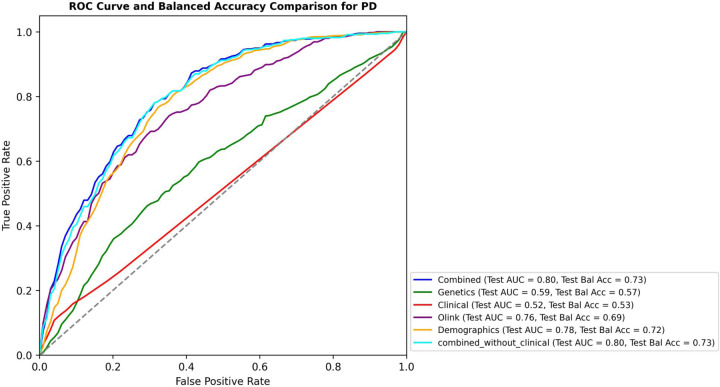
Performance evaluation of multi-omics integration models using clinical, genetic, proteomic, and demographic data for Parkinson’s disease

For PD, the integration of genetic, proteomic and demographic factors showcased heightened predictive efficacy with a test AUC of 0.80 and test BA of 0.73. For PD, addition of the clinical data did not improve the AUC and test BA of the model. The best classifier using only one data attribute was the model that used demographics data (Age at recruitment, Sex and Townsend_deprivation_index), with a test AUC of 0.78 and a test BA of 0.72. The performance of each model for PD is shown in Figure 3 and Supplementary Table 13.

## Discussion

With the increasing prevalence of AD (40) and PD (41), it is imperative to enhance our understanding of the determinants that increase the risk for these common neurodegenerative diseases and most importantly, develop improved prediction models for early detection. Here, we have undertaken the most extensive biobank-scale omics study to date to assess the influence of main biological system disorders implicated in the gut-brain axis (including endocrine, nutritional, metabolic, and digestive-related conditions) preceding the diagnosis of AD and PD. The culmination of which is a multimodal classification model that combines clinical, genetics, and proteomics data enhancing the prediction accuracy of AD and PD.

In a large-scale and data driven manner, we demonstrate that certain endocrine, nutritional, metabolic, and digestive system related disorders are significantly associated with an increased risk of AD and/or PD prior to diagnosis. Of note, individuals with other non-infective gastroenteritis and colitis; oesophagitis; gastritis and duodenitis; disorders of fluid, electrolyte, and acid-base balance; pancreatic internal secretion disorders; and other functional intestinal disorders showed a higher likelihood of developing AD later in life. Recent literature has suggested the amplification of AD risk from disorders affecting the gut-brain axis, such as gastritis (42). Our results corroborate these findings and reveal additional, novel potential disorders of interest for further study, with replication across multiple datasets. In regards to PD, significant diagnoses associated with increased PD risk include other functional intestinal disorders, disorders of pancreatic internal secretion, and deficiency of other B group vitamins. The correlation between the deficiency of other B group vitamins and PD expands upon previous studies that have looked at other vitamin deficiencies, such as vitamin D (26), and warrants more research on the impact of nutritional deficiencies on neurodegenerative diseases.

Our study robustly demonstrates that the probability of developing AD/PD increases with certain co-occurring endocrine, metabolic, digestive, or nutritional conditions, corroborating the hypothesis that a diagnosis affecting the gut-brain axis elevates the risk of AD/PD. We also show that risk of neurodegeneration persists up to 15 years before AD/PD onset with a co-occurring diagnosis of an endocrine, metabolic, digestive or nutritional disorder or trait. For example, having a diagnosis of other functional intestinal disorders results in an increased HR for both AD and PD in the periods 1–5, 5–10, and 10–15 years prior to AD/PD diagnosis.

In an effort to investigate genetic distinctions and potential etiological subtypes of AD and PD, we compared polygenic risk for AD and PD in individuals diagnosed only with AD or PD and individuals with AD or PD co-occurring with other endocrine, nutritional, metabolic, and digestive-related disorders. Of note, our study confirmed that individuals diagnosed with any type of diabetes mellitus in addition to AD or PD are shown to have a significant different PRS than individuals with AD or PD alone, in concordance with previous studies showing that diabetes is a risk factor for both AD and PD (43–45). For both AD and PD, comorbidity with any of the significant disorders affecting the gut-brain axis showed lower average PRS scores compared to those with only AD or PD. Our findings suggest that systemic health risk factors can more prominently account for one’s disease risk in the absence of other genetic risk factors in patients with AD or PD, highlighting the importance of considering both genetic as well as other health factors in assessing the overall risk of developing AD and PD. Using diabetes as a positive control in our study, we found that individuals diagnosed with AD along with another disorder affecting the gut-brain axis, such as other bacterial intestinal infections and other functional intestinal disorders, result in a significantly lower average PRS score compared to individuals with only AD. This suggests that severe digestive conditions such as infections affecting the gut microbiome, independent of genetic risk, could increase the risk for AD as previously described (46). For PD, there existed a significant difference in PRS in individuals without vs. with other functional intestinal disorders and other disorders of the peritoneum, in concordance with previous studies suggesting an influence of disorders involving the gut-brain axis on PD (47).

In addition, our analysis revealed that the relationship between the genetic risk for AD/PD and many disorders of the endocrine, nutritional, metabolic and digestive systems resulted in a combined impact not significantly greater than the sum of their individual effects. These interactions are not synergistic, confirming the notion that known genetic risk factors included in the models under study for both AD and PD are independent from gastrointestinal and metabolic disorders, which highlights the importance of environmental factors in the development of both AD and PD. While both genetic and systemic health disorders independently influence the risk of AD and PD, many disorders may not interact in a way that significantly amplifies this risk when combined.

Through our exploration of proteomics and AD/PD risk, we identified several promising candidates for AD/PD diagnosis. For instance, we found that for AD, the proteomic biomarker with the largest impact on disease was glial fibrillary acidic protein (GFAP), supporting previous literature findings that GFAP can serve as an indicator of AD pathology (48). However, the levels of GFAP were significantly decreased in samples with AD and a co-occurring ICD-10 code for gastritis and duodenitis or non-insulin dependent diabetes mellitus. For PD, in concordance with previous studies, we identified peroxiredoxin 1 (PRDX1) as a potential biomarker for disease (49). Again, the levels of PRDX1 were significantly decreased in PD cases and a coinciding ICD-10 code for insulin-dependent diabetes mellitus. These differences in biomarker levels between samples solely with AD/PD vs. samples with additional diagnoses highlight the influence of comorbidities on disease manifestation. Although longitudinal research would need to be conducted, our findings suggest that these biomarkers could serve as valuable diagnostic or prognostic tools for AD and PD, potentially enhancing early detection and disease management.

The inclusion of multiple features, integrating clinical data on digestive, endocrine, nutritional and metabolic disorders, genetic risk scores, and proteomic data in our prediction model, demonstrated superior performance in predicting both AD and PD compared to single-variable paradigms. Co-occurring diagnosis for conditions influencing the gut-brain axis do not seem to influence the predictability of neither AD nor PD as much as the other variables (demographic factors, biomarkers, and genetic status), but the fact that for both AD and PD the combination of data (clinical, genetic, proteomic, and demographic) produces a higher AUC for the ROC curve compared to single modalities underscores the value in including multiple facets of data in predictive models. Overall, these results from our models reveal the promising predictive capabilities of our constructed multi-modal classification models in identifying individuals at risk for AD/PD. The study suggests that the selected proteomic biomarkers, when combined with genetic and other demographic and clinical variables, serve as robust predictors. These predictors have the potential to significantly enhance prediction diagnostic accuracy for AD/PD. Our approach highlights the potential clinical utility of multi-omics in enhancing diagnostic accuracy, and further emphasizes the importance of considering other bodily systems when predicting the risk underlying neurodegenerative diseases.

Some overall limitations of our work include the use of solely ICD-10 codes for diagnosis, and not other additional assays, which may overlook undiagnosed cases, leading to a potential underestimation of the true impact of these disorders on AD/PD risk. Across datasets, the available diagnosis codes also differ causing further limitations in comparison and validation. Because individuals with AD often present with different cognitive, behavioral, and pathological features, diagnosis of AD is often difficult and inconsistent (50). Though we use the ICD-10 code to filter for AD in this study, it is possible for AD to be interchanged with dementia as diagnosing AD is difficult. The sample makeup across data sources can also be different. Since the UKB participants are volunteers who may have agreed to participate before reaching old age, the incidence of AD/PD may be different compared to the SAIL and FinnGen datasets. The cross-sectional nature of the data used to study biomarker associations may limit any causal inference. The SAIL and FinnGen datasets do not include proteomic data, preventing us from further validating our modeling efforts. The focus on samples of European ancestry constrains the generalizability of our findings to other populations from other genetic backgrounds. Future work needs to be done across different ancestral backgrounds to be globally representative.

Our study delves into the intricate interactions between clinical, genetic and proteomic data, culminating in the construction of a comprehensive multi-modal classification model. This pioneering endeavor aims to shed light on the nuanced interplay between various physiological factors playing a role on the gut-brain axis and the development of AD and PD, offering a multifactorial systemic understanding that transcends traditional approaches. Our integrated approach serves as a proof of concept, aligning with the expanding body of evidence that underscores the intricate etiological foundations of neurodegenerative diseases and holds promise for refining risk prediction models and devising targeted preventive strategies. This, in turn, propels our endeavors in elucidating clinical interventions aimed at addressing these debilitating conditions.

## Supplementary Material

Supplement 1 Supplementary Figure 1 Forest plots displaying the impact of digestive, endocrine, metabolic and nutritional conditions on Alzheimer’s disease risk prior to diagnosis

Supplement 2 Supplementary Figure 2 Forest plots displaying the impact of digestive, endocrine, metabolic and nutritional conditions on Parkinson’s disease risk prior to diagnosis

Supplement 3 Supplementary Figure 3 Survival analysis: Alzheimer’s disease risk in individuals with digestive, endocrine, metabolic and nutritional related conditions

Supplement 4 Supplementary Figure 4 Survival analysis: Parkinson’s disease risk in individuals with digestive, endocrine, metabolic and nutritional related conditions

Supplement 5 Supplementary Figure 5 Distribution of polygenic risk scores in individuals diagnosed only with Alzheimer’s disease versus those diagnosed with concurrent Alzheimer’s disease and significant digestive, endocrine, metabolic and nutritional conditions under study

Supplement 6 Supplementary Figure 6 Distribution of polygenic risk scores in individuals diagnosed only with Parkinson’s disease versus those diagnosed with concurrent Parkinson’s disease and significant digestive, endocrine, metabolic and nutritional conditions under study

Supplement 7 Supplementary Figure 7 Polygenic risk score distribution for Alzheimer’s disease (all significant ICD-10 codes)

Supplement 8 Supplementary Figure 8 Polygenic risk score distribution for Parkinson’s disease (all significant ICD-10 codes)

Supplement 9 Supplementary Table 1 ICD 10 codes used to create control cohort
Supplementary Table 2 Biobank cohort gender and Alzheimer’s disease/Parkinson’s disease demographics
Supplementary Table 3 UKB diagnosis and disorders under study
Supplementary Table 4 Proteomic biomarkers under study
Supplementary Table 5 Cox proportional hazards regression analysis of Alzheimer’s disease and endocrine, nutritional, metabolic, and digestive system disorders ICD-10 codes adjusted for year of birth, Townsend deprivation index, and sex
Supplementary Table 6 Cox proportional hazards regression analysis of Parkinson’s disease and endocrine, nutritional, metabolic, and digestive system disorders ICD-10 codes adjusted for year of birth, Townsend deprivation index, and sex
Supplementary Table 7 Interaction terms between endocrine, nutritional, metabolic, digestive system disorders and Alzheimer’s disease polygenic risk score (excluding *APOE*)
Supplementary Table 8 Interaction terms between endocrine, nutritional, metabolic, and digestive system disorders and Alzheimer’s disease polygenic risk score
Supplementary Table 9 Interaction terms between endocrine, nutritional, metabolic, digestive system disorders and Parkinson’s disease polygenic risk score
Supplementary Table 10 Biomarkers associated with Alzheimer’s disease
Supplementary Table 11 Biomarkers associated with Parkinson’s disease
Supplementary Table 12 Performance summary of different feature sets for Alzheimer’s disease classification
Supplementary Table 13 Performance summary of different feature sets for Parkinson’s disease classification
Supplementary Table 14 Cox proportional hazards regression analysis of Alzheimer’s disease and endocrine, nutritional, metabolic, and digestive system disorders ICD-10 codes adjusted for *APOE 4/4* status, principal components 1–5, year of birth, Townsend deprivation index, and sex
Supplementary Table 15 Cox proportional hazards regression analysis of Alzheimer’s disease and endocrine, nutritional, metabolic, and digestive system disorders ICD-10 codes adjusted for polygenic risk Z-score excluding *APOE*, *APOE* 4/4 status, principal components 1–5, year of birth, Townsend deprivation index, and sex
Supplementary Table 16 Cox proportional hazards regression analysis of Alzheimer’s disease and endocrine, nutritional, metabolic, and digestive system disorders ICD-10 codes adjusted for polygenic risk Z-scores excluding *APOE*, principal components 1–5, year of birth, Townsend deprivation index, and sex
Supplementary Table 17 Cox proportional hazards regression analysis of Alzheimer’s disease and endocrine, nutritional, metabolic, and digestive system disorders ICD-10 codes adjusted for polygenic risk Z-score, *APOE 4/4* status, principal components 1–5, year of birth, Townsend deprivation index, and sex
Supplementary Table 18 Cox proportional hazards regression analysis of Alzheimer’s disease and endocrine, nutritional, metabolic, and digestive system disorders ICD-10 codes adjusted for polygenic risk Z-scores, principal components 1–5, year of birth, Townsend deprivation index, and sex
Supplementary Table 19 Cox proportional hazards regression analysis of Alzheimer’s disease and endocrine, nutritional, metabolic, and digestive system disorders ICD-10 codes adjusted for principal components 1–5, year of birth, Townsend deprivation index, and sex
Supplementary Table 20 Cox proportional hazards regression analysis of Parkinson’s disease and endocrine, nutritional, metabolic, and digestive system disorders ICD-10 codes adjusted for principal components 1–5, year of birth, Townsend deprivation index, and sex
Supplementary Table 21 Cox proportional hazards regression analysis of Parkinson’s disease and endocrine, nutritional, metabolic, and digestive system disorders ICD-10 codes adjusted for polygenic risk Z-scores, principal components 1–5, Townsend deprivation index, and sex
Supplementary Table 22 Cox proportional hazards regression analysis of Parkinson’s disease and endocrine, nutritional, metabolic, and digestive system disorders ICD-10 codes adjusted for *GBA1* (G_1_155162560 + T_1_155235843) status, principal components 1–5, year of birth, Townsend deprivation index, and sex
Supplementary Table 23 Cox proportional hazards regression analysis of Parkinson’s disease and endocrine, nutritional, metabolic, and digestive system disorders ICD-10 codes adjusted for polygenic risk Z-scores, *GBA1* (G_1_155162560 + T_1_155235843) status, principal components 1–5, year of birth, Townsend deprivation index, and sex
Supplementary Table 24 Cox proportional hazards regression analysis of Parkinson’s disease and endocrine, nutritional, metabolic, and digestive system disorders ICD-10 codes adjusted for *LRRK2* (C_12_40220632 + G_12_40340400) status, principal components 1–5, year of birth, Townsend deprivation index, and sex
Supplementary Table 25 Cox proportional hazards regression analysis of Parkinson’s disease and endocrine, nutritional, metabolic, and digestive system disorders ICD-10 codes adjusted for polygenic risk Z-scores, *LRRK2* (C_12_40220632 + G_12_40340400) status, principal components 1–5, year of birth, Townsend deprivation index, and sex

## Figures and Tables

**Table 1.** Replicated diagnoses in endocrine, nutritional, metabolic, and digestive systems associated with AD risk

Prior_ICD10_code	Prior_ICD10_code_Description	Dataset	Neurodegenerative disease outcome	Hazard Ratio	ci_min	ci_max	P_VAL	P_VAL_FDR_CORRECTED	N_pairs	n	rejected
E85	amyloidosis, other/unspecified	Finngen	AD	3.98	1.20	13.27	2.44E-02	3.65E-02	17		TRUE
	amyloidosis	UKB		2.57	1.34	4.94	4.72E-03	2.61E-02	9	182	TRUE
K04	diseases of pulp and periapical tissues	Finngen	AD	2.83	1.67	4.82	1.17E-04	2.68E-04	267		TRUE
	diseases of pulp and periapical tissues	UKB		1.44	1.11	1.87	6.21E-03	3.26E-02	57	3,365	TRUE
E78	disorders of lipoprotein metabolism and other lipidaemias	Finngen	AD	5.71	4.76	6.85	1.55E-78	1.46E-76	2,522.00		TRUE
	disorders of lipoprotein metabolism and other lipidaemias	UKB		1.18	1.09	1.27	1.53E-05	1.34E-04	1090	53,797	TRUE
E83	disorders of mineral metabolism	Finngen	AD	6.63	3.51	12.51	5.26E-09	2.10E-08	68.00		TRUE
	disorders of mineral metabolism	UKB		1.32	1.08	1.62	7.75E-03	3.88E-02	94	4,435	TRUE
K29	other gastritis (incl. Duodenitis)	Finngen	AD	2.40	1.53	3.77	1.35E-04	3.06E-04	416.00		TRUE
	gastritis and duodenitis	UKB		1.20	1.09	1.32	1.45E-04	1.17E-03	523	27,914	TRUE
K64	haemorrhoids and perianal venous thrombosis	Sail	AD	0.37	0.27	0.51	6.83E-10	8.64E-09	39	11,389	TRUE
	haemorrhoids and perianal venous thrombosis	UKB		0.69	0.59	0.80	6.94E-07	8.10E-06	186	20,162	TRUE
E10	Type 1 diabetes, wide definition * (includes E10)	Finngen	AD	2.46	1.63	3.70	1.60E-05	4.22E-05	272		TRUE
	insulin-dependent diabetes mellitus	Sail		1.78	1.48	2.13	4.29E-10	5.68E-09	118	6,668	TRUE
	insulin-dependent diabetes mellitus	UKB		3.09	2.45	3.90	1.77E-21	1.86E-19	73	1,633	TRUE
E11	Type 2 diabetes, wide definition*(includes E11)	Finngen	AD	4.02	3.28	4.92	2.54E-41	8.54E-40	2641		TRUE
	non-insulin-dependent diabetes mellitus	Sail		1.27	1.19	1.35	3.77E-14	8.43E-13	1052	72,913	TRUE
	non-insulin-dependent diabetes mellitus	UKB		1.47	1.33	1.63	1.19E-14	4.18E-13	476	19,048	TRUE
K20	oesophagitis	Finngen	AD	4.23	2.20	8.13	1.58E-05	4.16E-05	77.00		TRUE
	oesophagitis	UKB		1.28	1.10	1.49	1.63E-03	1.00E-02	175	8,660	TRUE
A04	other bacterial intestinal infections	Finngen	AD	2.56	1.22	5.37	1.27E-02	2.01E-02	255.00		TRUE
	other bacterial intestinal infections	Sail		1.37	1.14	1.65	6.29E-04	2.95E-03	117	6,202	TRUE
	other bacterial intestinal infections	UKB		1.51	1.21	1.89	2.35E-04	1.76E-03	81	3,585	TRUE
E87	other disorders of fluid, electrolyte and acid-base balance	Finngen	AD	10.04	7.24	13.93	2.11E-43	8.07E-42	600.00		TRUE
	other disorders of fluid, electrolyte and acid-base balance	UKB		1.51	1.34	1.70	6.43E-12	1.35E-10	312	11,313	TRUE
K59	other functional intestinal disorders	Finngen	AD	4.37	3.30	5.80	1.02E-24	1.51E-23	886.00		TRUE
	other functional intestinal disorders	Sail		1.19	1.09	1.29	9.59E-05	5.47E-04	529	29,639	TRUE
	other functional intestinal disorders	UKB		1.55	1.40	1.73	3.55E-16	1.86E-14	394	15,596	TRUE
K52	other noninfective gastroenteritis and colitis	Finngen	AD	2.53	1.40	4.57	2.15E-03	3.98E-03	300.00		TRUE
	other non-infective gastro-enteritis and colitis	Sail		1.36	1.22	1.50	4.97E-09	5.56E-08	373	23,128	TRUE
	other non-infective gastro-enteritis and colitis	UKB		1.37	1.20	1.56	1.75E-06	1.84E-05	250	12,984	TRUE
E14	unspecified diabetes mellitus	Sail	AD	1.49	1.19	1.88	6.05E-04	2.89E-03	73	4,470	TRUE
	unspecified diabetes mellitus	UKB		1.53	1.33	1.75	1.21E-09	1.58E-08	224	8,733	TRUE
E55	vitamin d deficiency	Finngen	AD	13.48	4.76	38.22	9.94E-07	3.16E-06	26.00		TRUE
	vitamin d deficiency	UKB		1.95	1.57	2.41	9.97E-10	1.50E-08	87	2,405	TRUE
E86	volume depletion	Finngen	AD	10.65	4.09	27.71	1.24E-06	3.88E-06	161.00		TRUE
	volume depletion	UKB		1.78	1.52	2.10	3.26E-12	8.57E-11	153	4,911	TRUE

AD: Alzheimer’s disease

UKB: UK Biobank

SAIL: Secure Anonymised Information Linkage Databank

Prior_ICD10_code: initial diagnosis of endocrine, metabolic, digestive system, and nutritional disorders

ci_min: Confidence interval minimum

ci_max: Confidence interval maximum

P_VAL: p-value

P_VAL_FDR_CORRECTED: p-value after False Discovery Rate corrected

N_pairs: Number of individuals identified with both ICD-10 code and neurodegenerative disease outcome

n: Number of individulas identified with ICD-10 code

**Table 2.** Replicated diagnoses in endocrine, nutritional, metabolic, and digestive systems associated with PD risk

Prior_ICD10_code	Prior_ICD10_code_Description	Dataset	Neurodegenerative disease outcome	Hazard Ratio	ci_min	ci_max	P_VAL	P_VAL_FDR_CORRECTED	N_pairs	n	rejected
K57	diverticular disease of intestine	Sail	PD	0.75	0.69	0.81	1.74E-11	2.82E-10	555.00	70,198.00	TRUE
	diverticular disease of intestine	UKB		0.69	0.62	0.77	6.24E-11	2.04E-09	354.00	34,801.00	TRUE
K30	functional dyspepsia	Finngen	PD	2.72	1.96	3.76	1.95E-09	1.21E-08	86.00		TRUE
	dyspepsia	UKB		1.34	1.13	1.60	8.51E-04	9.26E-03	135.00	8,811.00	TRUE
E10	insulin-dependent diabetes mellitus	Sail	PD	1.59	1.31	1.93	3.36E-06	2.48E-05	101.00	6,668.00	TRUE
	insulin-dependent diabetes mellitus	UKB		2.65	2.02	3.48	2.44E-12	1.20E-10	53.00	1,608.00	TRUE
E11	Type 2 diabetes, wide definition*(Includes E11)	Finngen	PD	2.04	1.75	2.39	2.06E-19	3.38E-18	421.00		TRUE
	non-insulin-dependent diabetes mellitus	UKB		1.21	1.08	1.36	1.36E-03	1.33E-02	330.00	18,815.00	TRUE
K63	other diseases of intestine	Sail	PD	0.84	0.73	0.97	1.51E-02	4.71E-02	205.00	25,877.00	TRUE
	other diseases of intestine	UKB		0.69	0.59	0.80	1.34E-06	2.20E-05	183.00	19,139.00	TRUE
K66	other disorders of peritoneum	Sail	PD	0.70	0.53	0.93	1.32E-02	4.21E-02	48.00	7,406.00	TRUE
	other disorders of peritoneum	UKB		0.63	0.45	0.87	5.81E-03	4.42E-02	35.00	4,612.00	TRUE
K59	other functional intestinal disorders	Finngen	PD	3.19	2.46	4.14	2.02E-18	3.02E-17	178.00		TRUE
	other functional intestinal disorders	UKB		1.56	1.38	1.76	4.81E-13	4.71E-11	301.00	15,431.00	TRUE

PD: Parkinson’s disease

UKB: UK Biobank

SAIL: Secure Anonymised Information Linkage Databank

Prior_ICD10_code: initial diagnosis of endocrine, metabolic, digestive system, and nutritional disorders

ci_min: Confidence interval minimum

ci_max: Confidence interval maximum

P_VAL: p-value

P_VAL_FDR_CORRECTED: p-value after False Discovery Rate corrected

N_pairs: Number of individuals identified with both ICD-10 code and neurodegenerative disease outcome

n: Number of Individulas Identified with ICD10_code

**Table 3.** Time-Stratified Cox Regression model for AD

ICD10_code	ICD10_code_description	ICD10_diagnosis_range	Neurodegenerative disease outcome	Hazard Ratio	ci_min	ci_max	P_VAL	N_pairs	n	P_VAL_FDR_CO	rejected
A04	Other Bacterial Intestinal Infections	1–5	AD	2.12	1.28	3.53	3.63E-03	15	434	6.60E-03	TRUE
A04	Other Bacterial Intestinal Infections	All	AD	1.51	1.21	1.89	2.35E-04	81	3585	3.35E-04	TRUE
E10	Insulin-Dependent Diabetes Mellitus	1–5	AD	3.13	1.85	5.29	2.05E-05	14	325	5.11E-05	TRUE
E10	Insulin-Dependent Diabetes Mellitus	5–10	AD	3.08	1.94	4.90	1.96E-06	18	398	9.78E-06	TRUE
E10	Insulin-Dependent Diabetes Mellitus	10–15	AD	3.68	2.32	5.85	3.55E-08	18	318	1.60E-07	TRUE
E10	Insulin-Dependent Diabetes Mellitus	All	AD	3.09	2.45	3.90	1.77E-21	73	1633	3.55E-20	TRUE
E11	Non-Insulin-Dependent Diabetes Mellitus	5–10	AD	1.32	1.11	1.58	1.91E-03	128	5496	4.78E-03	TRUE
E11	Non-Insulin-Dependent Diabetes Mellitus	10–15	AD	1.82	1.53	2.17	1.59E-11	133	4023	2.86E-10	TRUE
E11	Non-Insulin-Dependent Diabetes Mellitus	All	AD	1.47	1.33	1.63	1.19E-14	476	19048	7.96E-14	TRUE
E14	Unspecified Diabetes Mellitus	5–10	AD	1.79	1.13	2.85	1.36E-02	18	827	2.47E-02	TRUE
E14	Unspecified Diabetes Mellitus	10–15	AD	2.28	1.76	2.94	4.16E-10	59	1803	3.74E-09	TRUE
E14	Unspecified Diabetes Mellitus	All	AD	1.53	1.33	1.75	1.21E-09	224	8733	3.02E-09	TRUE
E16	Other Disorders Of Pancreatic Internal Secretion	1–5	AD	3.52	2.41	5.15	7.75E-11	27	374	1.55E-09	TRUE
E16	Other Disorders Of Pancreatic Internal Secretion	5–10	AD	1.87	1.21	2.91	5.12E-03	20	525	1.02E-02	TRUE
E16	Other Disorders Of Pancreatic Internal Secretion	10–15	AD	2.69	1.34	5.38	5.23E-03	8	180	1.18E-02	TRUE
E16	Other Disorders Of Pancreatic Internal Secretion	All	AD	2.23	1.75	2.83	5.27E-11	69	1637	1.76E-10	TRUE
E53	Deficiency Of Other B Group Vitamins	1–5	AD	2.33	1.66	3.27	9.65E-07	34	700	3.86E-06	TRUE
E53	Deficiency Of Other B Group Vitamins	5–10	AD	1.74	1.08	2.80	2.29E-02	17	509	3.81E-02	TRUE
E53	Deficiency Of Other B Group Vitamins	All	AD	1.78	1.40	2.27	2.61E-06	68	1939	4.75E-06	TRUE
E55	Vitamin D Deficiency	1–5	AD	2.35	1.80	3.06	2.47E-10	56	1198	2.47E-09	TRUE
E55	Vitamin D Deficiency	All	AD	1.95	1.57	2.41	9.97E-10	87	2405	2.85E-09	TRUE
E66	Obesity	All	AD	0.85	0.74	0.96	9.29E-03	260	22619	9.29E-03	TRUE
E78	Disorders Of Lipoprotein Metabolism And Other Lipidaemias	5–10	AD	1.48	1.29	1.70	3.33E-08	212	8983	3.33E-07	TRUE
E78	Disorders Of Lipoprotein Metabolism And Other Lipidaemias	10–15	AD	1.44	1.27	1.63	4.43E-09	288	12012	2.66E-08	TRUE
E78	Disorders Of Lipoprotein Metabolism And Other Lipidaemias	All	AD	1.18	1.09	1.27	1.53E-05	1090	53797	2.56E-05	TRUE
E83	Disorders Of Mineral Metabolism	1–5	AD	1.64	1.22	2.21	9.34E-04	45	1522	2.08E-03	TRUE
E83	Disorders Of Mineral Metabolism	All	AD	1.32	1.08	1.62	7.75E-03	94	4435	8.16E-03	TRUE
E85	Amyloidosis	1–5	AD	3.59	1.61	8.00	1.75E-03	6	81	3.50E-03	TRUE
E85	Amyloidosis	All	AD	2.57	1.34	4.94	4.72E-03	9	182	5.55E-03	TRUE
E86	Volume Depletion	1–5	AD	1.90	1.48	2.44	5.01E-07	63	1678	2.50E-06	TRUE
E86	Volume Depletion	5–10	AD	1.89	1.32	2.71	5.28E-04	30	795	1.56E-03	TRUE
E86	Volume Depletion	10–15	AD	2.09	1.28	3.41	3.30E-03	16	374	9.91E-03	TRUE
E86	Volume Depletion	All	AD	1.78	1.52	2.10	3.26E-12	153	4911	1.63E-11	TRUE
E87	Other Disorders Of Fluid, Electrolyte And Acid-Base Balance	1–5	AD	1.52	1.27	1.81	3.99E-06	128	4059	1.14E-05	TRUE
E87	Other Disorders Of Fluid, Electrolyte And Acid-Base Balance	5–10	AD	1.72	1.37	2.16	3.78E-06	75	1965	1.51E-05	TRUE
E87	Other Disorders Of Fluid, Electrolyte And Acid-Base Balance	10–15	AD	1.92	1.34	2.75	3.70E-04	30	786	1.33E-03	TRUE
E87	Other Disorders Of Fluid, Electrolyte And Acid-Base Balance	All	AD	1.51	1.34	1.70	6.43E-12	312	11313	2.57E-11	TRUE
K04	Diseases Of Pulp And Periapical Tissues	1–5	AD	2.41	1.20	4.82	1.31E-02	8	305	1.87E-02	TRUE
K04	Diseases Of Pulp And Periapical Tissues	All	AD	1.44	1.11	1.87	6.21E-03	57	3365	6.90E-03	TRUE
K20	Oesophagitis	1–5	AD	1.62	1.16	2.28	4.88E-03	34	1399	8.14E-03	TRUE
K20	Oesophagitis	5–10	AD	1.51	1.15	1.99	3.18E-03	52	2290	7.06E-03	TRUE
K20	Oesophagitis	All	AD	1.28	1.10	1.49	1.63E-03	175	8660	2.03E-03	TRUE
K29	Gastritis And Duodenitis	5–10	AD	1.32	1.13	1.55	5.47E-04	160	7742	1.56E-03	TRUE
K29	Gastritis And Duodenitis	10–15	AD	1.31	1.09	1.57	3.89E-03	120	5837	1.00E-02	TRUE
K29	Gastritis And Duodenitis	All	AD	1.20	1.09	1.32	1.45E-04	523	27914	2.23E-04	TRUE
K52	Other Non-Infective Gastro-Enteritis And Colitis	5–10	AD	2.31	1.82	2.93	6.20E-12	69	2086	1.24E-10	TRUE
K52	Other Non-Infective Gastro-Enteritis And Colitis	10–15	AD	1.36	1.08	1.70	7.92E-03	77	3697	1.43E-02	TRUE
K52	Other Non-Infective Gastro-Enteritis And Colitis	All	AD	1.37	1.20	1.56	1.75E-06	250	12984	3.50E-06	TRUE
K59	Other Functional Intestinal Disorders	1–5	AD	1.55	1.30	1.85	1.27E-06	127	4476	4.22E-06	TRUE
K59	Other Functional Intestinal Disorders	5–10	AD	1.70	1.40	2.07	8.75E-08	105	3464	5.83E-07	TRUE
K59	Other Functional Intestinal Disorders	10–15	AD	1.41	1.10	1.82	7.32E-03	61	2512	1.43E-02	TRUE
K59	Other Functional Intestinal Disorders	All	AD	1.55	1.40	1.73	3.55E-16	394	15596	3.55E-15	TRUE
K64	Haemorrhoids And Perianal Venous Thrombosis	1–5	AD	0.41	0.29	0.58	2.60E-07	34	5880	1.73E-06	TRUE
K64	Haemorrhoids And Perianal Venous Thrombosis	All	AD	0.69	0.59	0.80	6.94E-07	186	20162	1.54E-06	TRUE
K66	Other Disorders Of Peritoneum	1–5	AD	0.49	0.28	0.86	1.28E-02	12	1668	1.87E-02	TRUE
K66	Other Disorders Of Peritoneum	All	AD	0.59	0.44	0.81	8.77E-04	41	4636	1.17E-03	TRUE

AD: Alzheimer’s disease

ICD10_code: initial diagnosis of endocrine, metabolic, digestive system, and nutritional disorders

ICD10_diagnosis_range: Number of years from the initial diagnosis of endocrine, metabolic, digestive system, and nutritional disorders (ICD-10 code) to the occurrence of neurodegenerative disease outcome

ci_min: Confidence interval minimum

ci_max: Confidence interval maximum

P_VAL: p-value

N_pairs: Number of individuals identified with both ICD-10 code and neurodegenerative disease outcome

n: Number of Individulas Identified with ICD10_code

P_VAL_FDR_CORRECTED: p-value after False Discovery Rate corrected

**Table 4.** Time-Stratified Cox Regression model for PD

ICD10_code	ICD10_code_description	ICD10_diagnosis_range	Neurodegenerative disease outcome	Hazard Ratio	ci_min	ci_max	P_VAL	N_pairs	n	P_VAL_FDR_CO	rejected
E10	insulin-dependent diabetes mellitus	5–10	PD	3.08	1.89	5.04	7.15E-06	16	396	4.29E-05	TRUE
E10	insulin-dependent diabetes mellitus	10–15	PD	2.53	1.36	4.71	3.39E-03	10	310	1.02E-02	TRUE
E10	insulin-dependent diabetes mellitus	All	PD	2.65	2.02	3.48	2.44E-12	53	1608	1.46E-11	TRUE
E11	non-insulin-dependent diabetes mellitus	5–10	PD	1.41	1.16	1.70	4.19E-04	112	5480	8.38E-04	TRUE
E11	non-insulin-dependent diabetes mellitus	10–15	PD	1.40	1.13	1.74	2.03E-03	86	3976	8.10E-03	TRUE
E11	non-insulin-dependent diabetes mellitus	All	PD	1.21	1.08	1.36	1.36E-03	330	18815	1.63E-03	TRUE
E14	unspecified diabetes mellitus	1–5	PD	1.94	1.17	3.22	1.06E-02	15	623	1.58E-02	TRUE
E14	unspecified diabetes mellitus	5–10	PD	2.66	1.76	4.01	3.04E-06	23	832	3.65E-05	TRUE
E14	unspecified diabetes mellitus	10–15	PD	1.94	1.45	2.60	8.17E-06	46	1790	9.80E-05	TRUE
E14	unspecified diabetes mellitus	All	PD	1.61	1.39	1.86	1.81E-10	197	8658	5.44E-10	TRUE
E16	other disorders of pancreatic internal secretion	1–5	PD	1.89	1.04	3.42	3.54E-02	11	358	4.73E-02	TRUE
E16	other disorders of pancreatic internal secretion	5–10	PD	1.94	1.17	3.22	1.06E-02	15	520	1.81E-02	TRUE
E16	other disorders of pancreatic internal secretion	All	PD	1.84	1.36	2.47	6.76E-05	44	1602	1.16E-04	TRUE
E53	deficiency of other b group vitamins	1–5	PD	2.04	1.34	3.10	8.81E-04	22	688	2.64E-03	TRUE
E53	deficiency of other b group vitamins	All	PD	1.72	1.30	2.29	1.88E-04	48	1907	2.81E-04	TRUE
E66	obesity	1–5	PD	0.63	0.47	0.83	1.45E-03	47	6561	2.95E-03	TRUE
E66	obesity	All	PD	0.82	0.71	0.94	5.86E-03	211	22453	5.86E-03	TRUE
K30	dyspepsia	5–10	PD	1.84	1.39	2.44	1.90E-05	50	2480	7.61E-05	TRUE
K30	dyspepsia	All	PD	1.34	1.13	1.60	8.51E-04	135	8811	1.13E-03	TRUE
K57	diverticular disease of intestine	1–5	PD	0.60	0.48	0.75	8.54E-06	77	10132	5.12E-05	TRUE
K57	diverticular disease of intestine	5–10	PD	0.68	0.56	0.83	1.19E-04	104	10867	2.85E-04	TRUE
K57	diverticular disease of intestine	All	PD	0.69	0.62	0.77	6.24E-11	354	34801	2.50E-10	TRUE
K59	other functional intestinal disorders	1–5	PD	1.51	1.23	1.86	8.27E-05	94	4443	3.31E-04	TRUE
K59	other functional intestinal disorders	5–10	PD	1.62	1.29	2.03	3.70E-05	76	3435	1.11E-04	TRUE
K59	other functional intestinal disorders	10–15	PD	1.61	1.22	2.12	7.90E-04	51	2502	4.74E-03	TRUE
K59	other functional intestinal disorders	All	PD	1.56	1.38	1.76	4.81E-13	301	15431	5.77E-12	TRUE
K63	other diseases of intestine	1–5	PD	0.61	0.45	0.83	1.47E-03	42	5726	2.95E-03	TRUE
K63	other diseases of intestine	5–10	PD	0.74	0.57	0.95	1.67E-02	62	6182	2.23E-02	TRUE
K63	other diseases of intestine	10–15	PD	0.70	0.52	0.94	1.84E-02	44	4008	4.40E-02	TRUE
K63	other diseases of intestine	All	PD	0.69	0.59	0.80	1.34E-06	183	19139	2.69E-06	TRUE
K64	haemorrhoids and perianal venous thrombosis	1–5	PD	0.39	0.27	0.57	1.01E-06	27	5873	1.22E-05	TRUE
K64	haemorrhoids and perianal venous thrombosis	5–10	PD	0.66	0.47	0.92	1.55E-02	34	4640	2.23E-02	TRUE
K64	haemorrhoids and perianal venous thrombosis	All	PD	0.59	0.50	0.71	3.62E-09	136	20006	8.69E-09	TRUE
K66	other disorders of peritoneum	1–5	PD	0.34	0.16	0.72	4.79E-03	7	1663	8.21E-03	TRUE
K66	other disorders of peritoneum	All	PD	0.63	0.45	0.87	5.81E-03	35	4612	5.86E-03	TRUE

PD: Parkinson’s disease

ICD10_code: initial diagnosis of endocrine, metabolic, digestive system, and nutritional disorders

ICD10_diagnosis_range: Number of years from the initial diagnosis of endocrine, metabolic, digestive system, and nutritional disorders (ICD-10 code) to the occurrence of neurodegenerative disease outcome

ci_min: Confidence interval minimum

ci_max: Confidence interval maximum

P_VAL: p-value

N_pairs: Number of individuals identified with both ICD-10 code and neurodegenerative disease outcome

n: Number of Individulas Identified with ICD10_code

P_VAL_FDR_CORRECTED: p-value after False Discovery Rate corrected

**Table 5.** Proteomic biomarker comparison in isolated AD cases vs. cases with digestive, endocrine, metabolic, and nutritional conditions

Olink_marker_being_compared	Olink_marker_definition	ICD10_Code	ICD10_Code_definition	Average Olink_marker level in individuals diagnosed with only AD (AD)	Average Olink_marker level in individuals diagnosed with both AD and ICD-10 code (AD + ICD-10)	T-Statistic (AD + ICD-10 vs. AD)	Degrees of Freedom	AD_size	AD_ICD10_size	P-Value	P_VAL_FDR_CORRECTED	FDR_CORRECTED_rejected
gfap	Glial fibrillary acidic protein	p130708	Non-Insulin-Dependent Diabetes Mellitus	0.735	0.45	−4.05	544	437	109	8.20E-05	1.45E-03	TRUE
gfap	Glial fibrillary acidic protein	p131598	Gastritis And Duodenitis	0.722	0.50	−3.46	544	439	107	6.74E-04	8.42E-03	TRUE
gfap	Glial fibrillary acidic protein	p130714	Unspecified Diabetes Mellitus	0.713	0.42	−3.31	544	482	64	1.43E-03	1.47E-02	TRUE
gfap	Glial fibrillary acidic protein	p130770	Deficiency Of Other B Group Vitamins	0.694	0.46	−2.40	544	510	36	2.10E-02	9.83E-02	FALSE
gfap	Glial fibrillary acidic protein	p131654	Other Disorders Of Peritoneum	0.684	0.42	−2.33	544	534	12	3.72E-02	1.45E-01	FALSE
gfap	Glial fibrillary acidic protein	p130706	Insulin-Dependent Diabetes Mellitus	0.691	0.29	−2.15	544	529	17	4.61E-02	1.71E-01	FALSE
gfap	Glial fibrillary acidic protein	p130792	Obesity	0.700	0.49	−2.14	544	487	59	3.59E-02	1.44E-01	FALSE
gfap	Glial fibrillary acidic protein	p131640	Other Functional Intestinal Disorders	0.705	0.60	−1.71	544	410	136	8.91E-02	2.53E-01	FALSE
gfap	Glial fibrillary acidic protein	p130718	Other Disorders Of Pancreatic Internal Secretion	0.690	0.46	−1.69	544	518	28	1.02E-01	2.69E-01	FALSE
gfap	Glial fibrillary acidic protein	p130814	Disorders Of Lipoprotein Metabolism And Other Lipidaemias	0.716	0.64	−1.46	544	288	258	1.44E-01	3.38E-01	FALSE
gfap	Glial fibrillary acidic protein	p130820	Disorders Of Mineral Metabolism	0.685	0.58	−1.00	544	512	34	3.22E-01	5.54E-01	FALSE
gfap	Glial fibrillary acidic protein	p130828	Other Disorders Of Fluid, Electrolyte And Acid-Base Balance	0.693	0.63	−0.93	544	411	135	3.55E-01	5.81E-01	FALSE
gfap	Glial fibrillary acidic protein	p131650	Haemorrhoids And Perianal Venous Thrombosis	0.684	0.62	−0.67	544	493	53	5.06E-01	7.18E-01	FALSE
gfap	Glial fibrillary acidic protein	p131582	Oesophagitis	0.681	0.64	−0.36	544	508	38	7.20E-01	8.55E-01	FALSE
gfap	Glial fibrillary acidic protein	p131630	Other Non-Infective Gastro-Enteritis And Colitis	0.680	0.66	−0.27	544	491	55	7.91E-01	8.99E-01	FALSE
gfap	Glial fibrillary acidic protein	p131560	Diseases Of Pulp And Periapical Tissues	0.679	0.63	−0.25	544	538	8	8.07E-01	9.08E-01	FALSE
gfap	Glial fibrillary acidic protein	p130826	Volume Depletion	0.680	0.67	−0.14	544	434	112	8.88E-01	9.35E-01	FALSE
gfap	Glial fibrillary acidic protein	p130008	Other Bacterial Intestinal Infections	0.678	0.67	−0.07	544	523	23	9.47E-01	9.66E-01	FALSE
gfap	Glial fibrillary acidic protein	p130824	Amyloidosis	0.676	1.11	0.89	544	543	3	4.69E-01	6.89E-01	FALSE
gfap	Glial fibrillary acidic protein	p130774	Vitamin D Deficiency	0.671	0.79	1.16	544	512	34	2.54E-01	5.04E-01	FALSE
nefl	Neurofilament light polypeptide	p131654	Other Disorders Of Peritoneum	0.538	0.22	−2.92	536	526	12	1.28E-02	7.14E-02	FALSE
nefl	Neurofilament light polypeptide	p131598	Gastritis And Duodenitis	0.550	0.45	−1.74	536	435	103	8.46E-02	2.52E-01	FALSE
nefl	Neurofilament light polypeptide	p130770	Deficiency Of Other B Group Vitamins	0.539	0.43	−1.46	536	501	37	1.51E-01	3.52E-01	FALSE
nefl	Neurofilament light polypeptide	p131650	Haemorrhoids And Perianal Venous Thrombosis	0.538	0.46	−0.96	536	488	50	3.40E-01	5.64E-01	FALSE
nefl	Neurofilament light polypeptide	p130814	Disorders Of Lipoprotein Metabolism And Other Lipidaemias	0.545	0.52	−0.69	536	286	252	4.93E-01	7.07E-01	FALSE
nefl	Neurofilament light polypeptide	p131630	Other Non-Infective Gastro-Enteritis And Colitis	0.536	0.49	−0.64	536	484	54	5.27E-01	7.35E-01	FALSE
nefl	Neurofilament light polypeptide	p130792	Obesity	0.534	0.51	−0.37	536	479	59	7.14E-01	8.53E-01	FALSE
nefl	Neurofilament light polypeptide	p130820	Disorders Of Mineral Metabolism	0.534	0.49	−0.32	536	503	35	7.53E-01	8.78E-01	FALSE
nefl	Neurofilament light polypeptide	p131560	Diseases Of Pulp And Periapical Tissues	0.532	0.45	−0.30	536	531	7	7.77E-01	8.90E-01	FALSE
nefl	Neurofilament light polypeptide	p130708	Non-Insulin-Dependent Diabetes Mellitus	0.534	0.52	−0.28	536	430	108	7.81E-01	8.90E-01	FALSE
nefl	Neurofilament light polypeptide	p130008	Other Bacterial Intestinal Infections	0.532	0.50	−0.20	536	516	22	8.44E-01	9.11E-01	FALSE
nefl	Neurofilament light polypeptide	p130828	Other Disorders Of Fluid, Electrolyte And Acid-Base Balance	0.533	0.53	−0.14	536	407	131	8.91E-01	9.35E-01	FALSE
nefl	Neurofilament light polypeptide	p130706	Insulin-Dependent Diabetes Mellitus	0.531	0.53	0.00	536	520	18	9.99E-01	9.99E-01	FALSE
nefl	Neurofilament light polypeptide	p130826	Volume Depletion	0.530	0.54	0.10	536	431	107	9.20E-01	9.52E-01	FALSE
nefl	Neurofilament light polypeptide	p131582	Oesophagitis	0.528	0.56	0.39	536	499	39	6.99E-01	8.50E-01	FALSE
nefl	Neurofilament light polypeptide	p130714	Unspecified Diabetes Mellitus	0.527	0.56	0.45	536	474	64	6.55E-01	8.38E-01	FALSE
nefl	Neurofilament light polypeptide	p130824	Amyloidosis	0.530	0.66	0.91	536	535	3	4.56E-01	6.84E-01	FALSE
nefl	Neurofilament light polypeptide	p130774	Vitamin D Deficiency	0.522	0.66	1.43	536	505	33	1.61E-01	3.60E-01	FALSE
nefl	Neurofilament light polypeptide	p130718	Other Disorders Of Pancreatic Internal Secretion	0.520	0.73	1.78	536	510	28	8.50E-02	2.52E-01	FALSE
nefl	Neurofilament light polypeptide	p131640	Other Functional Intestinal Disorders	0.507	0.60	1.88	536	402	136	6.07E-02	2.02E-01	FALSE
adgrg1	Adhesion G-protein coupled receptor G	p130824	Amyloidosis	0.584	0.27	−1.29	556	556	2	4.09E-01	6.50E-01	FALSE
adgrg1	Adhesion G-protein coupled receptor G	p130008	Other Bacterial Intestinal Infections	0.585	0.54	−0.21	556	533	25	8.37E-01	9.08E-01	FALSE
adgrg1	Adhesion G-protein coupled receptor G	p131640	Other Functional Intestinal Disorders	0.572	0.62	0.40	556	418	140	6.90E-01	8.50E-01	FALSE
adgrg1	Adhesion G-protein coupled receptor G	p131560	Diseases Of Pulp And Periapical Tissues	0.581	0.72	0.48	556	551	7	6.46E-01	8.35E-01	FALSE
adgrg1	Adhesion G-protein coupled receptor G	p130774	Vitamin D Deficiency	0.571	0.78	0.90	556	524	34	3.77E-01	6.08E-01	FALSE
adgrg1	Adhesion G-protein coupled receptor G	p131654	Other Disorders Of Peritoneum	0.576	0.92	1.02	556	547	11	3.29E-01	5.54E-01	FALSE
adgrg1	Adhesion G-protein coupled receptor G	p131582	Oesophagitis	0.568	0.79	1.09	556	520	38	2.82E-01	5.23E-01	FALSE
adgrg1	Adhesion G-protein coupled receptor G	p131650	Haemorrhoids And Perianal Venous Thrombosis	0.564	0.76	1.19	556	504	54	2.38E-01	4.80E-01	FALSE
adgrg1	Adhesion G-protein coupled receptor G	p130770	Deficiency Of Other B Group Vitamins	0.565	0.85	1.27	556	521	37	2.11E-01	4.33E-01	FALSE
adgrg1	Adhesion G-protein coupled receptor G	p130820	Disorders Of Mineral Metabolism	0.561	0.92	1.74	556	524	34	9.10E-02	2.54E-01	FALSE
adgrg1	Adhesion G-protein coupled receptor G	p131598	Gastritis And Duodenitis	0.541	0.76	1.76	556	451	107	8.12E-02	2.48E-01	FALSE
adgrg1	Adhesion G-protein coupled receptor G	p130814	Disorders Of Lipoprotein Metabolism And Other Lipidaemias	0.500	0.68	1.97	556	296	262	4.93E-02	1.74E-01	FALSE
adgrg1	Adhesion G-protein coupled receptor G	p131630	Other Non-Infective Gastro-Enteritis And Colitis	0.546	0.93	2.00	556	504	54	5.03E-02	1.74E-01	FALSE
adgrg1	Adhesion G-protein coupled receptor G	p130828	Other Disorders Of Fluid, Electrolyte And Acid-Base Balance	0.526	0.76	2.17	556	419	139	3.07E-02	1.32E-01	FALSE
adgrg1	Adhesion G-protein coupled receptor G	p130826	Volume Depletion	0.526	0.80	2.36	556	443	115	1.94E-02	9.54E-02	FALSE
adgrg1	Adhesion G-protein coupled receptor G	p130718	Other Disorders Of Pancreatic Internal Secretion	0.548	1.18	2.62	556	527	31	1.32E-02	7.14E-02	FALSE
adgrg1	Adhesion G-protein coupled receptor G	p130706	Insulin-Dependent Diabetes Mellitus	0.551	1.60	2.97	556	541	17	8.81E-03	5.74E-02	FALSE
adgrg1	Adhesion G-protein coupled receptor G	p130792	Obesity	0.485	1.39	4.80	556	497	61	9.17E-06	2.29E-04	TRUE
adgrg1	Adhesion G-protein coupled receptor G	p130714	Unspecified Diabetes Mellitus	0.455	1.52	6.12	556	491	67	4.08E-08	1.75E-06	TRUE
adgrg1	Adhesion G-protein coupled receptor G	p130708	Non-Insulin-Dependent Diabetes Mellitus	0.393	1.35	7.21	556	447	111	3.60E-11	5.40E-09	TRUE
calb1	Calbindin	p131560	Diseases Of Pulp And Periapical Tissues	0.183	−0.16	−3.02	556	551	7	2.16E-02	9.99E-02	FALSE
calb1	Calbindin	p131654	Other Disorders Of Peritoneum	0.181	0.07	−1.20	556	547	11	2.57E-01	5.04E-01	FALSE
calb1	Calbindin	p130706	Insulin-Dependent Diabetes Mellitus	0.180	0.15	−0.17	556	541	17	8.68E-01	9.26E-01	FALSE
calb1	Calbindin	p130826	Volume Depletion	0.180	0.17	−0.15	556	443	115	8.83E-01	9.33E-01	FALSE
calb1	Calbindin	p130792	Obesity	0.179	0.18	0.05	556	497	61	9.64E-01	9.74E-01	FALSE
calb1	Calbindin	p131640	Other Functional Intestinal Disorders	0.176	0.19	0.21	556	418	140	8.30E-01	9.08E-01	FALSE
calb1	Calbindin	p131630	Other Non-Infective Gastro-Enteritis And Colitis	0.177	0.20	0.29	556	504	54	7.74E-01	8.90E-01	FALSE
calb1	Calbindin	p130770	Deficiency Of Other B Group Vitamins	0.176	0.22	0.59	556	521	37	5.62E-01	7.62E-01	FALSE
calb1	Calbindin	p130814	Disorders Of Lipoprotein Metabolism And Other Lipidaemias	0.168	0.19	0.59	556	296	262	5.55E-01	7.57E-01	FALSE
calb1	Calbindin	p130828	Other Disorders Of Fluid, Electrolyte And Acid-Base Balance	0.171	0.20	0.63	556	419	139	5.30E-01	7.35E-01	FALSE
calb1	Calbindin	p130708	Non-Insulin-Dependent Diabetes Mellitus	0.169	0.22	0.92	556	447	111	3.59E-01	5.82E-01	FALSE
calb1	Calbindin	p130008	Other Bacterial Intestinal Infections	0.173	0.30	1.31	556	533	25	2.03E-01	4.30E-01	FALSE
calb1	Calbindin	p131650	Haemorrhoids And Perianal Venous Thrombosis	0.169	0.27	1.50	556	504	54	1.38E-01	3.30E-01	FALSE
calb1	Calbindin	p131598	Gastritis And Duodenitis	0.164	0.24	1.59	556	451	107	1.14E-01	2.90E-01	FALSE
calb1	Calbindin	p130714	Unspecified Diabetes Mellitus	0.165	0.28	1.69	556	491	67	9.51E-02	2.62E-01	FALSE
calb1	Calbindin	p130820	Disorders Of Mineral Metabolism	0.167	0.36	2.03	556	524	34	5.04E-02	1.74E-01	FALSE
calb1	Calbindin	p130774	Vitamin D Deficiency	0.167	0.36	2.43	556	524	34	2.01E-02	9.59E-02	FALSE
calb1	Calbindin	p131582	Oesophagitis	0.162	0.41	2.96	556	520	38	5.05E-03	4.10E-02	TRUE
calb1	Calbindin	p130718	Other Disorders Of Pancreatic Internal Secretion	0.167	0.38	3.07	556	527	31	4.06E-03	3.48E-02	TRUE
calb1	Calbindin	p130824	Amyloidosis	0.177	0.55	12.23	556	556	2	1.86E-03	1.80E-02	TRUE
dcn	Decorin	p130824	Amyloidosis	0.138	0.05	−0.80	552	551	3	5.07E-01	7.18E-01	FALSE
dcn	Decorin	p131630	Other Non-Infective Gastro-Enteritis And Colitis	0.139	0.12	−0.45	552	500	54	6.56E-01	8.38E-01	FALSE
dcn	Decorin	p131560	Diseases Of Pulp And Periapical Tissues	0.137	0.12	−0.22	552	546	8	8.30E-01	9.08E-01	FALSE
dcn	Decorin	p131598	Gastritis And Duodenitis	0.135	0.14	0.35	552	446	108	7.23E-01	8.55E-01	FALSE
dcn	Decorin	p130008	Other Bacterial Intestinal Infections	0.136	0.16	0.50	552	528	26	6.22E-01	8.08E-01	FALSE
dcn	Decorin	p131640	Other Functional Intestinal Disorders	0.132	0.15	0.80	552	415	139	4.23E-01	6.54E-01	FALSE
dcn	Decorin	p130770	Deficiency Of Other B Group Vitamins	0.134	0.18	0.97	552	516	38	3.37E-01	5.62E-01	FALSE
dcn	Decorin	p130814	Disorders Of Lipoprotein Metabolism And Other Lipidaemias	0.126	0.15	1.08	552	295	259	2.81E-01	5.23E-01	FALSE
dcn	Decorin	p131650	Haemorrhoids And Perianal Venous Thrombosis	0.133	0.18	1.09	552	500	54	2.81E-01	5.23E-01	FALSE
dcn	Decorin	p130708	Non-Insulin-Dependent Diabetes Mellitus	0.130	0.16	1.14	552	443	111	2.57E-01	5.04E-01	FALSE
dcn	Decorin	p131654	Other Disorders Of Peritoneum	0.135	0.23	1.34	552	542	12	2.06E-01	4.30E-01	FALSE
dcn	Decorin	p130706	Insulin-Dependent Diabetes Mellitus	0.133	0.27	1.59	552	537	17	1.31E-01	3.20E-01	FALSE
dcn	Decorin	p131582	Oesophagitis	0.132	0.21	1.59	552	515	39	1.18E-01	2.95E-01	FALSE
dcn	Decorin	p130774	Vitamin D Deficiency	0.132	0.22	1.67	552	521	33	1.03E-01	2.69E-01	FALSE
dcn	Decorin	p130714	Unspecified Diabetes Mellitus	0.129	0.20	1.78	552	488	66	7.90E-02	2.47E-01	FALSE
dcn	Decorin	p130718	Other Disorders Of Pancreatic Internal Secretion	0.132	0.24	1.80	552	526	28	8.22E-02	2.49E-01	FALSE
dcn	Decorin	p130820	Disorders Of Mineral Metabolism	0.131	0.23	2.17	552	520	34	3.65E-02	1.44E-01	FALSE
dcn	Decorin	p130792	Obesity	0.124	0.24	2.62	552	495	59	1.08E-02	6.74E-02	FALSE
dcn	Decorin	p130828	Other Disorders Of Fluid, Electrolyte And Acid-Base Balance	0.118	0.20	2.86	552	418	136	4.68E-03	3.90E-02	TRUE
dcn	Decorin	p130826	Volume Depletion	0.119	0.21	3.05	552	442	112	2.71E-03	2.54E-02	TRUE
psg1	Pregnancy-specific beta-1-glycoprotein	p130824	Amyloidosis	0.347	−0.53	−3.03	550	549	3	8.65E-02	2.53E-01	FALSE
psg1	Pregnancy-specific beta-1-glycoprotein	p130770	Deficiency Of Other B Group Vitamins	0.379	−0.17	−1.93	550	515	37	6.12E-02	2.02E-01	FALSE
psg1	Pregnancy-specific beta-1-glycoprotein	p131654	Other Disorders Of Peritoneum	0.348	0.07	−0.96	550	540	12	3.56E-01	5.81E-01	FALSE
psg1	Pregnancy-specific beta-1-glycoprotein	p130008	Other Bacterial Intestinal Infections	0.349	0.19	−0.52	550	527	25	6.10E-01	8.01E-01	FALSE
psg1	Pregnancy-specific beta-1-glycoprotein	p131650	Haemorrhoids And Perianal Venous Thrombosis	0.349	0.28	−0.40	550	496	56	6.89E-01	8.50E-01	FALSE
psg1	Pregnancy-specific beta-1-glycoprotein	p131630	Other Non-Infective Gastro-Enteritis And Colitis	0.350	0.27	−0.39	550	496	56	6.95E-01	8.50E-01	FALSE
psg1	Pregnancy-specific beta-1-glycoprotein	p130814	Disorders Of Lipoprotein Metabolism And Other Lipidaemias	0.358	0.33	−0.30	550	294	258	7.62E-01	8.86E-01	FALSE
psg1	Pregnancy-specific beta-1-glycoprotein	p131640	Other Functional Intestinal Disorders	0.340	0.35	0.06	550	415	137	9.52E-01	9.66E-01	FALSE
psg1	Pregnancy-specific beta-1-glycoprotein	p130792	Obesity	0.335	0.40	0.38	550	491	61	7.07E-01	8.51E-01	FALSE
psg1	Pregnancy-specific beta-1-glycoprotein	p131560	Diseases Of Pulp And Periapical Tissues	0.340	0.50	0.42	550	544	8	6.86E-01	8.50E-01	FALSE
psg1	Pregnancy-specific beta-1-glycoprotein	p131598	Gastritis And Duodenitis	0.328	0.40	0.52	550	445	107	6.05E-01	8.00E-01	FALSE
psg1	Pregnancy-specific beta-1-glycoprotein	p131582	Oesophagitis	0.335	0.45	0.62	550	516	36	5.37E-01	7.39E-01	FALSE
psg1	Pregnancy-specific beta-1-glycoprotein	p130826	Volume Depletion	0.323	0.42	0.73	550	438	114	4.63E-01	6.89E-01	FALSE
psg1	Pregnancy-specific beta-1-glycoprotein	p130820	Disorders Of Mineral Metabolism	0.329	0.56	1.01	550	519	33	3.21E-01	5.54E-01	FALSE
psg1	Pregnancy-specific beta-1-glycoprotein	p130718	Other Disorders Of Pancreatic Internal Secretion	0.323	0.70	1.29	550	523	29	2.06E-01	4.30E-01	FALSE
psg1	Pregnancy-specific beta-1-glycoprotein	p130708	Non-Insulin-Dependent Diabetes Mellitus	0.297	0.52	1.65	550	440	112	1.00E-01	2.69E-01	FALSE
psg1	Pregnancy-specific beta-1-glycoprotein	p130714	Unspecified Diabetes Mellitus	0.308	0.60	1.68	550	486	66	9.67E-02	2.64E-01	FALSE
psg1	Pregnancy-specific beta-1-glycoprotein	p130774	Vitamin D Deficiency	0.318	0.71	1.79	550	518	34	8.10E-02	2.48E-01	FALSE
psg1	Pregnancy-specific beta-1-glycoprotein	p130706	Insulin-Dependent Diabetes Mellitus	0.321	0.98	1.90	550	534	18	7.40E-02	2.34E-01	FALSE
psg1	Pregnancy-specific beta-1-glycoprotein	p130828	Other Disorders Of Fluid, Electrolyte And Acid-Base Balance	0.280	0.53	1.91	550	416	136	5.79E-02	1.95E-01	FALSE
pvr	Poliovirus receptor	p130008	Other Bacterial Intestinal Infections	0.120	0.02	−1.07	562	538	26	2.95E-01	5.33E-01	FALSE
pvr	Poliovirus receptor	p130820	Disorders Of Mineral Metabolism	0.116	0.10	−0.23	562	529	35	8.21E-01	9.08E-01	FALSE
pvr	Poliovirus receptor	p130718	Other Disorders Of Pancreatic Internal Secretion	0.116	0.10	−0.22	562	534	30	8.24E-01	9.08E-01	FALSE
pvr	Poliovirus receptor	p130770	Deficiency Of Other B Group Vitamins	0.116	0.10	−0.19	562	527	37	8.52E-01	9.16E-01	FALSE
pvr	Poliovirus receptor	p131650	Haemorrhoids And Perianal Venous Thrombosis	0.116	0.11	−0.16	562	508	56	8.71E-01	9.26E-01	FALSE
pvr	Poliovirus receptor	p130792	Obesity	0.116	0.11	−0.15	562	502	62	8.82E-01	9.33E-01	FALSE
pvr	Poliovirus receptor	p131640	Other Functional Intestinal Disorders	0.115	0.12	0.01	562	423	141	9.96E-01	9.99E-01	FALSE
pvr	Poliovirus receptor	p130828	Other Disorders Of Fluid, Electrolyte And Acid-Base Balance	0.111	0.13	0.39	562	427	137	7.00E-01	8.50E-01	FALSE
pvr	Poliovirus receptor	p130824	Amyloidosis	0.114	0.18	0.43	562	561	3	7.09E-01	8.51E-01	FALSE
pvr	Poliovirus receptor	p131630	Other Non-Infective Gastro-Enteritis And Colitis	0.111	0.15	0.63	562	508	56	5.31E-01	7.35E-01	FALSE
pvr	Poliovirus receptor	p131654	Other Disorders Of Peritoneum	0.113	0.20	0.79	562	552	12	4.44E-01	6.70E-01	FALSE
pvr	Poliovirus receptor	p130826	Volume Depletion	0.107	0.15	0.81	562	448	116	4.20E-01	6.54E-01	FALSE
pvr	Poliovirus receptor	p131598	Gastritis And Duodenitis	0.108	0.14	0.85	562	455	109	3.98E-01	6.35E-01	FALSE
pvr	Poliovirus receptor	p130814	Disorders Of Lipoprotein Metabolism And Other Lipidaemias	0.099	0.13	1.00	562	299	265	3.20E-01	5.54E-01	FALSE
pvr	Poliovirus receptor	p130706	Insulin-Dependent Diabetes Mellitus	0.111	0.22	1.00	562	546	18	3.30E-01	5.54E-01	FALSE
pvr	Poliovirus receptor	p130774	Vitamin D Deficiency	0.110	0.19	1.04	562	530	34	3.05E-01	5.44E-01	FALSE
pvr	Poliovirus receptor	p130714	Unspecified Diabetes Mellitus	0.103	0.20	1.72	562	496	68	8.95E-02	2.53E-01	FALSE
pvr	Poliovirus receptor	p131582	Oesophagitis	0.100	0.32	2.21	562	526	38	3.28E-02	1.37E-01	FALSE
pvr	Poliovirus receptor	p130708	Non-Insulin-Dependent Diabetes Mellitus	0.091	0.21	2.71	562	451	113	7.42E-03	5.22E-02	FALSE
pvr	Poliovirus receptor	p131560	Diseases Of Pulp And Periapical Tissues	0.111	0.38	3.20	562	556	8	1.34E-02	7.14E-02	FALSE
ren	Renin	p131560	Diseases Of Pulp And Periapical Tissues	0.411	0.24	−1.05	559	553	8	3.25E-01	5.54E-01	FALSE
ren	Renin	p131654	Other Disorders Of Peritoneum	0.411	0.28	−0.42	559	550	11	6.81E-01	8.50E-01	FALSE
ren	Renin	p130824	Amyloidosis	0.409	0.35	−0.14	559	558	3	9.03E-01	9.41E-01	FALSE
ren	Renin	p131650	Haemorrhoids And Perianal Venous Thrombosis	0.397	0.52	0.81	559	505	56	4.23E-01	6.54E-01	FALSE
ren	Renin	p131598	Gastritis And Duodenitis	0.387	0.50	0.98	559	452	109	3.30E-01	5.54E-01	FALSE
ren	Renin	p130008	Other Bacterial Intestinal Infections	0.395	0.68	1.09	559	534	27	2.86E-01	5.26E-01	FALSE
ren	Renin	p131630	Other Non-Infective Gastro-Enteritis And Colitis	0.385	0.61	1.44	559	503	58	1.53E-01	3.53E-01	FALSE
ren	Renin	p130820	Disorders Of Mineral Metabolism	0.390	0.70	1.51	559	526	35	1.39E-01	3.30E-01	FALSE
ren	Renin	p131582	Oesophagitis	0.389	0.67	1.62	559	521	40	1.13E-01	2.89E-01	FALSE
ren	Renin	p130774	Vitamin D Deficiency	0.379	0.85	2.20	559	526	35	3.43E-02	1.39E-01	FALSE
ren	Renin	p130828	Other Disorders Of Fluid, Electrolyte And Acid-Base Balance	0.347	0.60	2.25	559	424	137	2.57E-02	1.15E-01	FALSE
ren	Renin	p130826	Volume Depletion	0.340	0.67	2.77	559	445	116	6.23E-03	4.67E-02	TRUE
ren	Renin	p130770	Deficiency Of Other B Group Vitamins	0.367	1.01	3.41	559	524	37	1.46E-03	1.47E-02	TRUE
ren	Renin	p130792	Obesity	0.349	0.89	3.71	559	499	62	3.89E-04	5.07E-03	TRUE
ren	Renin	p131640	Other Functional Intestinal Disorders	0.310	0.70	3.76	559	420	141	2.17E-04	3.25E-03	TRUE
ren	Renin	p130706	Insulin-Dependent Diabetes Mellitus	0.369	1.62	3.78	559	543	18	1.43E-03	1.47E-02	TRUE
ren	Renin	p130718	Other Disorders Of Pancreatic Internal Secretion	0.349	1.46	4.30	559	531	30	1.60E-04	2.67E-03	TRUE
ren	Renin	p130714	Unspecified Diabetes Mellitus	0.293	1.25	5.73	559	493	68	1.86E-07	6.97E-06	TRUE
ren	Renin	p130708	Non-Insulin-Dependent Diabetes Mellitus	0.253	1.02	5.97	559	447	114	1.74E-08	8.71E-07	TRUE
ren	Renin	p130814	Disorders Of Lipoprotein Metabolism And Other Lipidaemias	0.161	0.69	6.02	559	296	265	3.38E-09	2.54E-07	TRUE
gdf15	Growth/differentiation factor 15	p130824	Amyloidosis	0.445	0.22	−1.90	568	567	3	1.88E-01	4.08E-01	FALSE
gdf15	Growth/differentiation factor 15	p131560	Diseases Of Pulp And Periapical Tissues	0.443	0.48	0.25	568	562	8	8.12E-01	9.08E-01	FALSE
gdf15	Growth/differentiation factor 15	p130008	Other Bacterial Intestinal Infections	0.438	0.55	0.57	568	543	27	5.74E-01	7.72E-01	FALSE
gdf15	Growth/differentiation factor 15	p131654	Other Disorders Of Peritoneum	0.441	0.56	0.58	568	558	12	5.73E-01	7.72E-01	FALSE
gdf15	Growth/differentiation factor 15	p131630	Other Non-Infective Gastro-Enteritis And Colitis	0.432	0.54	1.09	568	512	58	2.81E-01	5.23E-01	FALSE
gdf15	Growth/differentiation factor 15	p131598	Gastritis And Duodenitis	0.426	0.51	1.42	568	459	111	1.57E-01	3.56E-01	FALSE
gdf15	Growth/differentiation factor 15	p131650	Haemorrhoids And Perianal Venous Thrombosis	0.433	0.54	1.43	568	514	56	1.59E-01	3.58E-01	FALSE
gdf15	Growth/differentiation factor 15	p130774	Vitamin D Deficiency	0.430	0.64	2.05	568	535	35	4.77E-02	1.72E-01	FALSE
gdf15	Growth/differentiation factor 15	p130770	Deficiency Of Other B Group Vitamins	0.427	0.67	2.07	568	532	38	4.44E-02	1.69E-01	FALSE
gdf15	Growth/differentiation factor 15	p131640	Other Functional Intestinal Disorders	0.407	0.55	2.66	568	427	143	8.23E-03	5.49E-02	FALSE
gdf15	Growth/differentiation factor 15	p131582	Oesophagitis	0.415	0.82	3.12	568	530	40	3.32E-03	3.01E-02	TRUE
gdf15	Growth/differentiation factor 15	p130826	Volume Depletion	0.399	0.62	3.28	568	453	117	1.26E-03	1.45E-02	TRUE
gdf15	Growth/differentiation factor 15	p130820	Disorders Of Mineral Metabolism	0.418	0.83	3.89	568	535	35	3.85E-04	5.07E-03	TRUE
gdf15	Growth/differentiation factor 15	p130828	Other Disorders Of Fluid, Electrolyte And Acid-Base Balance	0.382	0.63	4.26	568	431	139	3.03E-05	6.49E-04	TRUE
gdf15	Growth/differentiation factor 15	p130706	Insulin-Dependent Diabetes Mellitus	0.418	1.22	4.47	568	552	18	3.12E-04	4.46E-03	TRUE
gdf15	Growth/differentiation factor 15	p130792	Obesity	0.400	0.80	4.48	568	508	62	2.76E-05	6.36E-04	TRUE
gdf15	Growth/differentiation factor 15	p130814	Disorders Of Lipoprotein Metabolism And Other Lipidaemias	0.338	0.56	4.68	568	302	268	3.64E-06	1.21E-04	TRUE
gdf15	Growth/differentiation factor 15	p130718	Other Disorders Of Pancreatic Internal Secretion	0.409	1.06	4.82	568	540	30	3.68E-05	7.36E-04	TRUE
gdf15	Growth/differentiation factor 15	p130708	Non-Insulin-Dependent Diabetes Mellitus	0.336	0.87	7.47	568	455	115	7.97E-12	2.39E-09	TRUE
gdf15	Growth/differentiation factor 15	p130714	Unspecified Diabetes Mellitus	0.359	1.06	7.51	568	501	69	9.20E-11	9.20E-09	TRUE
igf2r	Cation-independent mannose-6-phosp	p130008	Other Bacterial Intestinal Infections	0.081	0.02	−0.99	558	534	26	3.29E-01	5.54E-01	FALSE
igf2r	Cation-independent mannose-6-phosp	p130774	Vitamin D Deficiency	0.078	0.08	−0.06	558	526	34	9.54E-01	9.66E-01	FALSE
igf2r	Cation-independent mannose-6-phosp	p130824	Amyloidosis	0.078	0.13	0.25	558	557	3	8.26E-01	9.08E-01	FALSE
igf2r	Cation-independent mannose-6-phosp	p130826	Volume Depletion	0.076	0.09	0.36	558	445	115	7.21E-01	8.55E-01	FALSE
igf2r	Cation-independent mannose-6-phosp	p130770	Deficiency Of Other B Group Vitamins	0.076	0.10	0.51	558	523	37	6.14E-01	8.01E-01	FALSE
igf2r	Cation-independent mannose-6-phosp	p131650	Haemorrhoids And Perianal Venous Thrombosis	0.075	0.10	0.69	558	504	56	4.95E-01	7.08E-01	FALSE
igf2r	Cation-independent mannose-6-phosp	p131640	Other Functional Intestinal Disorders	0.074	0.09	0.69	558	419	141	4.88E-01	7.07E-01	FALSE
igf2r	Cation-independent mannose-6-phosp	p131654	Other Disorders Of Peritoneum	0.075	0.20	1.17	558	548	12	2.68E-01	5.14E-01	FALSE
igf2r	Cation-independent mannose-6-phosp	p131560	Diseases Of Pulp And Periapical Tissues	0.076	0.21	1.51	558	552	8	1.72E-01	3.80E-01	FALSE
igf2r	Cation-independent mannose-6-phosp	p131598	Gastritis And Duodenitis	0.069	0.12	1.63	558	452	108	1.05E-01	2.73E-01	FALSE
igf2r	Cation-independent mannose-6-phosp	p131630	Other Non-Infective Gastro-Enteritis And Colitis	0.072	0.13	1.84	558	504	56	7.00E-02	2.26E-01	FALSE
igf2r	Cation-independent mannose-6-phosp	p130820	Disorders Of Mineral Metabolism	0.073	0.16	1.85	558	525	35	7.20E-02	2.30E-01	FALSE
igf2r	Cation-independent mannose-6-phosp	p130828	Other Disorders Of Fluid, Electrolyte And Acid-Base Balance	0.066	0.12	1.98	558	423	137	4.90E-02	1.74E-01	FALSE
igf2r	Cation-independent mannose-6-phosp	p131582	Oesophagitis	0.072	0.16	2.04	558	521	39	4.77E-02	1.72E-01	FALSE
igf2r	Cation-independent mannose-6-phosp	p130814	Disorders Of Lipoprotein Metabolism And Other Lipidaemias	0.053	0.11	2.40	558	294	266	1.69E-02	8.43E-02	FALSE
igf2r	Cation-independent mannose-6-phosp	p130718	Other Disorders Of Pancreatic Internal Secretion	0.071	0.20	2.45	558	530	30	1.99E-02	9.59E-02	FALSE
igf2r	Cation-independent mannose-6-phosp	p130706	Insulin-Dependent Diabetes Mellitus	0.072	0.26	2.91	558	542	18	9.27E-03	5.92E-02	FALSE
igf2r	Cation-independent mannose-6-phosp	p130714	Unspecified Diabetes Mellitus	0.065	0.17	3.39	558	492	68	1.04E-03	1.24E-02	TRUE
igf2r	Cation-independent mannose-6-phosp	p130792	Obesity	0.064	0.19	3.94	558	498	62	1.74E-04	2.74E-03	TRUE
igf2r	Cation-independent mannose-6-phosp	p130708	Non-Insulin-Dependent Diabetes Mellitus	0.053	0.17	4.75	558	446	114	4.15E-06	1.24E-04	TRUE
il1r1	Interleukin-1 receptor type 1	p130824	Amyloidosis	0.060	−0.10	−0.84	556	555	3	4.90E-01	7.07E-01	FALSE
il1r1	Interleukin-1 receptor type 1	p130814	Disorders Of Lipoprotein Metabolism And Other Lipidaemias	0.063	0.05	−0.41	556	293	265	6.82E-01	8.50E-01	FALSE
il1r1	Interleukin-1 receptor type 1	p130008	Other Bacterial Intestinal Infections	0.060	0.05	−0.21	556	532	26	8.32E-01	9.08E-01	FALSE
il1r1	Interleukin-1 receptor type 1	p131560	Diseases Of Pulp And Periapical Tissues	0.059	0.05	−0.11	556	550	8	9.19E-01	9.52E-01	FALSE
il1r1	Interleukin-1 receptor type 1	p130770	Deficiency Of Other B Group Vitamins	0.058	0.07	0.28	556	522	36	7.79E-01	8.90E-01	FALSE
il1r1	Interleukin-1 receptor type 1	p131640	Other Functional Intestinal Disorders	0.057	0.07	0.34	556	418	140	7.31E-01	8.60E-01	FALSE
il1r1	Interleukin-1 receptor type 1	p131650	Haemorrhoids And Perianal Venous Thrombosis	0.057	0.08	0.63	556	503	55	5.30E-01	7.35E-01	FALSE
il1r1	Interleukin-1 receptor type 1	p130826	Volume Depletion	0.055	0.07	0.64	556	443	115	5.24E-01	7.35E-01	FALSE
il1r1	Interleukin-1 receptor type 1	p131630	Other Non-Infective Gastro-Enteritis And Colitis	0.056	0.08	0.71	556	503	55	4.83E-01	7.03E-01	FALSE
il1r1	Interleukin-1 receptor type 1	p131654	Other Disorders Of Peritoneum	0.058	0.13	1.19	556	546	12	2.59E-01	5.04E-01	FALSE
il1r1	Interleukin-1 receptor type 1	p131598	Gastritis And Duodenitis	0.052	0.09	1.26	556	451	107	2.08E-01	4.31E-01	FALSE
il1r1	Interleukin-1 receptor type 1	p130792	Obesity	0.054	0.11	1.31	556	496	62	1.95E-01	4.22E-01	FALSE
il1r1	Interleukin-1 receptor type 1	p130774	Vitamin D Deficiency	0.055	0.13	1.59	556	524	34	1.21E-01	2.99E-01	FALSE
il1r1	Interleukin-1 receptor type 1	p130820	Disorders Of Mineral Metabolism	0.054	0.14	1.87	556	523	35	6.89E-02	2.25E-01	FALSE
il1r1	Interleukin-1 receptor type 1	p131582	Oesophagitis	0.052	0.16	2.28	556	520	38	2.77E-02	1.22E-01	FALSE
il1r1	Interleukin-1 receptor type 1	p130828	Other Disorders Of Fluid, Electrolyte And Acid-Base Balance	0.044	0.11	2.49	556	422	136	1.36E-02	7.14E-02	FALSE
il1r1	Interleukin-1 receptor type 1	p130718	Other Disorders Of Pancreatic Internal Secretion	0.051	0.20	2.67	556	528	30	1.19E-02	7.14E-02	FALSE
il1r1	Interleukin-1 receptor type 1	p130706	Insulin-Dependent Diabetes Mellitus	0.051	0.30	3.75	556	540	18	1.47E-03	1.47E-02	TRUE
il1r1	Interleukin-1 receptor type 1	p130714	Unspecified Diabetes Mellitus	0.039	0.20	4.77	556	491	67	7.80E-06	2.13E-04	TRUE
il1r1	Interleukin-1 receptor type 1	p130708	Non-Insulin-Dependent Diabetes Mellitus	0.027	0.18	5.99	556	444	114	1.17E-08	7.01E-07	TRUE
il1rl1	Interleukin-1 receptor-like 1	p130770	Deficiency Of Other B Group Vitamins	0.142	0.09	−0.79	566	530	38	4.32E-01	6.58E-01	FALSE
il1rl1	Interleukin-1 receptor-like 1	p131598	Gastritis And Duodenitis	0.138	0.14	0.07	566	457	111	9.42E-01	9.66E-01	FALSE
il1rl1	Interleukin-1 receptor-like 1	p130008	Other Bacterial Intestinal Infections	0.137	0.16	0.18	566	541	27	8.59E-01	9.20E-01	FALSE
il1rl1	Interleukin-1 receptor-like 1	p130774	Vitamin D Deficiency	0.137	0.16	0.33	566	533	35	7.46E-01	8.74E-01	FALSE
il1rl1	Interleukin-1 receptor-like 1	p131560	Diseases Of Pulp And Periapical Tissues	0.135	0.40	0.85	566	560	8	4.21E-01	6.54E-01	FALSE
il1rl1	Interleukin-1 receptor-like 1	p130824	Amyloidosis	0.138	0.30	0.96	566	565	3	4.34E-01	6.58E-01	FALSE
il1rl1	Interleukin-1 receptor-like 1	p131640	Other Functional Intestinal Disorders	0.125	0.18	1.01	566	425	143	3.12E-01	5.54E-01	FALSE
il1rl1	Interleukin-1 receptor-like 1	p131582	Oesophagitis	0.128	0.28	1.21	566	528	40	2.34E-01	4.74E-01	FALSE
il1rl1	Interleukin-1 receptor-like 1	p130814	Disorders Of Lipoprotein Metabolism And Other Lipidaemias	0.113	0.17	1.24	566	301	267	2.15E-01	4.40E-01	FALSE
il1rl1	Interleukin-1 receptor-like 1	p130820	Disorders Of Mineral Metabolism	0.130	0.27	1.29	566	533	35	2.07E-01	4.30E-01	FALSE
il1rl1	Interleukin-1 receptor-like 1	p130828	Other Disorders Of Fluid, Electrolyte And Acid-Base Balance	0.120	0.19	1.42	566	429	139	1.56E-01	3.56E-01	FALSE
il1rl1	Interleukin-1 receptor-like 1	p130826	Volume Depletion	0.121	0.21	1.64	566	451	117	1.03E-01	2.69E-01	FALSE
il1rl1	Interleukin-1 receptor-like 1	p131630	Other Non-Infective Gastro-Enteritis And Colitis	0.123	0.28	1.94	566	511	57	5.68E-02	1.94E-01	FALSE
il1rl1	Interleukin-1 receptor-like 1	p130706	Insulin-Dependent Diabetes Mellitus	0.128	0.46	2.38	566	550	18	2.89E-02	1.26E-01	FALSE
il1rl1	Interleukin-1 receptor-like 1	p130792	Obesity	0.117	0.32	2.54	566	507	61	1.32E-02	7.14E-02	FALSE
il1rl1	Interleukin-1 receptor-like 1	p131654	Other Disorders Of Peritoneum	0.131	0.49	2.60	566	556	12	2.40E-02	1.09E-01	FALSE
il1rl1	Interleukin-1 receptor-like 1	p131650	Haemorrhoids And Perianal Venous Thrombosis	0.117	0.33	2.61	566	512	56	1.13E-02	6.93E-02	FALSE
il1rl1	Interleukin-1 receptor-like 1	p130708	Non-Insulin-Dependent Diabetes Mellitus	0.109	0.26	2.80	566	454	114	5.67E-03	4.48E-02	TRUE
il1rl1	Interleukin-1 receptor-like 1	p130714	Unspecified Diabetes Mellitus	0.115	0.31	2.81	566	500	68	6.14E-03	4.67E-02	TRUE
il1rl1	Interleukin-1 receptor-like 1	p130718	Other Disorders Of Pancreatic Internal Secretion	0.121	0.46	3.12	566	538	30	3.82E-03	3.37E-02	TRUE
ltbp2	Latent-transforming growth factor beta	p130824	Amyloidosis	0.281	−0.04	−1.47	568	567	3	2.79E-01	5.23E-01	FALSE
ltbp2	Latent-transforming growth factor beta	p130770	Deficiency Of Other B Group Vitamins	0.283	0.23	−0.79	568	532	38	4.31E-01	6.58E-01	FALSE
ltbp2	Latent-transforming growth factor beta	p131560	Diseases Of Pulp And Periapical Tissues	0.280	0.21	−0.46	568	562	8	6.62E-01	8.38E-01	FALSE
ltbp2	Latent-transforming growth factor beta	p130814	Disorders Of Lipoprotein Metabolism And Other Lipidaemias	0.279	0.28	0.01	568	302	268	9.94E-01	9.99E-01	FALSE
ltbp2	Latent-transforming growth factor beta	p130708	Non-Insulin-Dependent Diabetes Mellitus	0.278	0.29	0.20	568	455	115	8.38E-01	9.08E-01	FALSE
ltbp2	Latent-transforming growth factor beta	p131654	Other Disorders Of Peritoneum	0.279	0.30	0.25	568	558	12	8.11E-01	9.08E-01	FALSE
ltbp2	Latent-transforming growth factor beta	p131640	Other Functional Intestinal Disorders	0.276	0.29	0.40	568	427	143	6.90E-01	8.50E-01	FALSE
ltbp2	Latent-transforming growth factor beta	p130718	Other Disorders Of Pancreatic Internal Secretion	0.278	0.31	0.55	568	540	30	5.89E-01	7.89E-01	FALSE
ltbp2	Latent-transforming growth factor beta	p131650	Haemorrhoids And Perianal Venous Thrombosis	0.276	0.31	0.73	568	514	56	4.67E-01	6.89E-01	FALSE
ltbp2	Latent-transforming growth factor beta	p130008	Other Bacterial Intestinal Infections	0.274	0.39	0.80	568	543	27	4.31E-01	6.58E-01	FALSE
ltbp2	Latent-transforming growth factor beta	p130774	Vitamin D Deficiency	0.276	0.34	0.86	568	535	35	3.94E-01	6.33E-01	FALSE
ltbp2	Latent-transforming growth factor beta	p130820	Disorders Of Mineral Metabolism	0.275	0.34	1.02	568	535	35	3.14E-01	5.54E-01	FALSE
ltbp2	Latent-transforming growth factor beta	p130792	Obesity	0.271	0.35	1.05	568	508	62	2.95E-01	5.33E-01	FALSE
ltbp2	Latent-transforming growth factor beta	p131582	Oesophagitis	0.272	0.38	1.07	568	530	40	2.90E-01	5.31E-01	FALSE
ltbp2	Latent-transforming growth factor beta	p130714	Unspecified Diabetes Mellitus	0.270	0.35	1.12	568	501	69	2.65E-01	5.12E-01	FALSE
ltbp2	Latent-transforming growth factor beta	p131630	Other Non-Infective Gastro-Enteritis And Colitis	0.270	0.36	1.17	568	512	58	2.48E-01	4.96E-01	FALSE
ltbp2	Latent-transforming growth factor beta	p131598	Gastritis And Duodenitis	0.268	0.33	1.47	568	459	111	1.44E-01	3.38E-01	FALSE
ltbp2	Latent-transforming growth factor beta	p130826	Volume Depletion	0.263	0.34	1.67	568	453	117	9.77E-02	2.64E-01	FALSE
ltbp2	Latent-transforming growth factor beta	p130706	Insulin-Dependent Diabetes Mellitus	0.272	0.50	2.30	568	552	18	3.35E-02	1.38E-01	FALSE
ltbp2	Latent-transforming growth factor beta	p130828	Other Disorders Of Fluid, Electrolyte And Acid-Base Balance	0.243	0.39	4.08	568	431	139	6.02E-05	1.13E-03	TRUE
timp4	Metalloproteinase inhibitor 4	p130714	Unspecified Diabetes Mellitus	0.212	0.11	−1.52	558	492	68	1.34E-01	3.23E-01	FALSE
timp4	Metalloproteinase inhibitor 4	p130708	Non-Insulin-Dependent Diabetes Mellitus	0.214	0.14	−1.38	558	446	114	1.70E-01	3.78E-01	FALSE
timp4	Metalloproteinase inhibitor 4	p131598	Gastritis And Duodenitis	0.210	0.15	−1.08	558	452	108	2.82E-01	5.23E-01	FALSE
timp4	Metalloproteinase inhibitor 4	p130824	Amyloidosis	0.200	−0.02	−0.62	558	557	3	5.96E-01	7.91E-01	FALSE
timp4	Metalloproteinase inhibitor 4	p130814	Disorders Of Lipoprotein Metabolism And Other Lipidaemias	0.209	0.19	−0.53	558	294	266	5.95E-01	7.91E-01	FALSE
timp4	Metalloproteinase inhibitor 4	p130718	Other Disorders Of Pancreatic Internal Secretion	0.201	0.17	−0.22	558	530	30	8.28E-01	9.08E-01	FALSE
timp4	Metalloproteinase inhibitor 4	p130826	Volume Depletion	0.200	0.20	−0.07	558	445	115	9.43E-01	9.66E-01	FALSE
timp4	Metalloproteinase inhibitor 4	p131650	Haemorrhoids And Perianal Venous Thrombosis	0.199	0.20	0.09	558	504	56	9.29E-01	9.57E-01	FALSE
timp4	Metalloproteinase inhibitor 4	p130706	Insulin-Dependent Diabetes Mellitus	0.198	0.24	0.30	558	542	18	7.67E-01	8.88E-01	FALSE
timp4	Metalloproteinase inhibitor 4	p131560	Diseases Of Pulp And Periapical Tissues	0.198	0.29	0.40	558	552	8	7.03E-01	8.50E-01	FALSE
timp4	Metalloproteinase inhibitor 4	p130008	Other Bacterial Intestinal Infections	0.197	0.24	0.45	558	534	26	6.53E-01	8.38E-01	FALSE
timp4	Metalloproteinase inhibitor 4	p131654	Other Disorders Of Peritoneum	0.196	0.34	0.96	558	548	12	3.56E-01	5.81E-01	FALSE
timp4	Metalloproteinase inhibitor 4	p131582	Oesophagitis	0.194	0.27	0.99	558	521	39	3.30E-01	5.54E-01	FALSE
timp4	Metalloproteinase inhibitor 4	p131630	Other Non-Infective Gastro-Enteritis And Colitis	0.191	0.27	1.28	558	504	56	2.05E-01	4.30E-01	FALSE
timp4	Metalloproteinase inhibitor 4	p130770	Deficiency Of Other B Group Vitamins	0.192	0.30	1.35	558	523	37	1.85E-01	4.05E-01	FALSE
timp4	Metalloproteinase inhibitor 4	p130820	Disorders Of Mineral Metabolism	0.189	0.36	1.75	558	525	35	8.75E-02	2.53E-01	FALSE
timp4	Metalloproteinase inhibitor 4	p131640	Other Functional Intestinal Disorders	0.173	0.28	2.01	558	419	141	4.56E-02	1.71E-01	FALSE
timp4	Metalloproteinase inhibitor 4	p130792	Obesity	0.183	0.33	2.06	558	498	62	4.32E-02	1.66E-01	FALSE
timp4	Metalloproteinase inhibitor 4	p130774	Vitamin D Deficiency	0.187	0.38	2.22	558	526	34	3.28E-02	1.37E-01	FALSE
timp4	Metalloproteinase inhibitor 4	p130828	Other Disorders Of Fluid, Electrolyte And Acid-Base Balance	0.169	0.29	2.47	558	423	137	1.42E-02	7.32E-02	FALSE
tcn2	Transcobalamin-2	p130824	Amyloidosis	0.081	−0.08	−1.37	568	567	3	2.99E-01	5.38E-01	FALSE
tcn2	Transcobalamin-2	p131560	Diseases Of Pulp And Periapical Tissues	0.082	−0.05	−0.86	568	562	8	4.19E-01	6.54E-01	FALSE
tcn2	Transcobalamin-2	p130008	Other Bacterial Intestinal Infections	0.082	0.04	−0.51	568	543	27	6.13E-01	8.01E-01	FALSE
tcn2	Transcobalamin-2	p131582	Oesophagitis	0.083	0.05	−0.44	568	530	40	6.61E-01	8.38E-01	FALSE
tcn2	Transcobalamin-2	p130774	Vitamin D Deficiency	0.082	0.06	−0.40	568	535	35	6.93E-01	8.50E-01	FALSE
tcn2	Transcobalamin-2	p131640	Other Functional Intestinal Disorders	0.083	0.07	−0.20	568	427	143	8.39E-01	9.08E-01	FALSE
tcn2	Transcobalamin-2	p131630	Other Non-Infective Gastro-Enteritis And Colitis	0.081	0.08	−0.12	568	512	58	9.03E-01	9.41E-01	FALSE
tcn2	Transcobalamin-2	p131598	Gastritis And Duodenitis	0.075	0.10	0.60	568	459	111	5.51E-01	7.55E-01	FALSE
tcn2	Transcobalamin-2	p130770	Deficiency Of Other B Group Vitamins	0.078	0.12	0.72	568	532	38	4.77E-01	6.97E-01	FALSE
tcn2	Transcobalamin-2	p130820	Disorders Of Mineral Metabolism	0.077	0.14	0.73	568	535	35	4.68E-01	6.89E-01	FALSE
tcn2	Transcobalamin-2	p130826	Volume Depletion	0.066	0.14	1.55	568	453	117	1.22E-01	3.01E-01	FALSE
tcn2	Transcobalamin-2	p131650	Haemorrhoids And Perianal Venous Thrombosis	0.072	0.16	1.59	568	514	56	1.16E-01	2.94E-01	FALSE
tcn2	Transcobalamin-2	p130814	Disorders Of Lipoprotein Metabolism And Other Lipidaemias	0.053	0.11	1.69	568	302	268	9.15E-02	2.54E-01	FALSE
tcn2	Transcobalamin-2	p130718	Other Disorders Of Pancreatic Internal Secretion	0.074	0.20	1.76	568	540	30	8.84E-02	2.53E-01	FALSE
tcn2	Transcobalamin-2	p130714	Unspecified Diabetes Mellitus	0.063	0.21	2.47	568	501	69	1.57E-02	7.99E-02	FALSE
tcn2	Transcobalamin-2	p130792	Obesity	0.062	0.23	2.56	568	508	62	1.26E-02	7.14E-02	FALSE
tcn2	Transcobalamin-2	p130828	Other Disorders Of Fluid, Electrolyte And Acid-Base Balance	0.053	0.17	2.69	568	431	139	7.73E-03	5.27E-02	FALSE
tcn2	Transcobalamin-2	p130708	Non-Insulin-Dependent Diabetes Mellitus	0.057	0.17	2.71	568	455	115	7.48E-03	5.22E-02	FALSE
tcn2	Transcobalamin-2	p131654	Other Disorders Of Peritoneum	0.076	0.29	2.90	568	558	12	1.30E-02	7.14E-02	FALSE
tcn2	Transcobalamin-2	p130706	Insulin-Dependent Diabetes Mellitus	0.073	0.32	3.03	568	552	18	6.94E-03	5.08E-02	FALSE

AD: Alzheimer’s disease

P_VAL_FDR_CORRECTED: p-value after False Discovery Rate corrected

**Table 6.** Proteomic biomarker comparison in isolated PD cases vs. cases with digestive, endocrine, metabolic, and nutritional conditions

Olink_marker_being_compared	Olink_marker_definition	ICD10_Code	ICD10_Code_definition	Average Olink_marker level in individuals diagnosed with only PD (PD)	Average Olink_marker level in individuals diagnosed with both PD and ICD-10 code (PD + ICD-10)	T-Statistic (PD + ICD-10 vs. PD)	Degrees of Free	AD_size	AD_ICD10_size	P-Value	P_VAL_FDR_CORRECTED	FDR_CORRECTED_rejected
Collagenase 3	mmp13	p130714	unspecified diabetes mellitus	0.06	−0.06	−2.24	603	548	57	2.82E-02	1.69E-01	FALSE
Collagenase 3	mmp13	p130792	obesity	0.06	−0.04	−2.19	603	532	73	3.08E-02	1.77E-01	FALSE
Collagenase 3	mmp13	p130708	non-insulin-dependent diabetes mellitus	0.06	−0.02	−2.16	603	504	101	3.21E-02	1.78E-01	FALSE
Collagenase 3	mmp13	p130706	insulin-dependent diabetes mellitus	0.05	−0.10	−1.47	603	590	15	1.63E-01	4.34E-01	FALSE
Collagenase 3	mmp13	p130718	other disorders of pancreatic internal secretion	0.05	−0.05	−1.24	603	580	25	2.25E-01	5.15E-01	FALSE
Collagenase 3	mmp13	p130770	deficiency of other b group vitamins	0.05	−0.03	−1.17	603	578	27	2.51E-01	5.54E-01	FALSE
Collagenase 3	mmp13	p131648	other diseases of intestine	0.05	0.01	−0.82	603	547	58	4.17E-01	6.53E-01	FALSE
Collagenase 3	mmp13	p131640	other functional intestinal disorders	0.04	0.06	0.69	603	407	198	4.87E-01	6.95E-01	FALSE
Collagenase 3	mmp13	p131654	other disorders of peritoneum	0.05	0.13	0.95	603	596	9	3.69E-01	6.53E-01	FALSE
Collagenase 3	mmp13	p131636	diverticular disease of intestine	0.04	0.08	1.06	603	467	138	2.91E-01	5.89E-01	FALSE
Collagenase 3	mmp13	p131650	haemorrhoids and perianal venous thrombosis	0.04	0.10	1.23	603	538	67	2.23E-01	5.15E-01	FALSE
Collagenase 3	mmp13	p131600	dyspepsia	0.04	0.23	3.28	603	569	36	2.08E-03	4.97E-02	TRUE
Neurofilament light polypeptide	nefl	p131650	haemorrhoids and perianal venous thrombosis	0.37	0.23	−2.06	601	536	67	4.23E-02	2.10E-01	FALSE
Neurofilament light polypeptide	nefl	p130792	obesity	0.37	0.26	−1.35	601	530	73	1.80E-01	4.63E-01	FALSE
Neurofilament light polypeptide	nefl	p130706	insulin-dependent diabetes mellitus	0.35	0.35	−0.03	601	588	15	9.79E-01	9.92E-01	FALSE
Neurofilament light polypeptide	nefl	p131600	dyspepsia	0.35	0.38	0.25	601	567	36	8.02E-01	9.16E-01	FALSE
Neurofilament light polypeptide	nefl	p131640	other functional intestinal disorders	0.35	0.37	0.45	601	406	197	6.50E-01	8.24E-01	FALSE
Neurofilament light polypeptide	nefl	p130714	unspecified diabetes mellitus	0.35	0.43	0.84	601	547	56	4.04E-01	6.53E-01	FALSE
Neurofilament light polypeptide	nefl	p131654	other disorders of peritoneum	0.35	0.51	0.86	601	594	9	4.15E-01	6.53E-01	FALSE
Neurofilament light polypeptide	nefl	p130770	deficiency of other b group vitamins	0.35	0.48	0.88	601	576	27	3.87E-01	6.53E-01	FALSE
Neurofilament light polypeptide	nefl	p130708	non-insulin-dependent diabetes mellitus	0.34	0.40	0.91	601	503	100	3.63E-01	6.53E-01	FALSE
Neurofilament light polypeptide	nefl	p130718	other disorders of pancreatic internal secretion	0.35	0.45	0.93	601	578	25	3.63E-01	6.53E-01	FALSE
Neurofilament light polypeptide	nefl	p131636	diverticular disease of intestine	0.33	0.42	1.49	601	466	137	1.37E-01	3.87E-01	FALSE
Neurofilament light polypeptide	nefl	p131648	other diseases of intestine	0.34	0.46	1.56	601	545	58	1.22E-01	3.82E-01	FALSE
Protein-arginine deiminase type-2	padi2	p130770	deficiency of other b group vitamins	0.19	−0.01	−2.77	608	579	31	7.94E-03	9.52E-02	FALSE
Protein-arginine deiminase type-2	padi2	p131600	dyspepsia	0.19	0.13	−0.76	608	573	37	4.52E-01	6.85E-01	FALSE
Protein-arginine deiminase type-2	padi2	p131640	other functional intestinal disorders	0.19	0.17	−0.36	608	415	195	7.16E-01	8.52E-01	FALSE
Protein-arginine deiminase type-2	padi2	p130718	other disorders of pancreatic internal secretion	0.18	0.18	−0.07	608	587	23	9.43E-01	9.69E-01	FALSE
Protein-arginine deiminase type-2	padi2	p131648	other diseases of intestine	0.18	0.24	0.65	608	549	61	5.18E-01	7.17E-01	FALSE
Protein-arginine deiminase type-2	padi2	p130714	unspecified diabetes mellitus	0.18	0.26	0.67	608	554	56	5.06E-01	7.09E-01	FALSE
Protein-arginine deiminase type-2	padi2	p130792	obesity	0.18	0.24	0.75	608	531	79	4.57E-01	6.86E-01	FALSE
Protein-arginine deiminase type-2	padi2	p130706	insulin-dependent diabetes mellitus	0.18	0.41	0.82	608	595	15	4.25E-01	6.53E-01	FALSE
Protein-arginine deiminase type-2	padi2	p131636	diverticular disease of intestine	0.17	0.24	0.86	608	469	141	3.92E-01	6.53E-01	FALSE
Protein-arginine deiminase type-2	padi2	p131654	other disorders of peritoneum	0.17	0.72	1.04	608	600	10	3.27E-01	6.34E-01	FALSE
Protein-arginine deiminase type-2	padi2	p131650	haemorrhoids and perianal venous thrombosis	0.17	0.31	1.11	608	538	72	2.72E-01	5.76E-01	FALSE
Protein-arginine deiminase type-2	padi2	p130708	non-insulin-dependent diabetes mellitus	0.15	0.33	1.65	608	508	102	1.02E-01	3.41E-01	FALSE
Interleukin-27	ebi3_il27	p131650	haemorrhoids and perianal venous thrombosis	0.13	0.04	−2.43	621	551	72	1.66E-02	1.33E-01	FALSE
Interleukin-27	ebi3_il27	p130792	obesity	0.12	0.11	−0.14	621	543	80	8.87E-01	9.40E-01	FALSE
Interleukin-27	ebi3_il27	p131600	dyspepsia	0.12	0.12	−0.01	621	586	37	9.92E-01	9.92E-01	FALSE
Interleukin-27	ebi3_il27	p131636	diverticular disease of intestine	0.12	0.13	0.29	621	480	143	7.70E-01	9.07E-01	FALSE
Interleukin-27	ebi3_il27	p130770	deficiency of other b group vitamins	0.12	0.16	0.88	621	594	29	3.87E-01	6.53E-01	FALSE
Interleukin-27	ebi3_il27	p131648	other diseases of intestine	0.11	0.16	0.93	621	561	62	3.56E-01	6.53E-01	FALSE
Interleukin-27	ebi3_il27	p131654	other disorders of peritoneum	0.12	0.23	1.15	621	613	10	2.80E-01	5.85E-01	FALSE
Interleukin-27	ebi3_il27	p131640	other functional intestinal disorders	0.10	0.14	1.30	621	420	203	1.93E-01	4.66E-01	FALSE
Interleukin-27	ebi3_il27	p130708	non-insulin-dependent diabetes mellitus	0.11	0.18	1.95	621	519	104	5.34E-02	2.25E-01	FALSE
Interleukin-27	ebi3_il27	p130714	unspecified diabetes mellitus	0.10	0.26	2.65	621	566	57	1.01E-02	1.04E-01	FALSE
Interleukin-27	ebi3_il27	p130718	other disorders of pancreatic internal secretion	0.11	0.37	2.71	621	597	26	1.17E-02	1.12E-01	FALSE
Interleukin-27	ebi3_il27	p130706	insulin-dependent diabetes mellitus	0.11	0.47	3.14	621	606	17	6.13E-03	8.03E-02	FALSE
Latexin	lxn	p130706	insulin-dependent diabetes mellitus	0.24	−0.14	−1.94	611	597	16	6.94E-02	2.69E-01	FALSE
Latexin	lxn	p130718	other disorders of pancreatic internal secretion	0.25	0.00	−1.36	611	588	25	1.85E-01	4.66E-01	FALSE
Latexin	lxn	p130770	deficiency of other b group vitamins	0.24	0.08	−0.87	611	584	29	3.94E-01	6.53E-01	FALSE
Latexin	lxn	p130714	unspecified diabetes mellitus	0.24	0.15	−0.71	611	555	58	4.79E-01	6.93E-01	FALSE
Latexin	lxn	p130792	obesity	0.24	0.21	−0.29	611	537	76	7.74E-01	9.07E-01	FALSE
Latexin	lxn	p130708	non-insulin-dependent diabetes mellitus	0.24	0.22	−0.19	611	510	103	8.47E-01	9.40E-01	FALSE
Latexin	lxn	p131636	diverticular disease of intestine	0.24	0.22	−0.16	611	472	141	8.76E-01	9.40E-01	FALSE
Latexin	lxn	p131650	haemorrhoids and perianal venous thrombosis	0.23	0.24	0.10	611	542	71	9.21E-01	9.61E-01	FALSE
Latexin	lxn	p131640	other functional intestinal disorders	0.23	0.24	0.18	611	412	201	8.56E-01	9.40E-01	FALSE
Latexin	lxn	p131600	dyspepsia	0.23	0.31	0.48	611	577	36	6.35E-01	8.24E-01	FALSE
Latexin	lxn	p131648	other diseases of intestine	0.23	0.33	0.82	611	554	59	4.16E-01	6.53E-01	FALSE
Latexin	lxn	p131654	other disorders of peritoneum	0.23	0.73	1.43	611	604	9	1.89E-01	4.66E-01	FALSE
Peroxiredoxin-1	prdx1	p130706	insulin-dependent diabetes mellitus	0.22	−0.43	−3.60	603	589	16	2.13E-03	4.97E-02	TRUE
Peroxiredoxin-1	prdx1	p130718	other disorders of pancreatic internal secretion	0.22	−0.20	−2.07	603	580	25	4.78E-02	2.22E-01	FALSE
Peroxiredoxin-1	prdx1	p130770	deficiency of other b group vitamins	0.21	−0.12	−1.94	603	577	28	6.18E-02	2.47E-01	FALSE
Peroxiredoxin-1	prdx1	p131640	other functional intestinal disorders	0.23	0.14	−0.88	603	405	200	3.82E-01	6.53E-01	FALSE
Peroxiredoxin-1	prdx1	p130714	unspecified diabetes mellitus	0.21	0.07	−0.85	603	547	58	4.00E-01	6.53E-01	FALSE
Peroxiredoxin-1	prdx1	p131600	dyspepsia	0.20	0.13	−0.42	603	569	36	6.75E-01	8.34E-01	FALSE
Peroxiredoxin-1	prdx1	p130708	non-insulin-dependent diabetes mellitus	0.20	0.19	−0.11	603	503	102	9.13E-01	9.60E-01	FALSE
Peroxiredoxin-1	prdx1	p131650	haemorrhoids and perianal venous thrombosis	0.20	0.22	0.17	603	534	71	8.68E-01	9.40E-01	FALSE
Peroxiredoxin-1	prdx1	p130792	obesity	0.19	0.22	0.20	603	529	76	8.40E-01	9.40E-01	FALSE
Peroxiredoxin-1	prdx1	p131636	diverticular disease of intestine	0.16	0.31	1.30	603	465	140	1.94E-01	4.66E-01	FALSE
Peroxiredoxin-1	prdx1	p131648	other diseases of intestine	0.17	0.45	1.47	603	548	57	1.46E-01	4.05E-01	FALSE
Peroxiredoxin-1	prdx1	p131654	other disorders of peritoneum	0.19	0.69	1.67	603	596	9	1.32E-01	3.87E-01	FALSE
CD276 antigen	cd276	p131650	haemorrhoids and perianal venous thrombosis	0.12	0.02	−1.95	623	553	72	5.47E-02	2.25E-01	FALSE
CD276 antigen	cd276	p131600	dyspepsia	0.11	0.04	−1.03	623	589	36	3.10E-01	6.12E-01	FALSE
CD276 antigen	cd276	p130792	obesity	0.11	0.09	−0.46	623	546	79	6.44E-01	8.24E-01	FALSE
CD276 antigen	cd276	p131648	other diseases of intestine	0.11	0.08	−0.45	623	562	63	6.52E-01	8.24E-01	FALSE
CD276 antigen	cd276	p131654	other disorders of peritoneum	0.11	0.05	−0.38	623	615	10	7.10E-01	8.52E-01	FALSE
CD276 antigen	cd276	p131636	diverticular disease of intestine	0.11	0.10	−0.15	623	483	142	8.79E-01	9.40E-01	FALSE
CD276 antigen	cd276	p130770	deficiency of other b group vitamins	0.11	0.11	0.06	623	596	29	9.53E-01	9.73E-01	FALSE
CD276 antigen	cd276	p131640	other functional intestinal disorders	0.09	0.15	1.99	623	424	201	4.68E-02	2.22E-01	FALSE
CD276 antigen	cd276	p130718	other disorders of pancreatic internal secretion	0.09	0.48	3.08	623	601	24	5.18E-03	7.46E-02	FALSE
CD276 antigen	cd276	p130708	non-insulin-dependent diabetes mellitus	0.08	0.23	3.38	623	523	102	9.29E-04	4.33E-02	TRUE
CD276 antigen	cd276	p130714	unspecified diabetes mellitus	0.09	0.33	3.40	623	569	56	1.20E-03	4.33E-02	TRUE
CD276 antigen	cd276	p130706	insulin-dependent diabetes mellitus	0.09	0.70	4.38	623	609	16	5.06E-04	3.64E-02	TRUE
Epithelial cell adhesion molecule	epcam	p130714	unspecified diabetes mellitus	0.12	−0.24	−2.70	621	568	55	8.69E-03	9.62E-02	FALSE
Epithelial cell adhesion molecule	epcam	p130770	deficiency of other b group vitamins	0.10	−0.30	−2.39	621	594	29	2.29E-02	1.57E-01	FALSE
Epithelial cell adhesion molecule	epcam	p130792	obesity	0.11	−0.09	−1.67	621	545	78	9.72E-02	3.41E-01	FALSE
Epithelial cell adhesion molecule	epcam	p131648	other diseases of intestine	0.10	−0.08	−1.22	621	560	63	2.27E-01	5.15E-01	FALSE
Epithelial cell adhesion molecule	epcam	p130708	non-insulin-dependent diabetes mellitus	0.10	0.02	−0.71	621	522	101	4.81E-01	6.93E-01	FALSE
Epithelial cell adhesion molecule	epcam	p130706	insulin-dependent diabetes mellitus	0.08	0.14	0.19	621	608	15	8.53E-01	9.40E-01	FALSE
Epithelial cell adhesion molecule	epcam	p131640	other functional intestinal disorders	0.08	0.10	0.25	621	423	200	8.00E-01	9.16E-01	FALSE
Epithelial cell adhesion molecule	epcam	p131654	other disorders of peritoneum	0.08	0.23	0.28	621	613	10	7.84E-01	9.11E-01	FALSE
Epithelial cell adhesion molecule	epcam	p131650	haemorrhoids and perianal venous thrombosis	0.08	0.15	0.59	621	551	72	5.57E-01	7.55E-01	FALSE
Epithelial cell adhesion molecule	epcam	p131636	diverticular disease of intestine	0.05	0.21	1.50	621	481	142	1.35E-01	3.87E-01	FALSE
Epithelial cell adhesion molecule	epcam	p131600	dyspepsia	0.06	0.50	2.14	621	587	36	3.84E-02	1.98E-01	FALSE
Epithelial cell adhesion molecule	epcam	p130718	other disorders of pancreatic internal secretion	0.07	0.51	2.21	621	599	24	3.61E-02	1.92E-01	FALSE
Tyrosine-protein kinase Mer	mertk	p131650	haemorrhoids and perianal venous thrombosis	0.11	0.08	−0.98	629	558	73	3.30E-01	6.34E-01	FALSE
Tyrosine-protein kinase Mer	mertk	p131600	dyspepsia	0.11	0.07	−0.84	629	594	37	4.03E-01	6.53E-01	FALSE
Tyrosine-protein kinase Mer	mertk	p131648	other diseases of intestine	0.11	0.09	−0.58	629	569	62	5.61E-01	7.55E-01	FALSE
Tyrosine-protein kinase Mer	mertk	p131636	diverticular disease of intestine	0.11	0.11	0.15	629	487	144	8.82E-01	9.40E-01	FALSE
Tyrosine-protein kinase Mer	mertk	p131640	other functional intestinal disorders	0.11	0.12	0.42	629	427	204	6.78E-01	8.34E-01	FALSE
Tyrosine-protein kinase Mer	mertk	p130714	unspecified diabetes mellitus	0.11	0.13	0.60	629	573	58	5.50E-01	7.55E-01	FALSE
Tyrosine-protein kinase Mer	mertk	p130792	obesity	0.10	0.15	1.07	629	551	80	2.90E-01	5.89E-01	FALSE
Tyrosine-protein kinase Mer	mertk	p130770	deficiency of other b group vitamins	0.10	0.21	1.77	629	600	31	8.52E-02	3.07E-01	FALSE
Tyrosine-protein kinase Mer	mertk	p131654	other disorders of peritoneum	0.11	0.25	1.78	629	621	10	1.07E-01	3.52E-01	FALSE
Tyrosine-protein kinase Mer	mertk	p130708	non-insulin-dependent diabetes mellitus	0.10	0.16	1.94	629	527	104	5.43E-02	2.25E-01	FALSE
Tyrosine-protein kinase Mer	mertk	p130718	other disorders of pancreatic internal secretion	0.10	0.25	2.34	629	606	25	2.74E-02	1.69E-01	FALSE
Tyrosine-protein kinase Mer	mertk	p130706	insulin-dependent diabetes mellitus	0.10	0.28	2.62	629	615	16	1.86E-02	1.34E-01	FALSE
Desmoglein-2	dsg2	p130708	non-insulin-dependent diabetes mellitus	0.04	−0.05	−2.45	620	518	104	1.57E-02	1.33E-01	FALSE
Desmoglein-2	dsg2	p130714	unspecified diabetes mellitus	0.03	−0.06	−1.84	620	565	57	7.09E-02	2.69E-01	FALSE
Desmoglein-2	dsg2	p130792	obesity	0.03	−0.02	−1.35	620	544	78	1.80E-01	4.63E-01	FALSE
Desmoglein-2	dsg2	p130770	deficiency of other b group vitamins	0.03	−0.02	−1.14	620	594	28	2.64E-01	5.68E-01	FALSE
Desmoglein-2	dsg2	p131600	dyspepsia	0.03	0.00	−0.55	620	588	34	5.84E-01	7.78E-01	FALSE
Desmoglein-2	dsg2	p131650	haemorrhoids and perianal venous thrombosis	0.03	0.01	−0.49	620	552	70	6.27E-01	8.21E-01	FALSE
Desmoglein-2	dsg2	p131636	diverticular disease of intestine	0.02	0.04	0.66	620	481	141	5.07E-01	7.09E-01	FALSE
Desmoglein-2	dsg2	p131648	other diseases of intestine	0.02	0.06	0.96	620	561	61	3.40E-01	6.45E-01	FALSE
Desmoglein-2	dsg2	p130718	other disorders of pancreatic internal secretion	0.02	0.09	1.05	620	597	25	3.05E-01	6.11E-01	FALSE
Desmoglein-2	dsg2	p131640	other functional intestinal disorders	0.01	0.05	1.52	620	420	202	1.29E-01	3.87E-01	FALSE
Desmoglein-2	dsg2	p131654	other disorders of peritoneum	0.02	0.18	1.55	620	613	9	1.59E-01	4.33E-01	FALSE
Desmoglein-2	dsg2	p130706	insulin-dependent diabetes mellitus	0.02	0.25	2.47	620	606	16	2.55E-02	1.67E-01	FALSE
Interleukin-1 receptor-like 1	il1rl1	p130770	deficiency of other b group vitamins	0.15	0.08	−0.82	631	602	31	4.20E-01	6.53E-01	FALSE
Interleukin-1 receptor-like 1	il1rl1	p131636	diverticular disease of intestine	0.16	0.14	−0.39	631	488	145	6.94E-01	8.40E-01	FALSE
Interleukin-1 receptor-like 1	il1rl1	p131600	dyspepsia	0.15	0.13	−0.24	631	596	37	8.11E-01	9.20E-01	FALSE
Interleukin-1 receptor-like 1	il1rl1	p130792	obesity	0.15	0.15	−0.01	631	552	81	9.91E-01	9.92E-01	FALSE
Interleukin-1 receptor-like 1	il1rl1	p131654	other disorders of peritoneum	0.15	0.17	0.09	631	624	9	9.28E-01	9.61E-01	FALSE
Interleukin-1 receptor-like 1	il1rl1	p131640	other functional intestinal disorders	0.14	0.16	0.41	631	427	206	6.83E-01	8.34E-01	FALSE
Interleukin-1 receptor-like 1	il1rl1	p131650	haemorrhoids and perianal venous thrombosis	0.14	0.22	1.15	631	561	72	2.54E-01	5.54E-01	FALSE
Interleukin-1 receptor-like 1	il1rl1	p131648	other diseases of intestine	0.14	0.26	1.67	631	570	63	9.99E-02	3.41E-01	FALSE
Interleukin-1 receptor-like 1	il1rl1	p130718	other disorders of pancreatic internal secretion	0.14	0.37	2.01	631	607	26	5.46E-02	2.25E-01	FALSE
Interleukin-1 receptor-like 1	il1rl1	p130706	insulin-dependent diabetes mellitus	0.14	0.46	2.61	631	616	17	1.83E-02	1.34E-01	FALSE
Interleukin-1 receptor-like 1	il1rl1	p130708	non-insulin-dependent diabetes mellitus	0.12	0.29	3.00	631	527	106	3.08E-03	5.55E-02	FALSE
Interleukin-1 receptor-like 1	il1rl1	p130714	unspecified diabetes mellitus	0.13	0.39	3.92	631	575	58	1.99E-04	2.87E-02	TRUE
Neural cell adhesion molecule 1	ncam1	p130792	obesity	0.13	−0.03	−3.12	628	550	80	2.42E-03	4.97E-02	TRUE
Neural cell adhesion molecule 1	ncam1	p130714	unspecified diabetes mellitus	0.12	−0.03	−2.97	628	571	59	4.02E-03	6.43E-02	FALSE
Neural cell adhesion molecule 1	ncam1	p130708	non-insulin-dependent diabetes mellitus	0.12	0.03	−2.45	628	525	105	1.55E-02	1.33E-01	FALSE
Neural cell adhesion molecule 1	ncam1	p131600	dyspepsia	0.11	0.01	−1.81	628	595	35	7.80E-02	2.88E-01	FALSE
Neural cell adhesion molecule 1	ncam1	p131636	diverticular disease of intestine	0.12	0.06	−1.58	628	485	145	1.15E-01	3.67E-01	FALSE
Neural cell adhesion molecule 1	ncam1	p130770	deficiency of other b group vitamins	0.11	0.05	−0.81	628	600	30	4.26E-01	6.53E-01	FALSE
Neural cell adhesion molecule 1	ncam1	p130706	insulin-dependent diabetes mellitus	0.11	0.04	−0.74	628	613	17	4.68E-01	6.87E-01	FALSE
Neural cell adhesion molecule 1	ncam1	p131640	other functional intestinal disorders	0.10	0.11	0.43	628	426	204	6.67E-01	8.34E-01	FALSE
Neural cell adhesion molecule 1	ncam1	p131654	other disorders of peritoneum	0.10	0.18	0.51	628	621	9	6.26E-01	8.21E-01	FALSE
Neural cell adhesion molecule 1	ncam1	p131648	other diseases of intestine	0.10	0.14	0.74	628	567	63	4.63E-01	6.87E-01	FALSE
Neural cell adhesion molecule 1	ncam1	p130718	other disorders of pancreatic internal secretion	0.10	0.20	1.23	628	604	26	2.29E-01	5.15E-01	FALSE
Neural cell adhesion molecule 1	ncam1	p131650	haemorrhoids and perianal venous thrombosis	0.10	0.17	1.52	628	559	71	1.32E-01	3.87E-01	FALSE

PD: Parkinson’s disease

P_VAL_FDR_CORRECTED: p-value after False Discovery Rate corrected

## Data Availability

This paper analyzes existing, publicly available data. In addition, complete summary statistics describing these data/processed datasets derived from these data have been deposited in the supplementary materials connected to this publication and are publicly available as of the date of publication.
