# Supplementary material for: Gut-Brain Nexus: Mapping Multi-Modal Links to Neurodegeneration at Biobank Scale
Source: medRxiv. 2024 Nov 6:2024.09.12.24313490. Originally published 2024 Sep 13. Preprint. [Version 2] doi: 10.1101/2024.09.12.24313490 (PMC11451806; doi:10.1101/2024.09.12.24313490)
Supplement: Supplement 1 [file media-1.pdf]

Supplementary Figure 1

Prior_Condition_Description	HR	95% CI	P_VAL
Other Disorders of Peritoneum	0.59	0.44 - 0.81	8.77e-04
Haemorrhoids and Perianal Venous Thrombosis	0.69	0.59 - 0.80	6.94e-07
Obesity	0.84	0.74 - 0.96	9.29e-03
Disorders of Lipoprotein Metabolism and Other Lipidaemias	1.18	1.09 - 1.27	1.53e-05
Gastritis and Duodenitis	1.20	1.09 - 1.32	1.45e-04
Oesophagitis	1.28	1.10 - 1.49	1.63e-03
Disorders of Mineral Metabolism	1.32	1.08 - 1.62	7.75e-03
Other Non-Infective Gastro-Enteritis and Colitis	1.37	1.20 - 1.56	1.75e-06
Diseases of Pulp and Periapical Tissues	1.44	1.11 - 1.87	6.21e-03
Non-Insulin-Dependent Diabetes Mellitus	1.47	1.33 - 1.63	1.19e-14
Other Disorders of Fluid, Electrolyte and Acid-Base Balance	1.51	1.34 - 1.70	6.43e-12
Other Bacterial Intestinal Infections	1.51	1.21 - 1.89	2.35e-04
Unspecified Diabetes Mellitus	1.53	1.33 - 1.75	1.21e-09
Other Functional Intestinal Disorders	1.55	1.40 - 1.73	3.55e-16
Deficiency of Other b Group Vitamins	1.78	1.40 - 2.27	2.61e-06
Volume Depletion	1.78	1.52 - 2.10	3.26e-12
Vitamin d Deficiency	1.95	1.57 - 2.41	9.97e-10
Other Disorders of Pancreatic Internal Secretion	2.23	1.75 - 2.83	5.27e-11
Amyloidosis	2.57	1.34 - 4.94	4.72e-03
Insulin-Dependent Diabetes Mellitus	3.09	2.45 - 3.90	1.77e-21

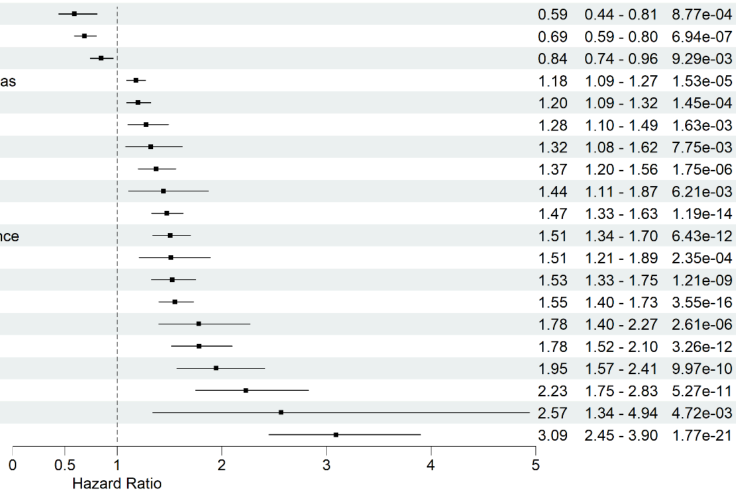

Fig. S1

Forest plot showing hazard ratios for the prior ICD-10 diagnoses that are significantly associated with the risk of Alzheimer’s Disease (AD). (AD ~ ICD-10 + sex + age + Townsend deprivation index)

Supplementary Figure 2

Prior_Condition_Description	HR	95% CI	P_VAL
Haemorrhoids and Perianal Venous Thrombosis	0.59	0.50 - 0.71	3.62e-09
Other Disorders of Peritoneum	0.63	0.45 - 0.87	5.81e-03
Diverticular Disease of Intestine	0.69	0.62 - 0.77	6.24e-11
Other Diseases of Intestine	0.69	0.59 - 0.80	1.34e-06
Obesity	0.82	0.71 - 0.94	5.86e-03
Non-Insulin-Dependent Diabetes Mellitus	1.21	1.08 - 1.36	1.36e-03
Dyspepsia	1.34	1.13 - 1.60	8.51e-04
Other Functional Intestinal Disorders	1.56	1.38 - 1.76	4.81e-13
Unspecified Diabetes Mellitus	1.61	1.39 - 1.86	1.81e-10
Deficiency of Other b Group Vitamins	1.72	1.30 - 2.29	1.88e-04
Other Disorders of Pancreatic Internal Secretion	1.84	1.36 - 2.47	6.76e-05
Insulin-Dependent Diabetes Mellitus	2.65	2.02 - 3.48	2.44e-12

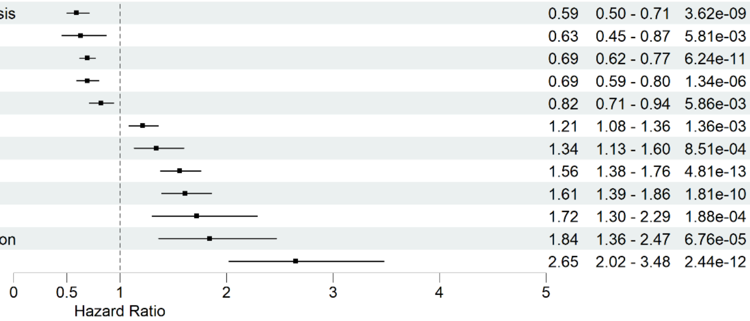

Fig. S2

Forest plot showing hazard ratios for the prior ICD-10 diagnoses that are significantly associated with the risk of Parkinson's Disease (PD). (PD ~ ICD-10 + sex + age + Townsend deprivation index).

Supplementary Figure 3A

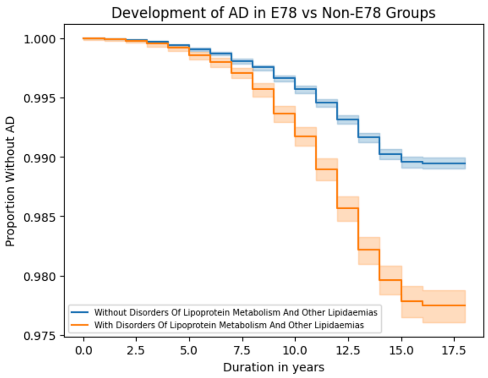

Supplementary Figure 3B

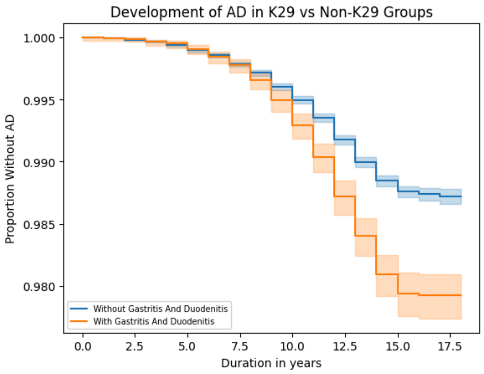

Supplementary Figure 3C

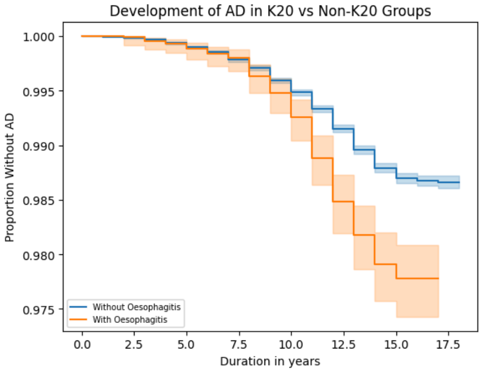

Supplementary Figure 3D

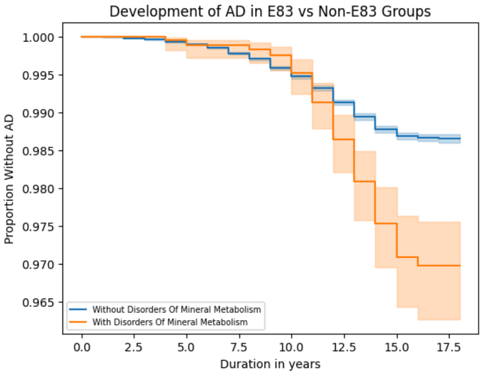

Supplementary Figure 3E

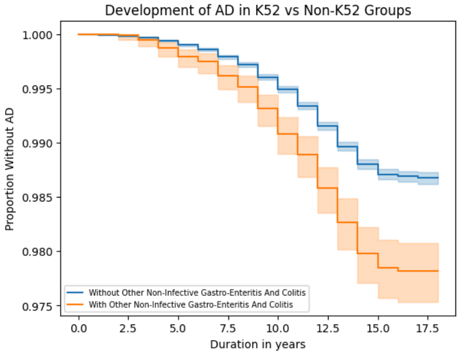

Supplementary Figure 3F

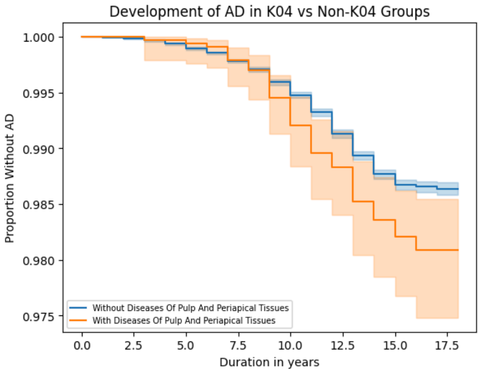

Supplementary Figure 3G

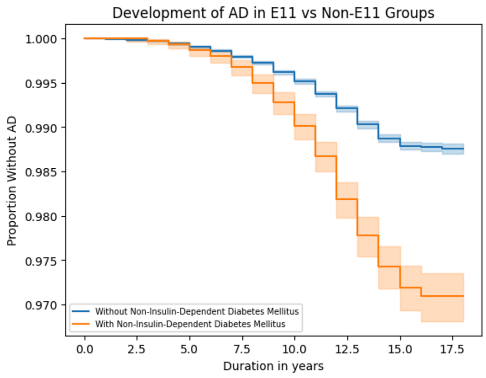

Supplementary Figure 3H

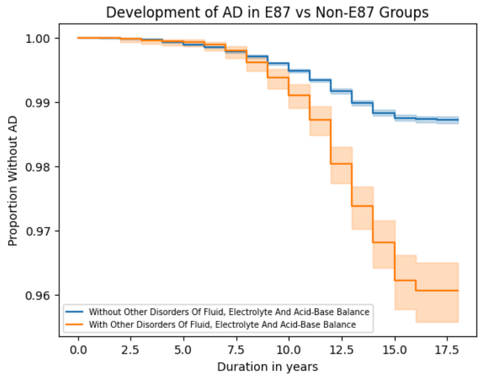

Supplementary Figure 3I

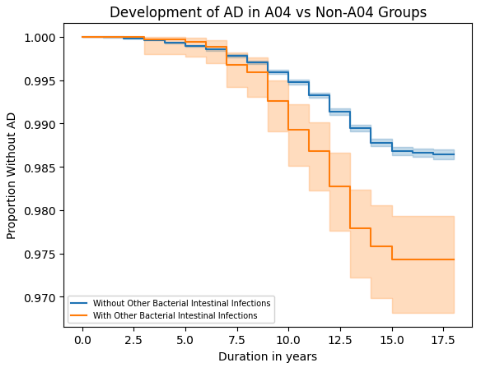

Supplementary Figure 3J

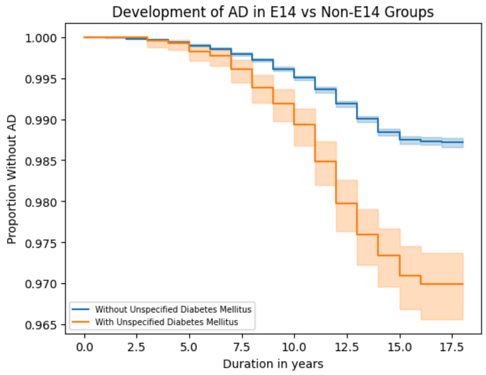

Supplementary Figure 3K

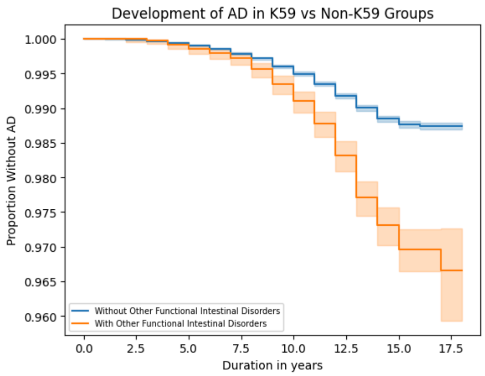

Supplementary Figure 3L

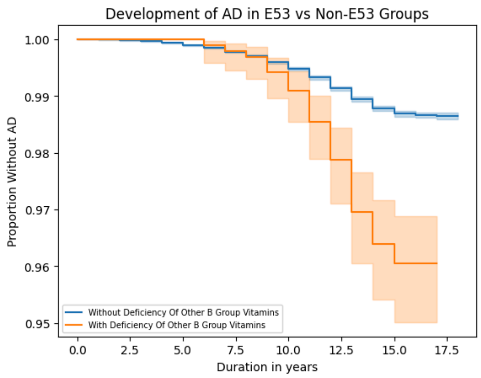

Supplementary Figure 3M

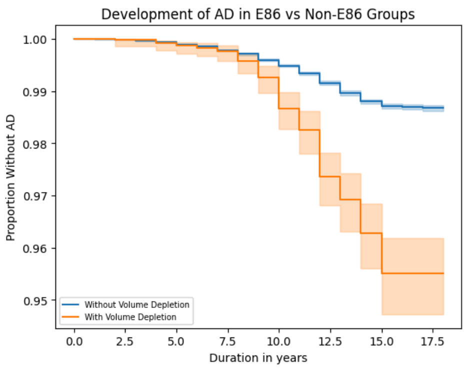

Supplementary Figure 3N

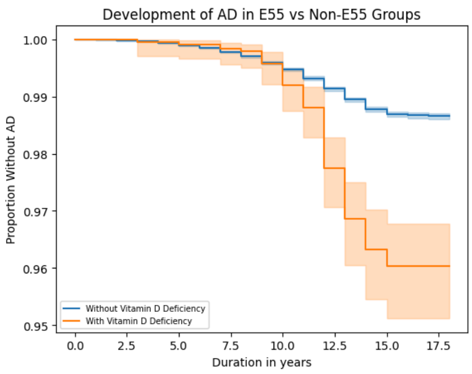

Supplementary Figure 3O

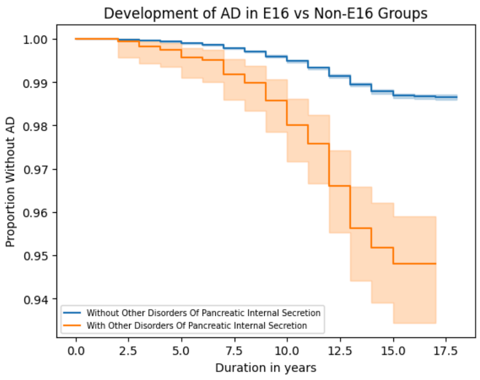

Supplementary Figure 3P

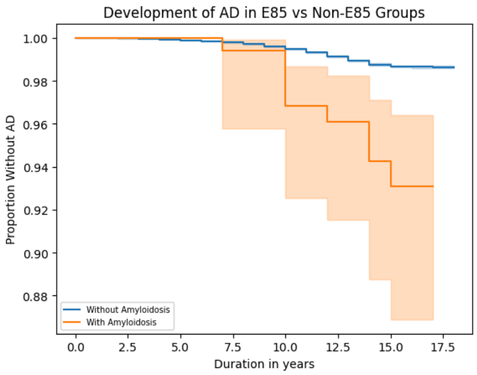

Supplementary Figure 3Q

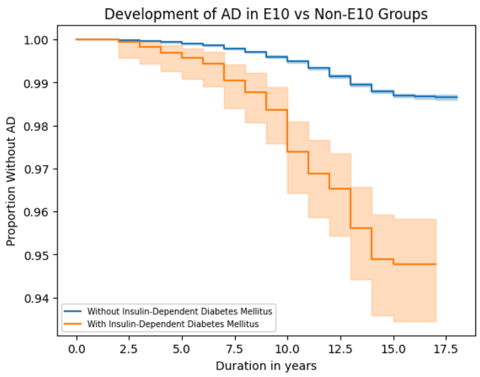

Fig. S3 Kaplan-Meier plot displaying the probabilities of developing Alzheimer's Disease for individuals categorized by specific diagnoses

Supplementary Figure 4A

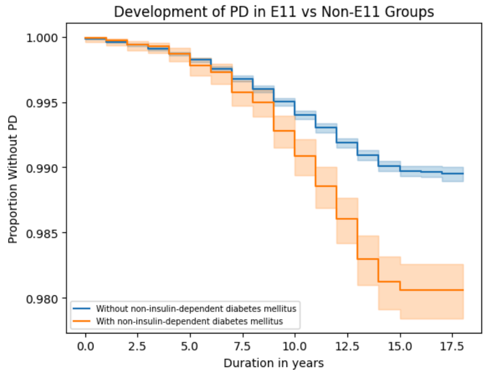

Supplementary Figure 4B

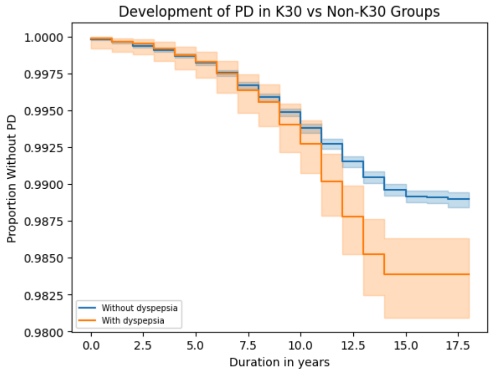

Supplementary Figure 4C

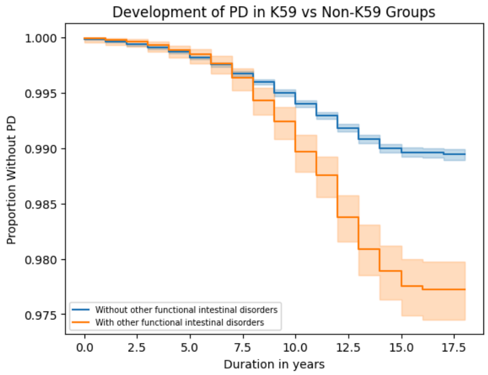

Supplementary Figure 4D

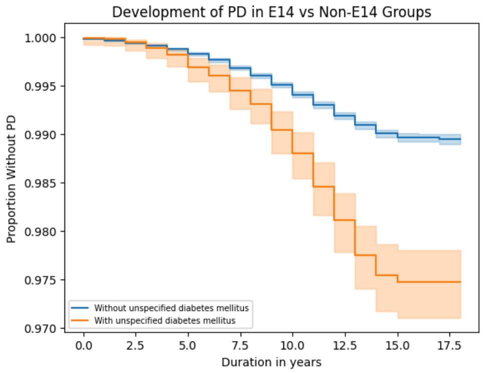

Supplementary Figure 4E

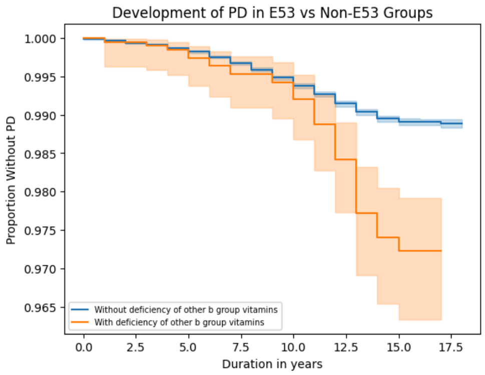

Supplementary Figure 4F

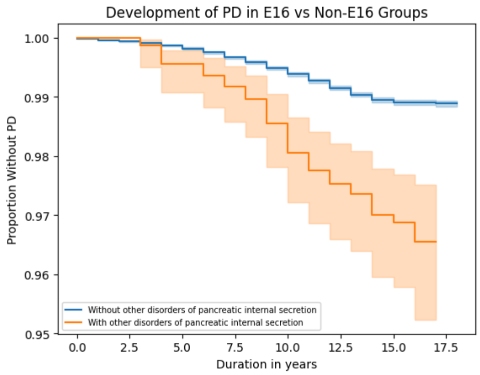

Supplementary Figure 4G

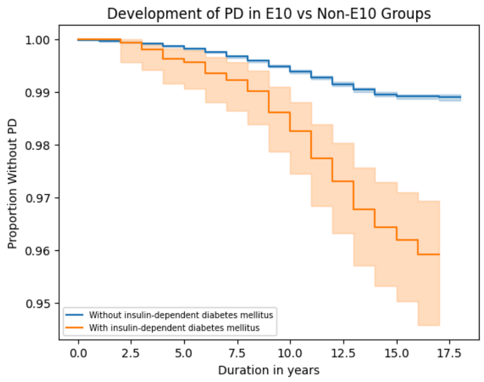

Fig. S4 Kaplan-Meier plot displaying the probabilities of developing Parkinson's Disease for individuals categorized by specific diagnoses of a disorder affecting the gut-brain axis.

Supplementary Figure 5A

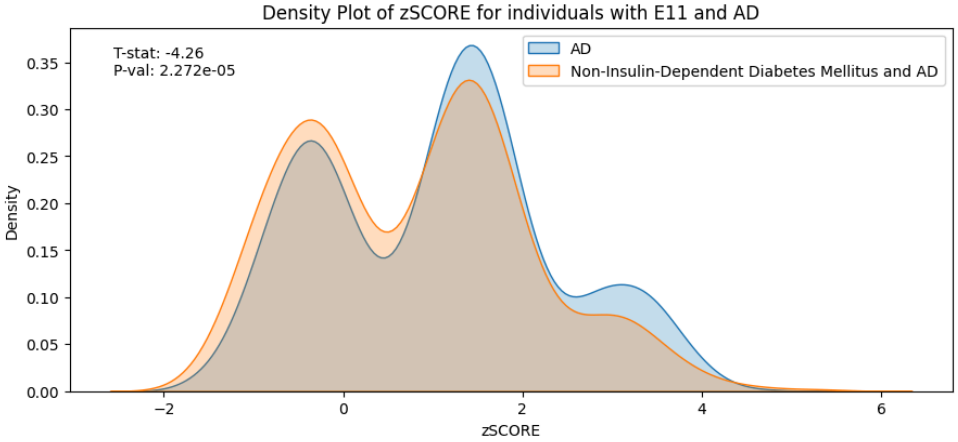

Supplementary Figure 5B

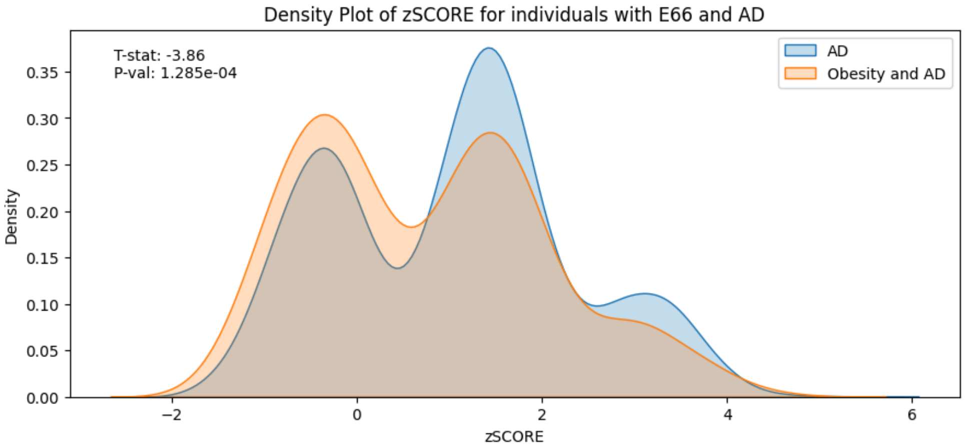

Supplementary Figure 5C

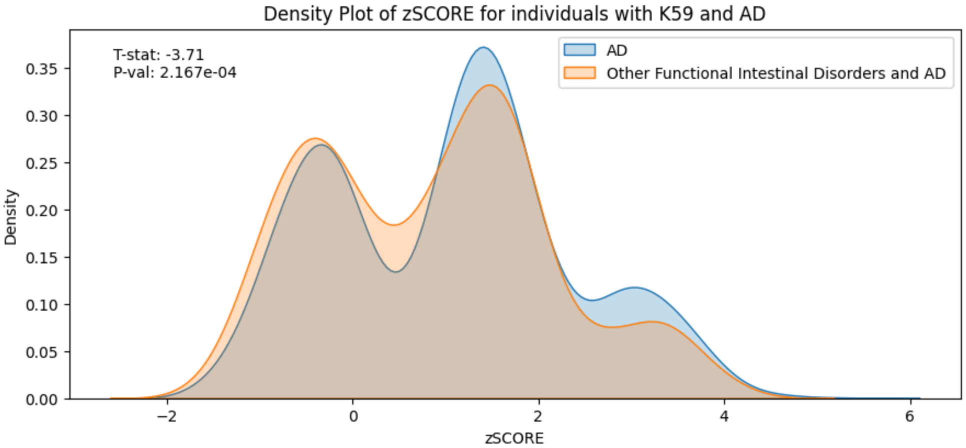

Supplementary Figure 5D

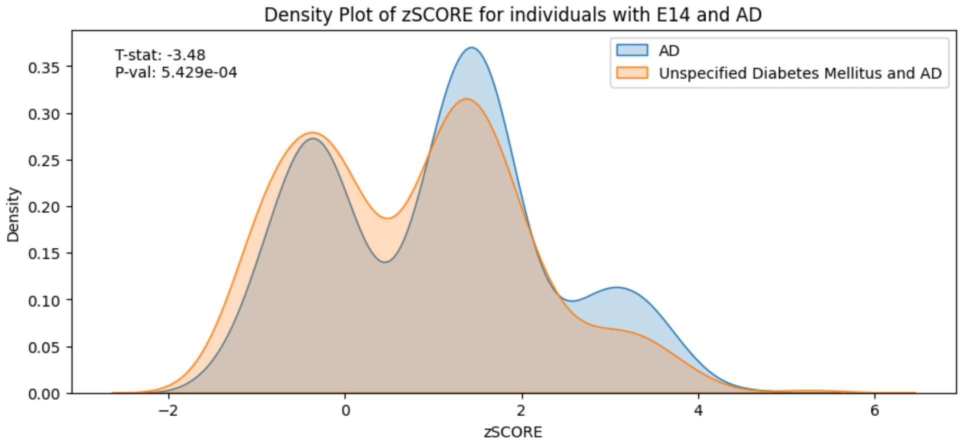

Supplementary Figure 5E

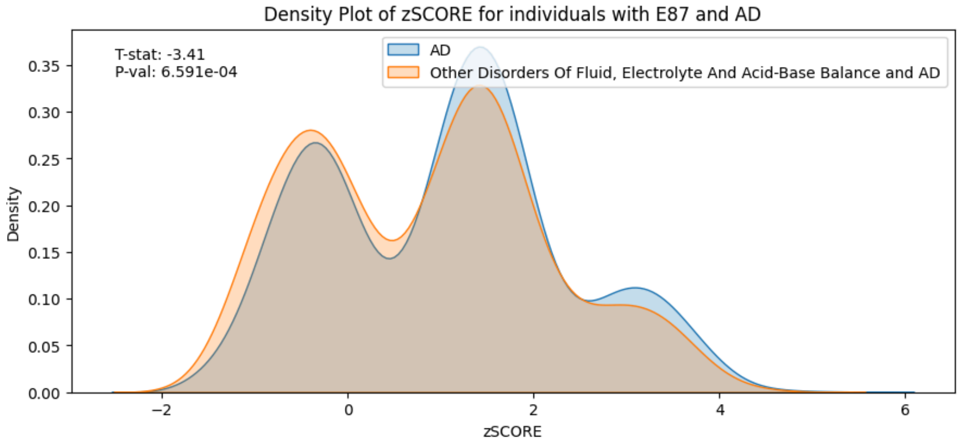

Supplementary Figure 5F

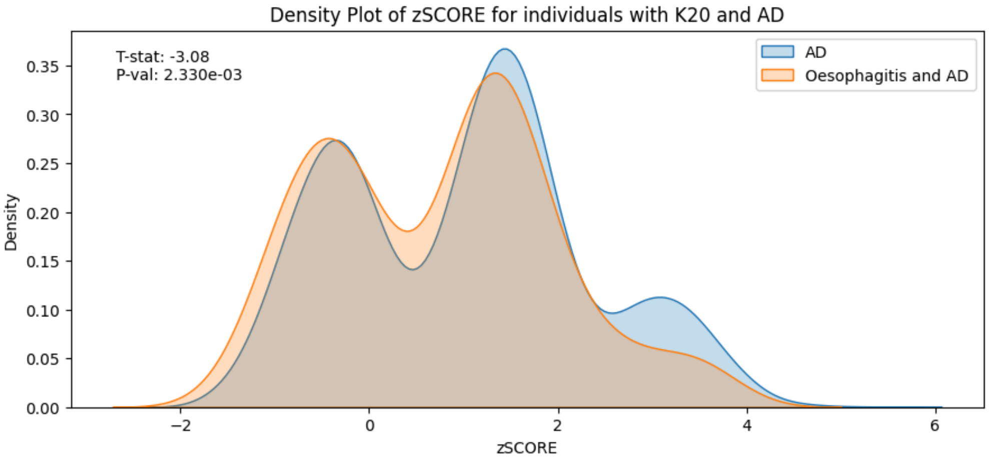

Supplementary Figure 5G

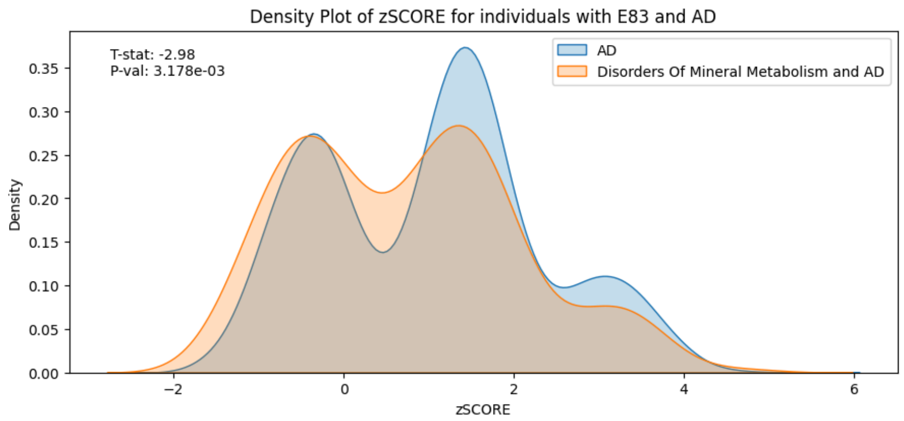

Supplementary Figure 5H

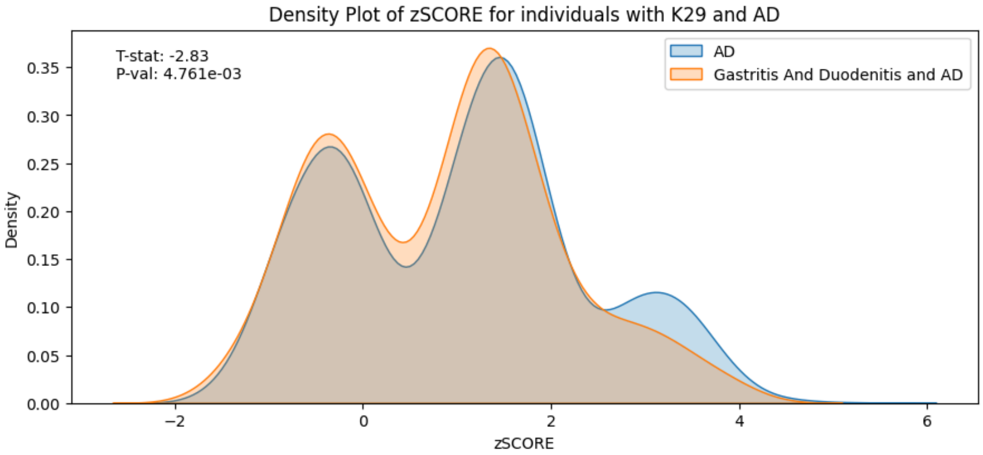

Supplementary Figure 5I

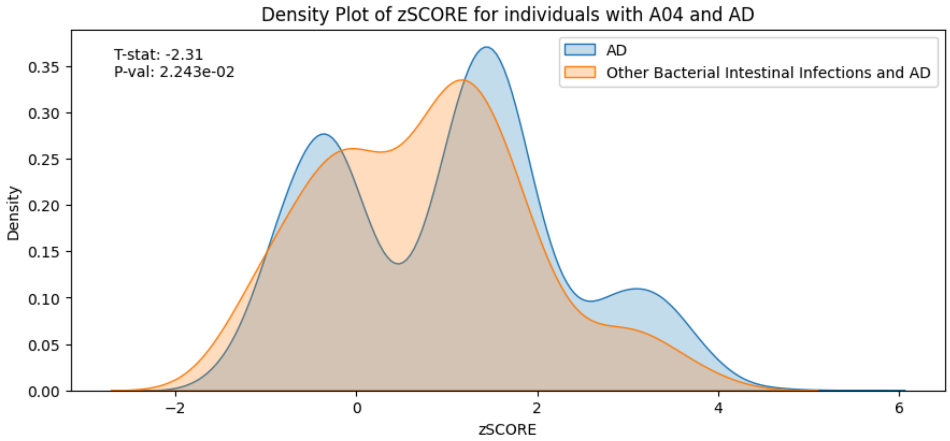

Supplementary Figure 5J

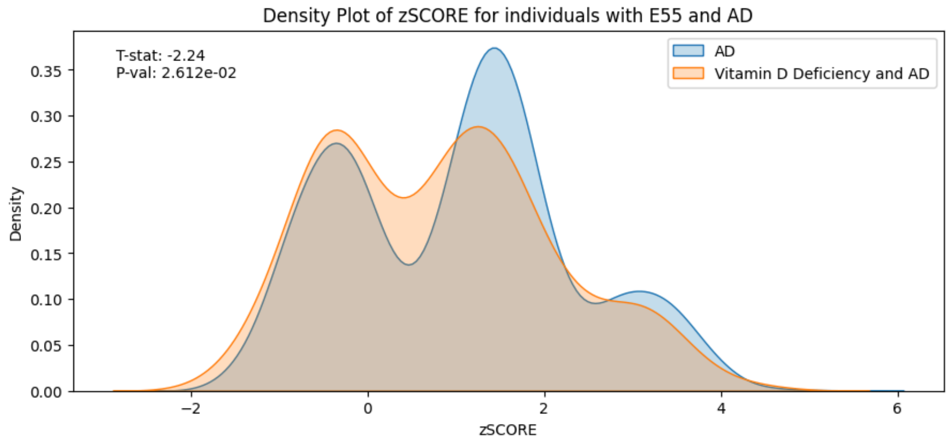

Supplementary Figure 5K

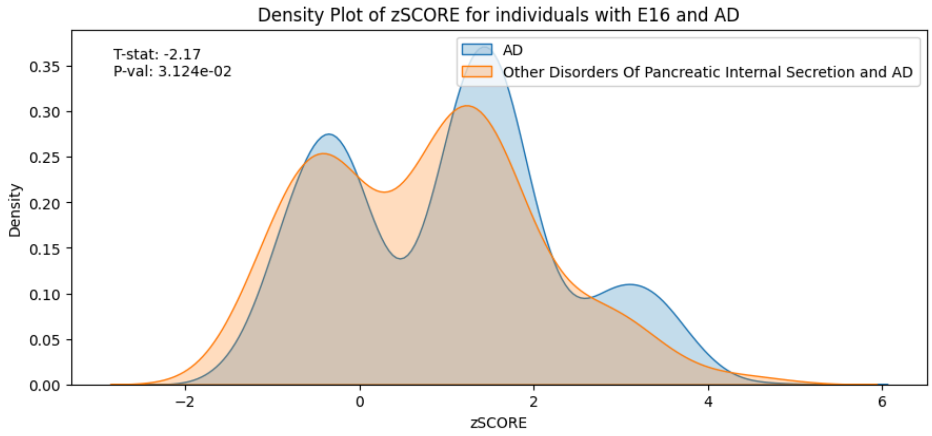

Supplementary Figure 5L

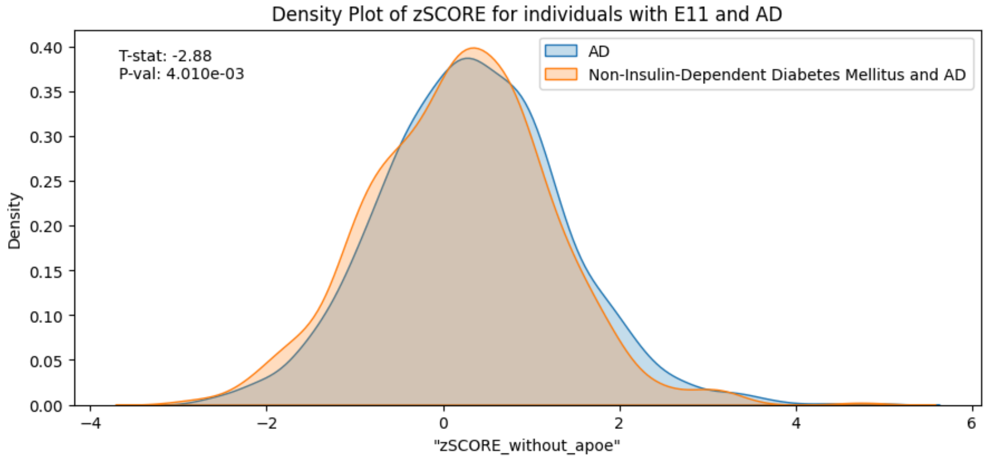

Supplementary Figure 5M

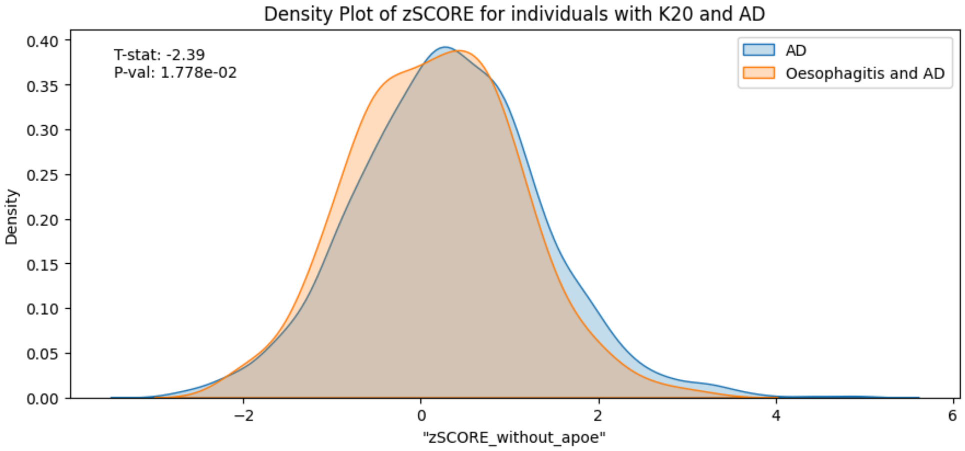

Supplementary Figure 5N

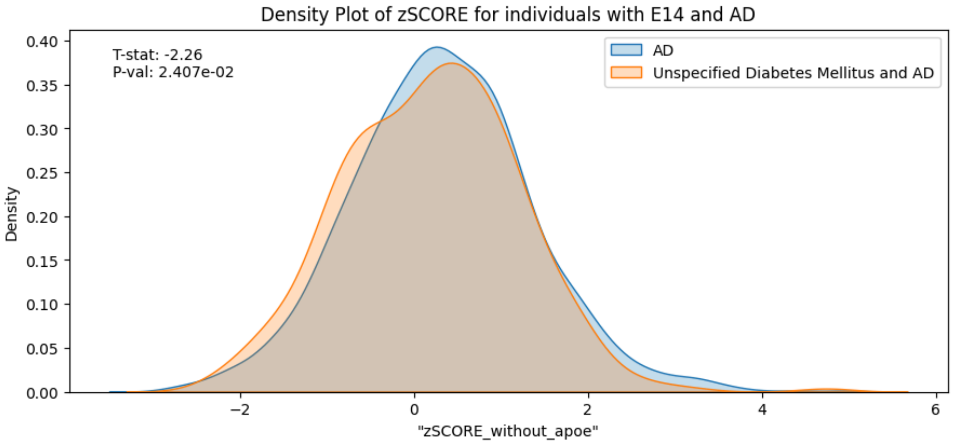

Supplementary Figure 5O

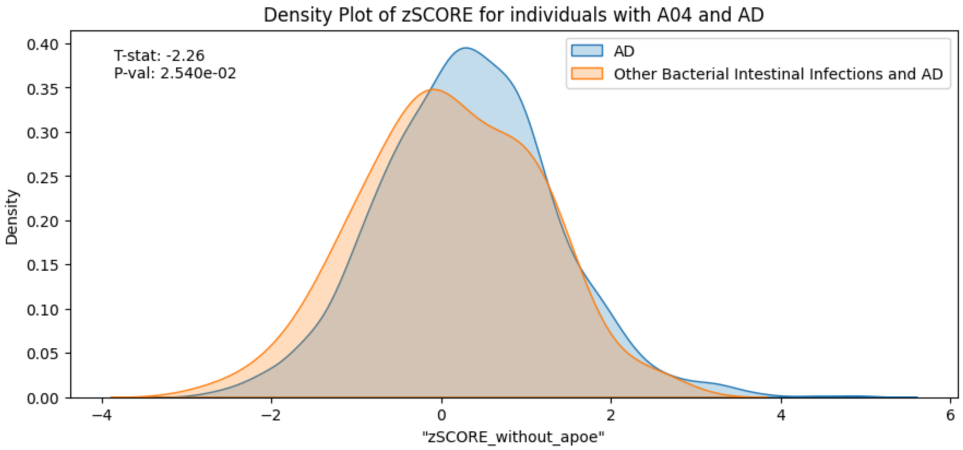

Supplementary Figure 5P

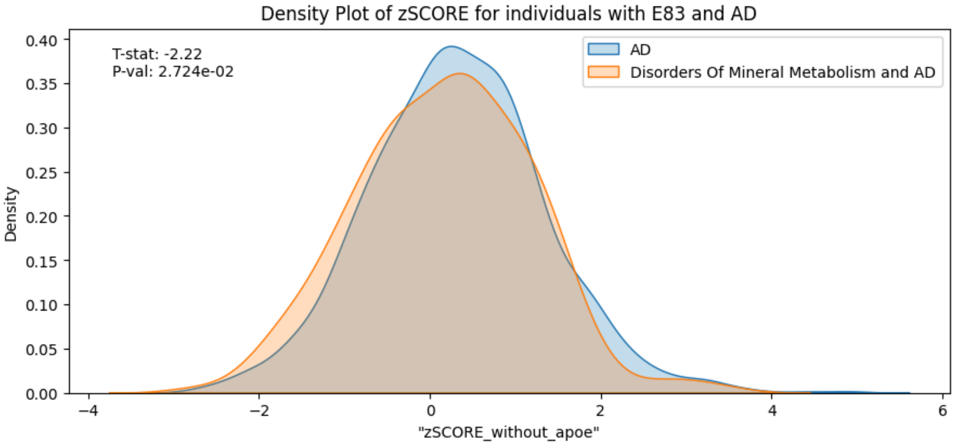

Fig. S5 Density plots of Alzheimer's Disease (AD) polygenic risk scores (PRS) with *APOE*, comparing cases with and without prior diagnoses of a disorder affecting the gut-brain axis. AD PRS is distributed differently between cases of AD with and without prior ICD-10 Diagnosis.

Supplementary Figure 6A

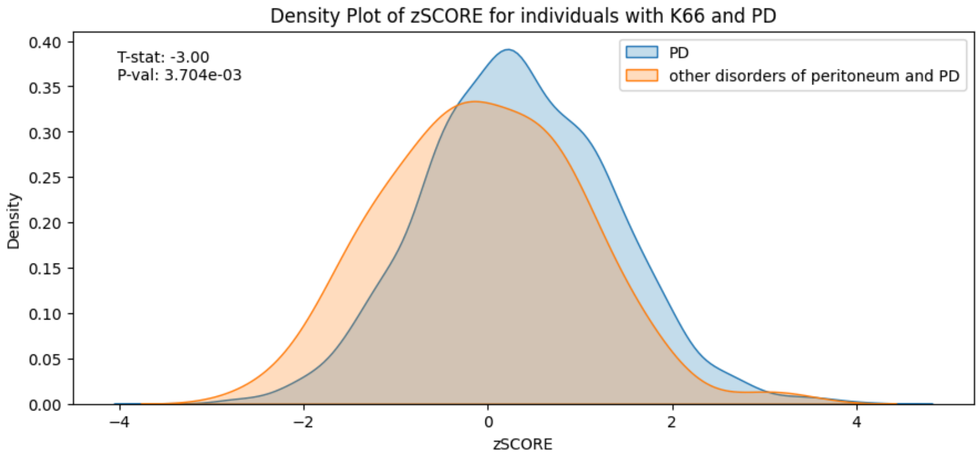

Supplementary Figure 6B

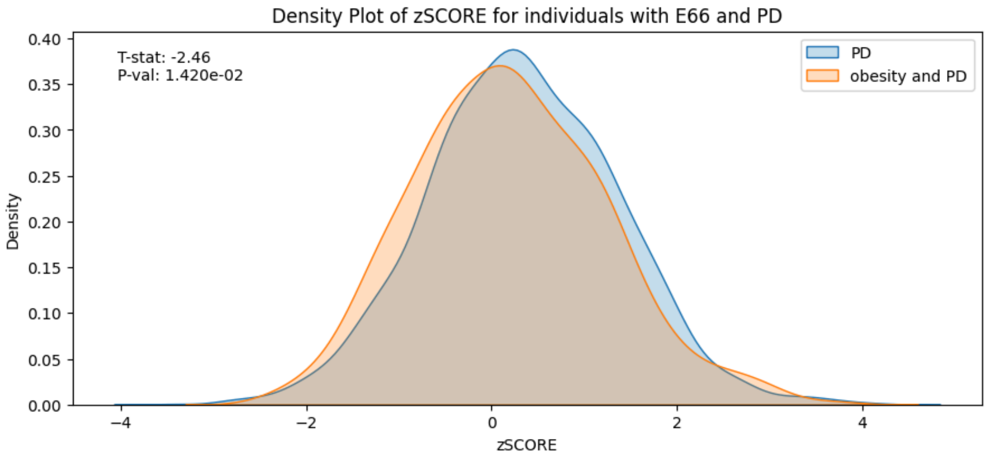

Supplementary Figure 6C

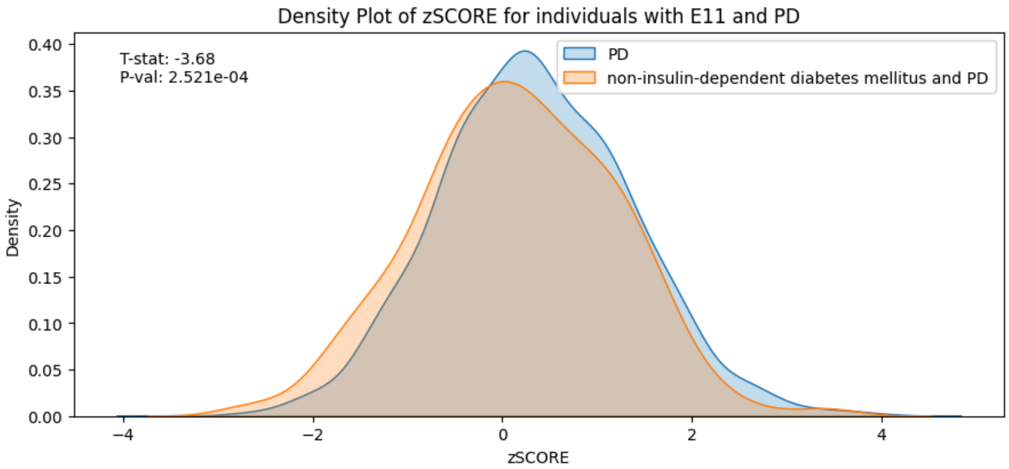

Supplementary Figure 6D

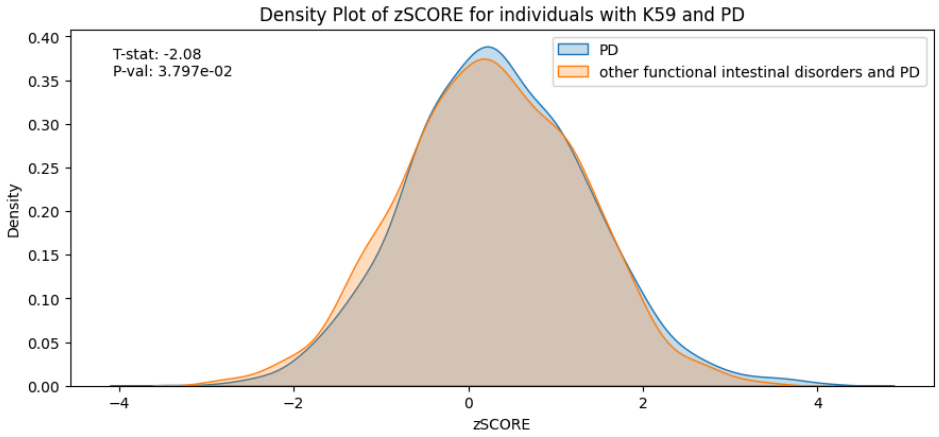

Supplementary Figure 6E

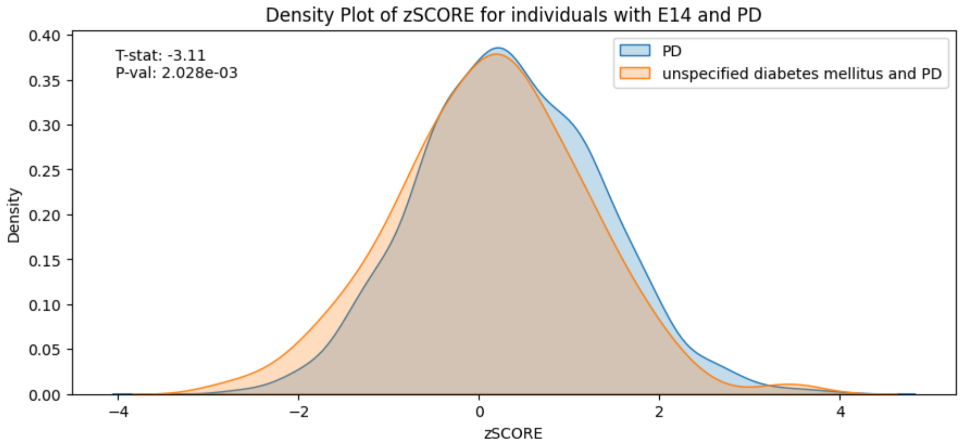

Supplementary Figure 6F

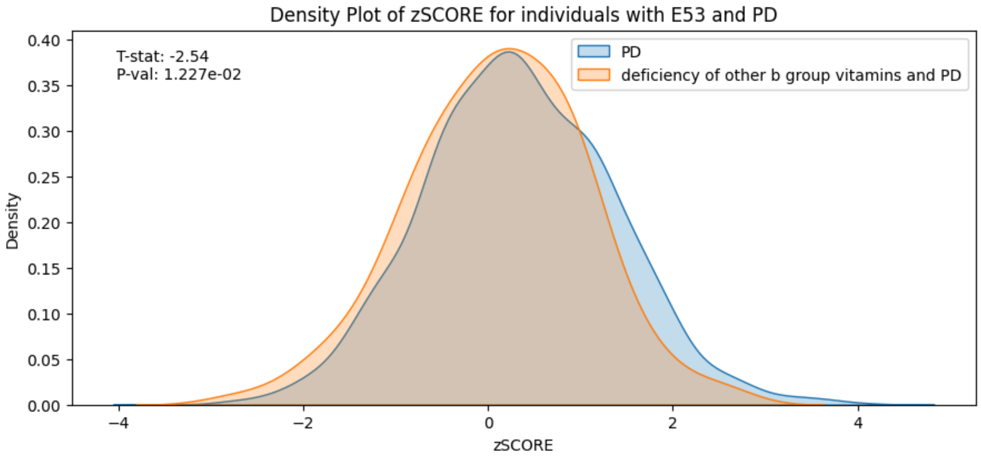

Supplementary Figure 6G

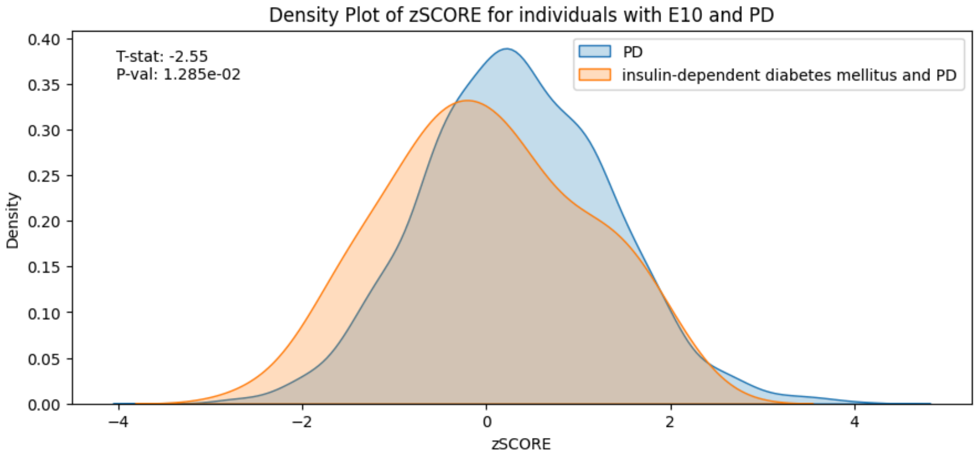

Fig. S6 Density plots of Parkinson's Disease (PD) polygenic risk scores (PRS) comparing cases with and without prior diagnosis of a disorder affecting the gut-brain axis. AD PRS is distributed differently between cases of AD with and without prior ICD-10 Diagnosis.

Figure 7A.1

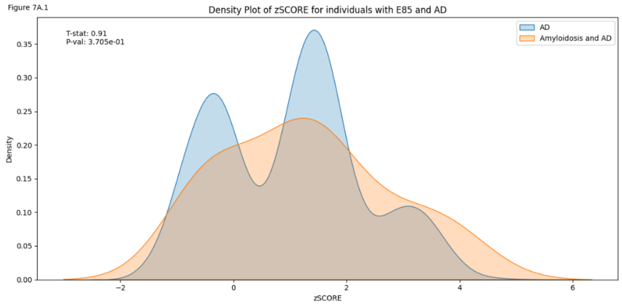

Figure 7A.2

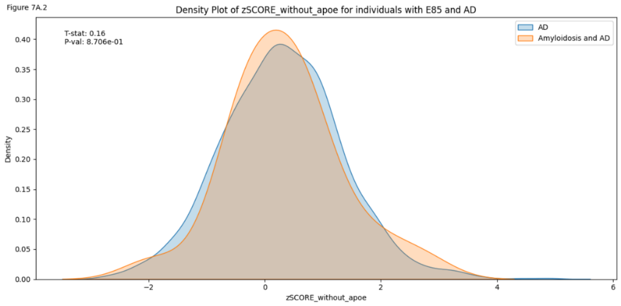

Figure 7B.1

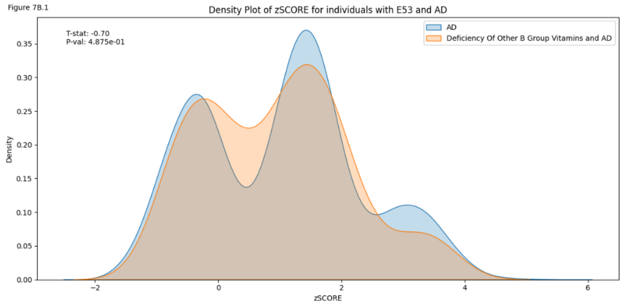

Figure 7B.2

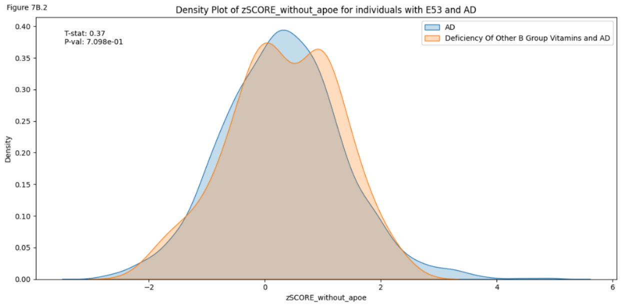

Figure 7C.1

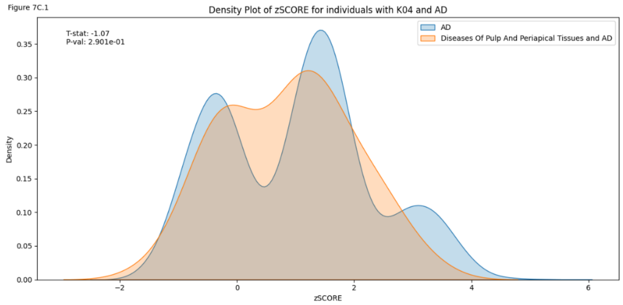

Figure 7C.2

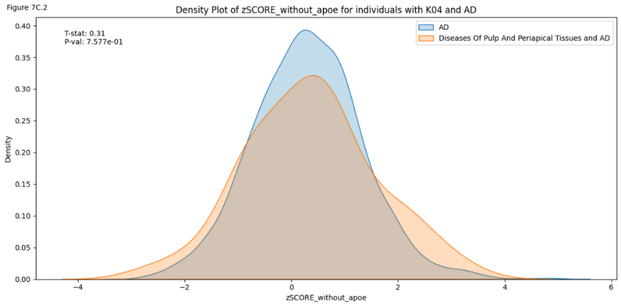

Figure 7D.1

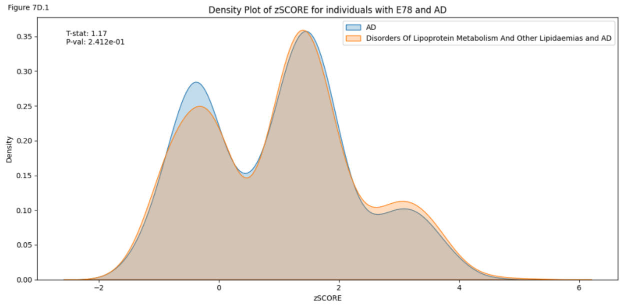

Figure 7D.2

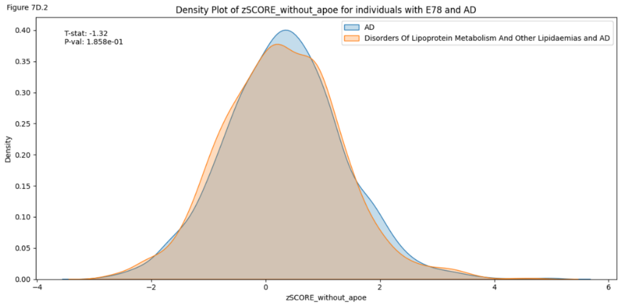

Figure 7E.1

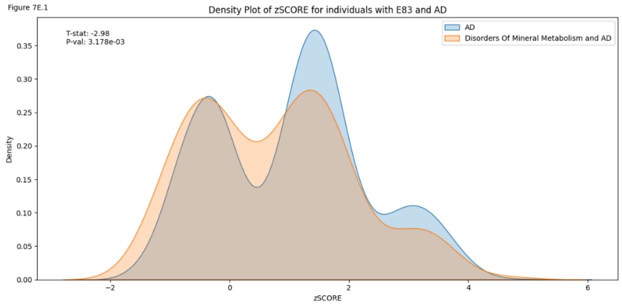

Figure 7E.2

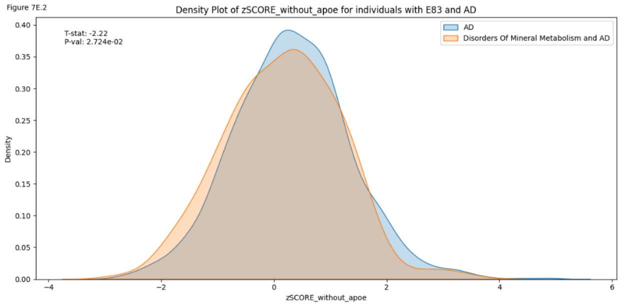

Figure 7F.1

Density Plot of zSCORE for individuals with K29 and AD

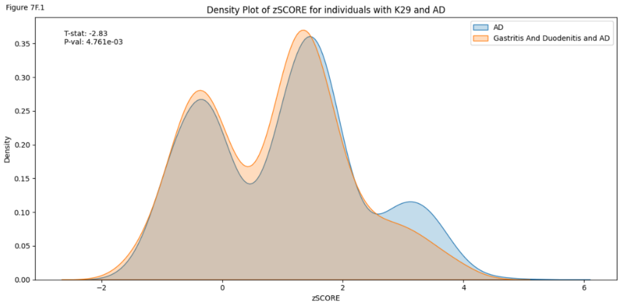

Figure 7F.2

Density Plot of zSCORE_without_apoe for individuals with K29 and AD

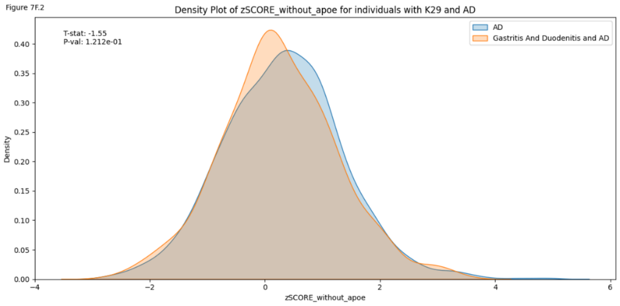

Figure 7G.1

Density Plot of zSCORE for individuals with K64 and AD

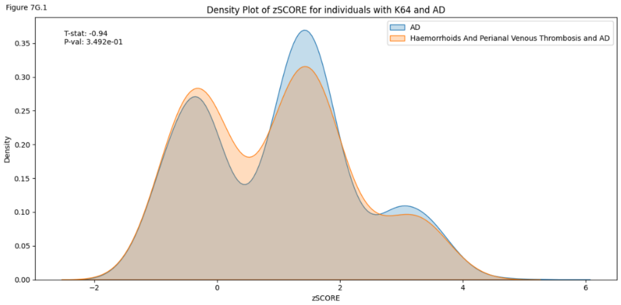

Figure 7G.2

Density Plot of zSCORE_without_apoe for individuals with K64 and AD

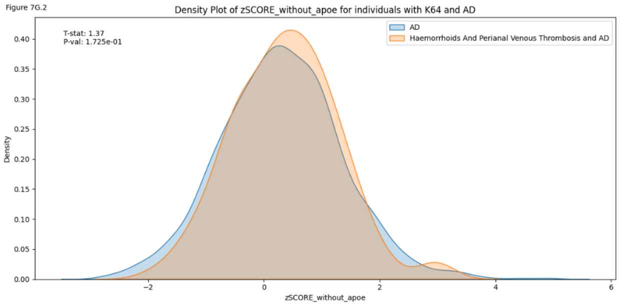

Figure 7H.1

Density Plot of zSCORE for individuals with E10 and AD

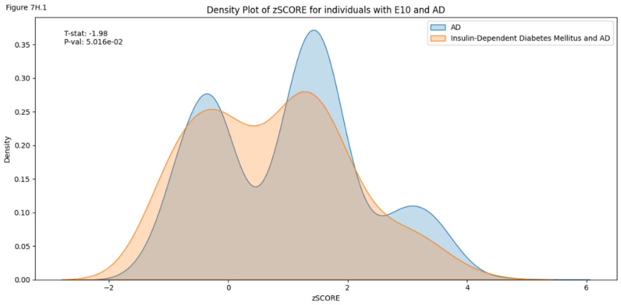

Figure 7H.2

Density Plot of zSCORE_without_apoe for individuals with E10 and AD

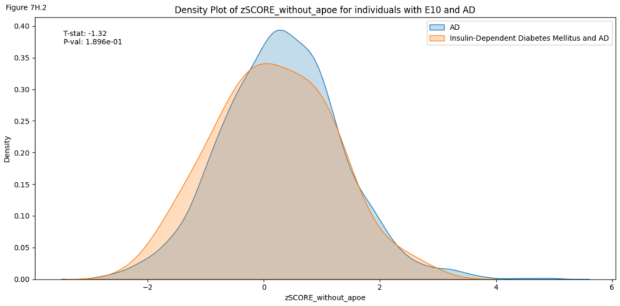

Figure 7I.1

Density Plot of zSCORE for individuals with E11 and AD

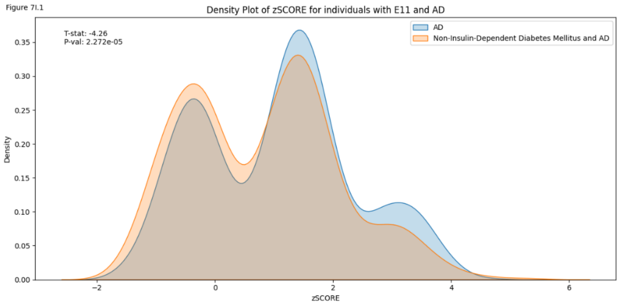

Figure 7I.2

Density Plot of zSCORE_without_apoe for individuals with E11 and AD

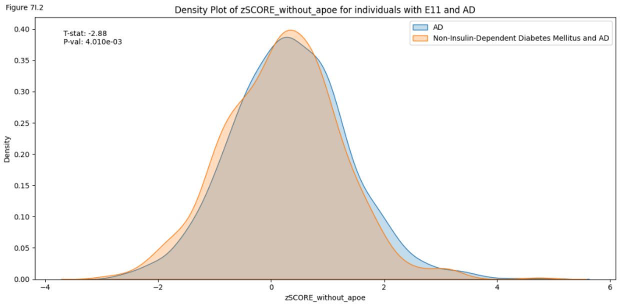

Figure 7J.1

Density Plot of zSCORE for individuals with E66 and AD

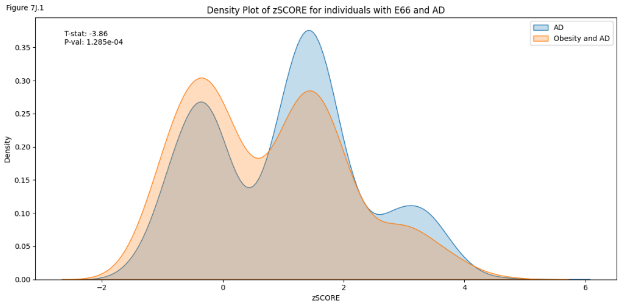

Figure 7J.2

Density Plot of zSCORE_without_apoe for individuals with E66 and AD

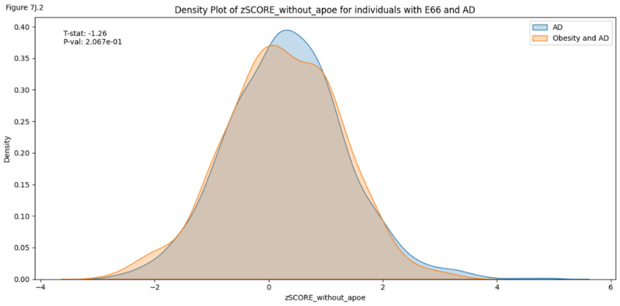

Figure 7K.1

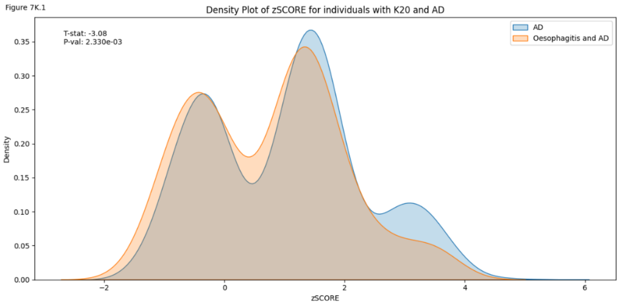

Figure 7K.2

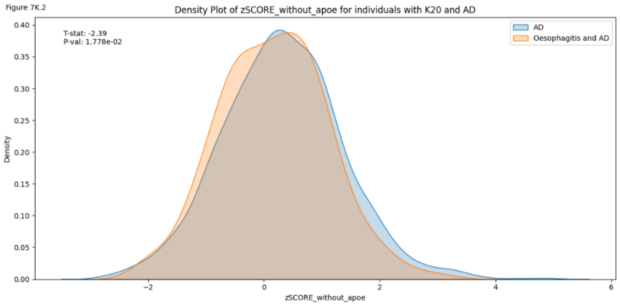

Figure 7L.1

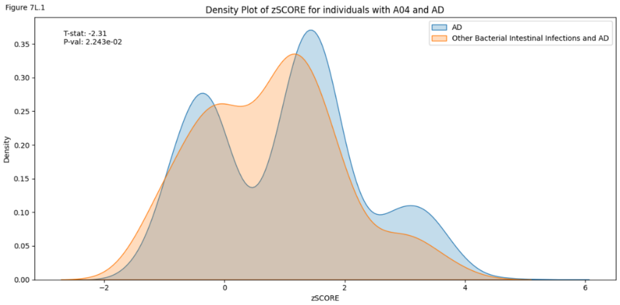

Figure 7L.2

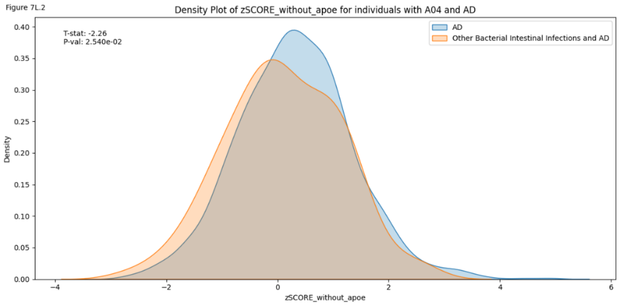

Figure 7M.1

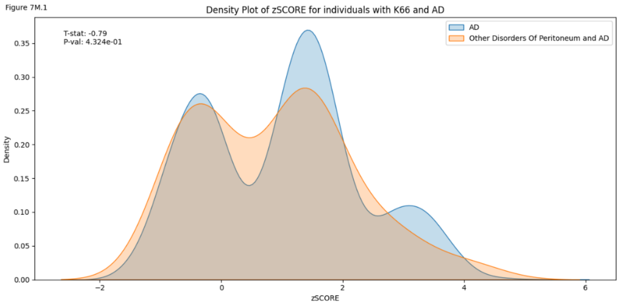

Figure 7M.2

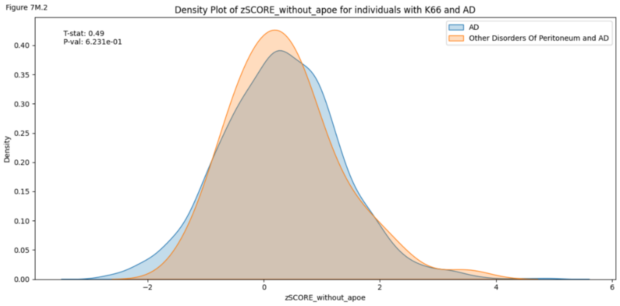

Figure 7N.1

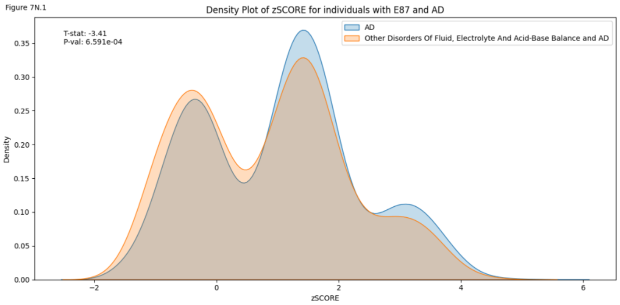

Figure 7N.2

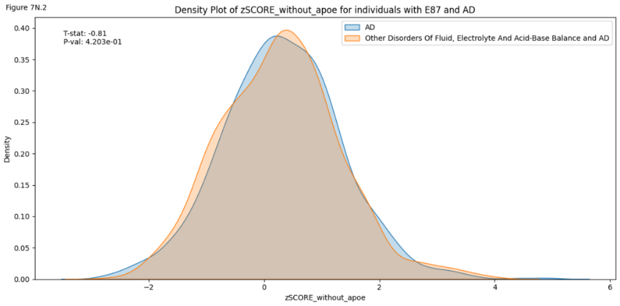

Figure 7O.1

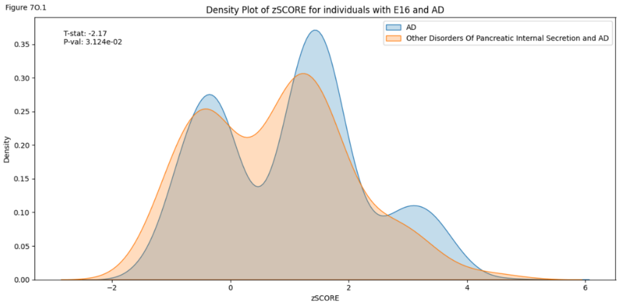

Figure 7O.2

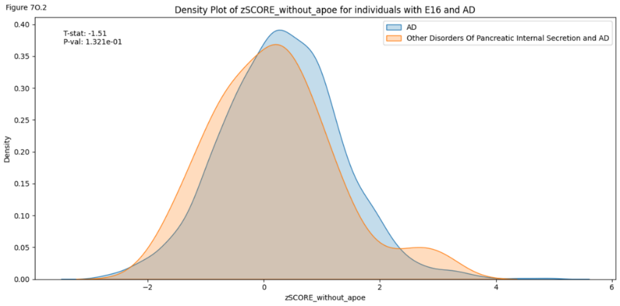

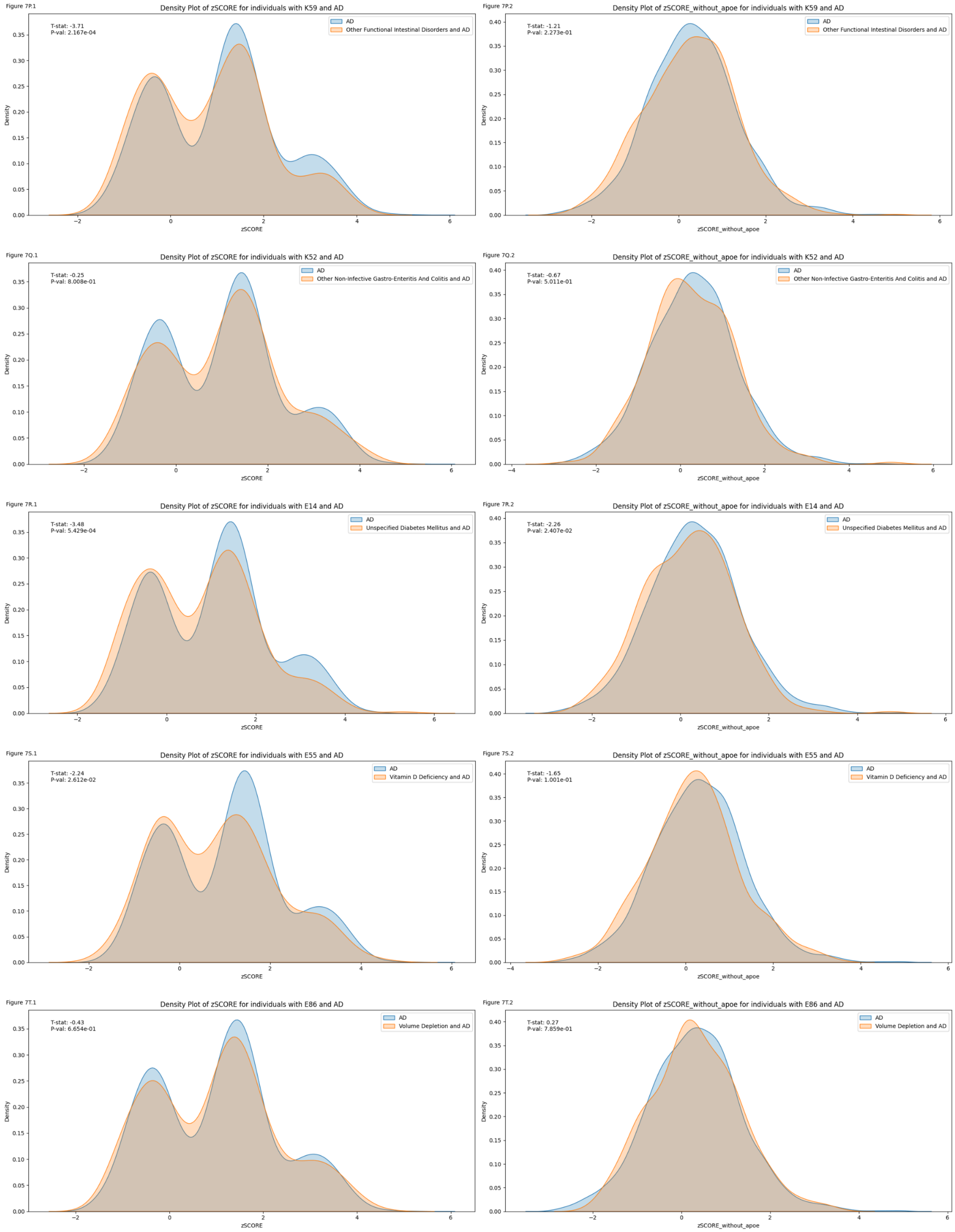

Fig. S7 Alzheimer's Disease (AD) polygenic risk scores (PRS) with and without *APOE* are differently distributed between cases of AD with and without prior diagnosis of a disorder

affecting the gut-brain axis.

Supplementary Figure 8A

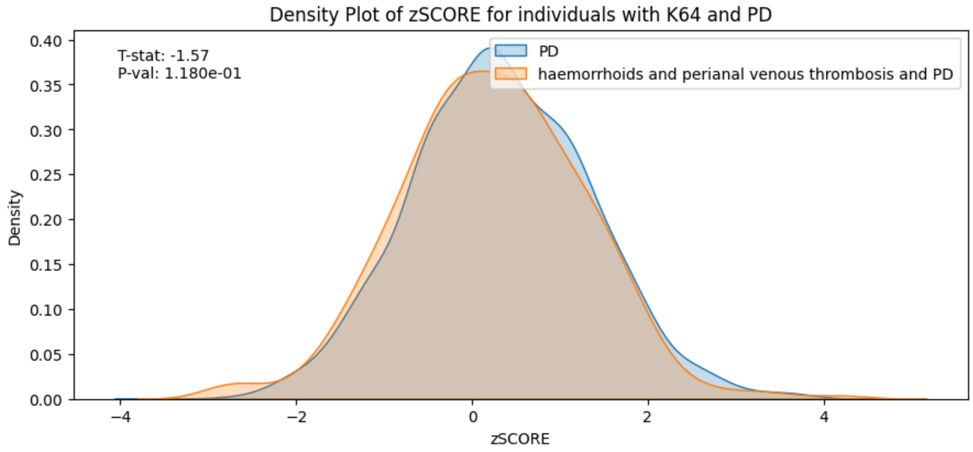

Supplementary Figure 8B

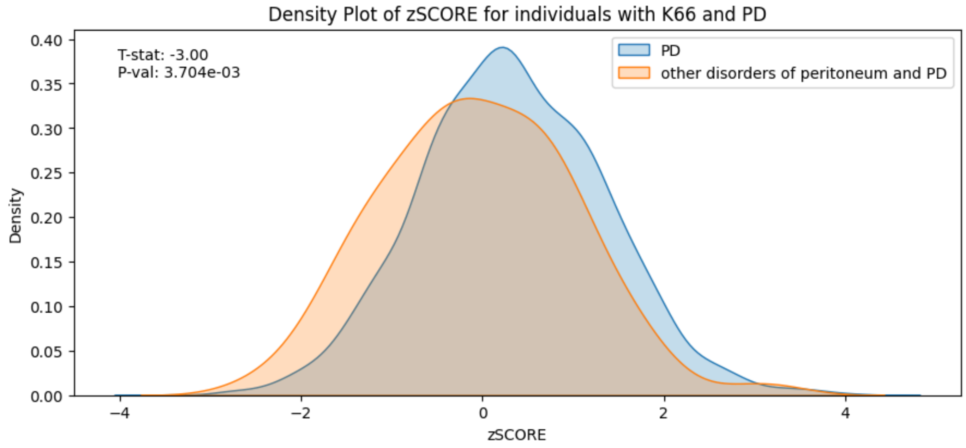

Supplementary Figure 8C

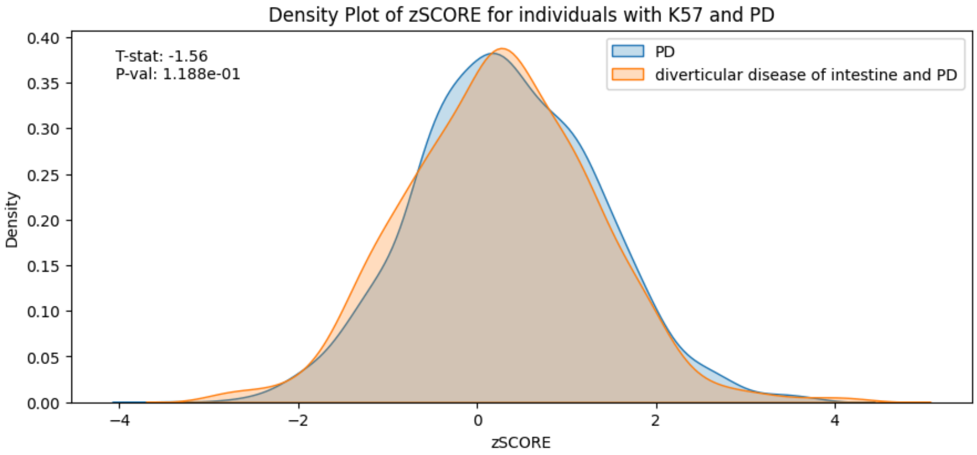

Supplementary Figure 8D

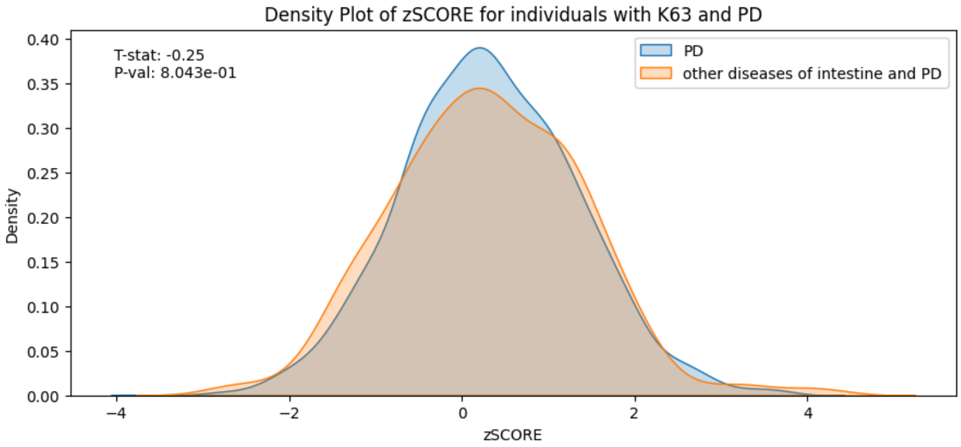

Supplementary Figure 8E

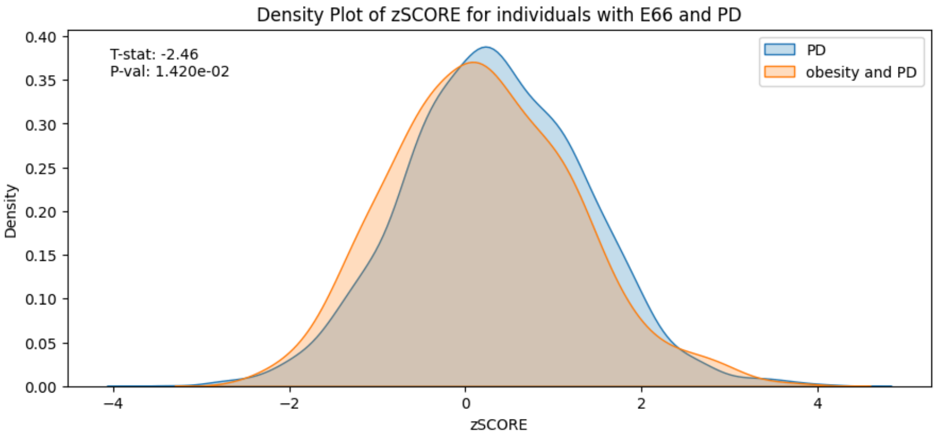

Supplementary Figure 8F

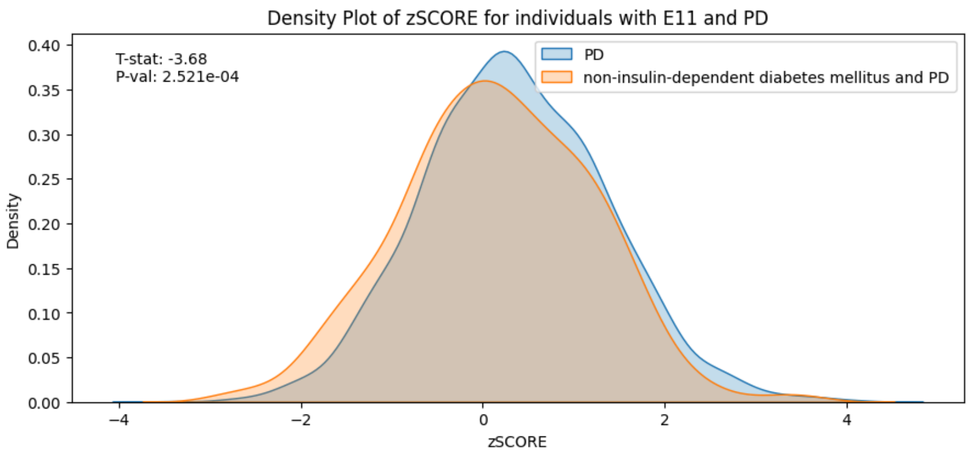

Supplementary Figure 8G

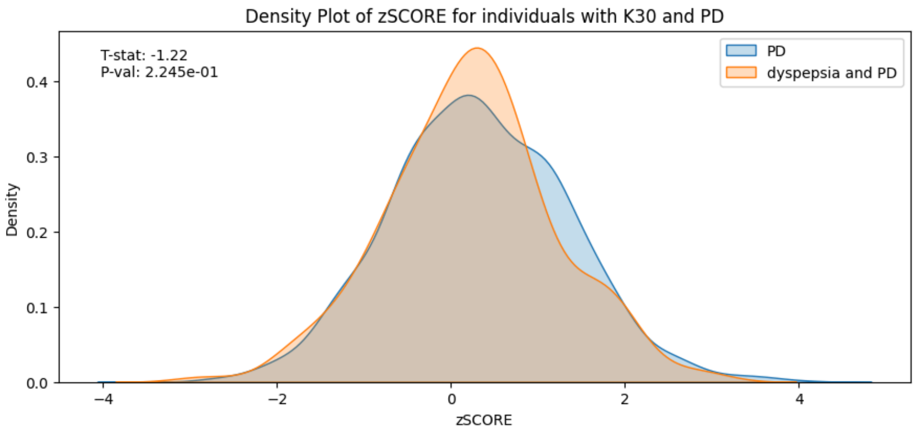

Supplementary Figure 8H

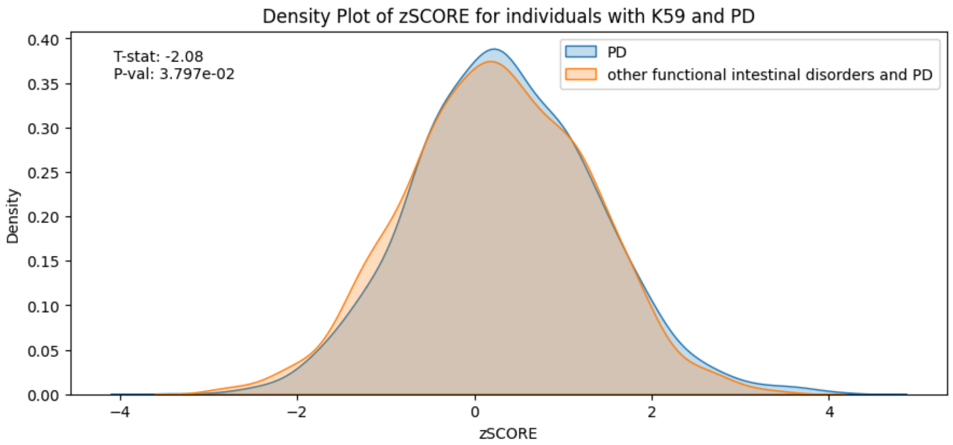

Supplementary Figure 8I

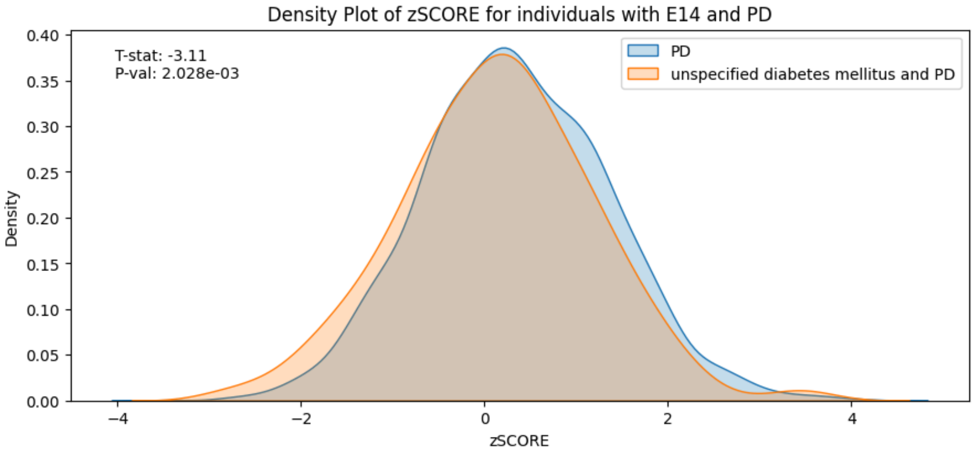

Supplementary Figure 8J

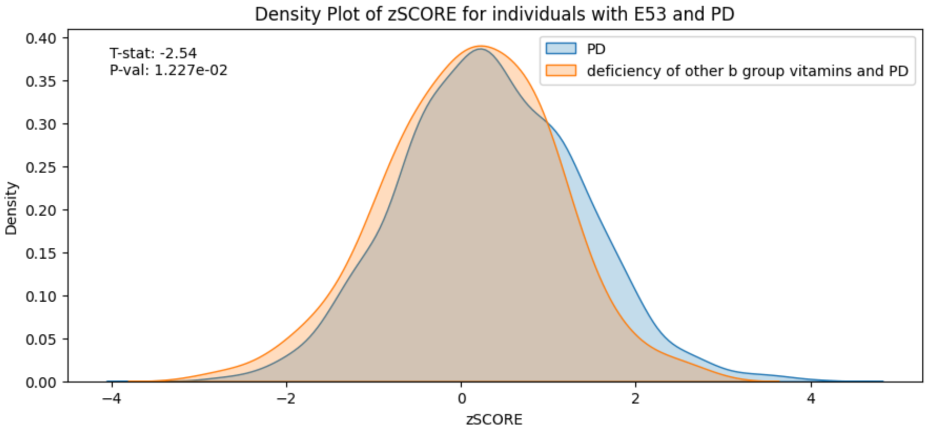

Supplementary Figure 8K

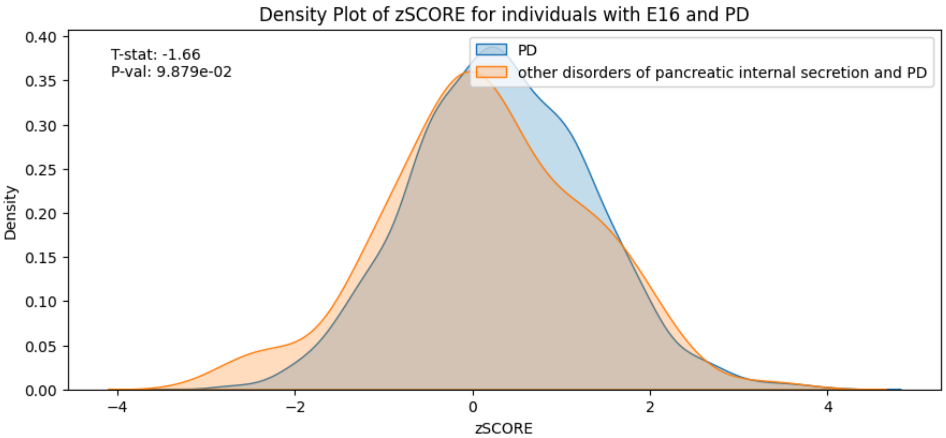

Supplementary Figure 8L

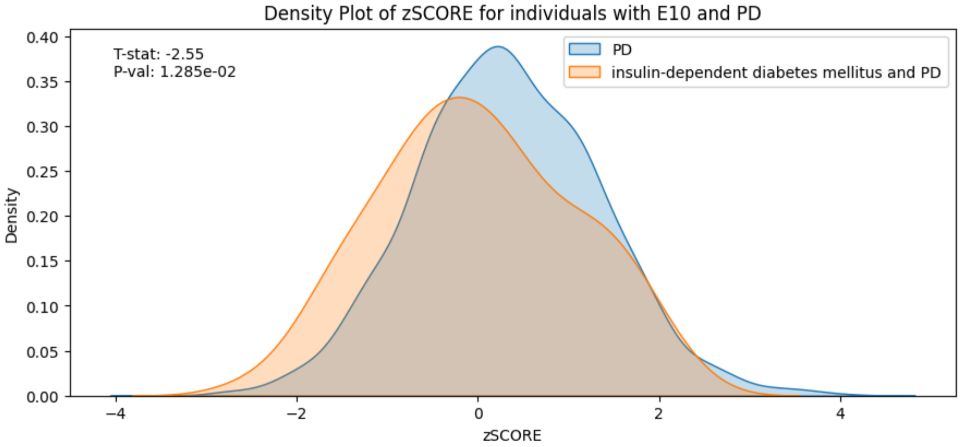

Fig. S8 Parkinson's Disease (PD) polygenic risk scores (PRS) are differently distributed between cases of PDs with and without prior diagnosis of a disorder affecting the gut-brain axis.
