# Supplementary material for: Gut-Brain Nexus: Mapping Multi-Modal Links to Neurodegeneration at Biobank Scale
Source: medRxiv. 2024 Nov 6:2024.09.12.24313490. Originally published 2024 Sep 13. Preprint. [Version 2] doi: 10.1101/2024.09.12.24313490 (PMC11451806; doi:10.1101/2024.09.12.24313490)
Supplement: Supplement 2 [file media-2.pdf]

Overview supplementary tables

Supplementary Table 1. Cox proportional hazards regression analysis of Alzheimer's disease and endocrine, nutritional, metabolic, and digestive system disorders ICD-10 codes adjusted for year of birth, Townsend deprivation index, and sex

Supplementary Table 2. Cox proportional hazards regression analysis of Parkinson's disease and endocrine, nutritional, metabolic, and digestive system disorders ICD-10 codes adjusted for year of birth, Townsend deprivation index, and sex

Supplementary Table 3. Interaction terms between endocrine, nutritional, metabolic, digestive system disorders and Alzheimer’s disease polygenic risk score (excluding APOE)

Supplementary Table 4. Interaction terms between endocrine, nutritional, metabolic, and digestive system disorders and Alzheimer’s disease polygenic risk score

Supplementary Table 5. Interaction terms between endocrine, nutritional, metabolic, digestive system disorders and Parkinson’s disease polygenic risk score

Supplementary Table 6. Biomarkers Associated with AD

Supplementary Table 7. Biomarkers Associated with PD

Supplementary Table 8. Performance summary of different feature sets for AD classification

Supplementary Table 9. Performance summary of different feature sets for PD classification

Supplementary Table 10. ICD 10 codes used to create control cohort

Supplementary Table 11. Biobank cohort gender and Alzheimer’s disease/Parkinson’s disease demographics

Supplementary Table 12. UKB diagnosis and disorders under study

Supplementary Table 13. Proteomic biomarkers under study

Supplementary Table 14. Cox proportional hazards regression analysis of Alzheimer’s disease and endocrine, nutritional, metabolic, and digestive system disorders ICD-10 codes adjusted for APOE4/4 status, principal components 1-5, year of birth, Townsend deprivation index, and sex

Supplementary Table 15. Cox proportional hazards regression analysis of Alzheimer's disease and endocrine, nutritional, metabolic, and digestive system disorders ICD-10 codes adjusted for polygenic risk Z-score excluding APOE, APOE4/4 status, principal components 1-5, year of birth, Townsend deprivation index, and sex

Supplementary Table 16. Cox proportional hazards regression analysis of Alzheimer's disease and endocrine, nutritional, metabolic, and digestive system disorders ICD-10 codes adjusted for polygenic risk Z-scores excluding APOE, principal components 1-5, year of birth, Townsend deprivation index, and sex

Supplementary Table 17. Cox proportional hazards regression analysis of Alzheimer's disease and endocrine, nutritional, metabolic, and digestive system disorders ICD-10 codes adjusted for polygenic risk Z-score, APOE4/4 status, principal components 1-5, year of birth, Townsend deprivation index, and sex

Supplementary Table 18. Cox proportional hazards regression analysis of Alzheimer's disease and endocrine, nutritional, metabolic, and digestive system disorders ICD-10 codes adjusted for polygenic risk Z-scores, principal components 1-5, year of birth, Townsend deprivation index, and sex

Supplementary Table 19. Cox proportional hazards regression analysis of Alzheimer's disease and endocrine, nutritional, metabolic, and digestive system disorders ICD-10 codes adjusted for principal components 1-5, year of birth, Townsend deprivation index, and sex

Supplementary Table 20. Cox proportional hazards regression analysis of Parkinson's disease and endocrine, nutritional, metabolic, and digestive system disorders ICD-10 codes adjusted for principal components 1-5, year of birth, Townsend deprivation index, and sex

Supplementary Table 21. Cox proportional hazards regression analysis of Parkinson's disease and endocrine, nutritional, metabolic, and digestive system disorders ICD-10 codes adjusted for polygenic risk Z-scores, principal components 1-5, Townsend deprivation index, and sex

Supplementary Table 22. Cox proportional hazards regression analysis of Parkinson's disease and endocrine, nutritional, metabolic, and digestive system disorders ICD-10 codes adjusted for GBA1 (G_1_155162560 + T_1_155235843) status, principal components 1-5, year of birth, Townsend deprivation index, and sex

Supplementary Table 23. Cox proportional hazards regression analysis of Parkinson's disease and endocrine, nutritional, metabolic, and digestive system disorders ICD-10 codes adjusted for polygenic risk Z-scores, GBA1 (G_1_155162560 + T_1_155235843) status, principal components 1-5, year of birth, Townsend deprivation index, and sex

Supplementary Table 24. Cox proportional hazards regression analysis of Parkinson's disease and endocrine, nutritional, metabolic, and digestive system disorders ICD-10 codes adjusted for LRRK2 (C_12_40220632 + G_12_40340400) status, principal components 1-5, year of birth, Townsend deprivation index, and sex

Supplementary Table 25. Cox proportional hazards regression analysis of Parkinson's disease and endocrine, nutritional, metabolic, and digestive system disorders ICD-10 codes adjusted for polygenic risk Z-scores, LRRK2 (C_12_40220632 + G_12_40340400) status, principal components 1-5, year of birth, Townsend deprivation index, and sex

Cox proportional hazards regression analysis of Alzheimer's disease and endocrine, nutritional, metabolic, and digestive system disorders ICD-10 codes adjusted for year of birth, Townsend deprivation index, and sex

UKB field corresponding to the ICD 10 code	ICD10 code	Definition of ICD10 code	Hazard Ratio	ci_min	ci_max	P_VAL	N_pairs	n	P_VAL_FDR_CORRECTED	rejected
p131654	K66	other disorders of peritoneum	0.59	0.44	0.81	8.77E-04	41	4636	5.76E-03	TRUE
p131650	K64	haemorrhoids and perianal venous thrombosis	0.69	0.59	0.80	6.94E-07	186	20162	8.10E-06	TRUE
p130792	E66	obesity	0.85	0.74	0.96	9.29E-03	260	22619	4.43E-02	TRUE
p130814	E78	disorders of lipoprotein metabolism and other lipidaemias	1.18	1.09	1.27	1.53E-05	1090	53797	1.34E-04	TRUE
p131598	K29	gastritis and duodenitis	1.20	1.09	1.32	1.45E-04	523	27914	1.17E-03	TRUE
p131582	K20	oesophagitis	1.28	1.10	1.49	1.63E-03	175	8660	1.00E-02	TRUE
p130820	E83	disorders of mineral metabolism	1.32	1.08	1.62	7.75E-03	94	4435	3.88E-02	TRUE
p131630	K52	other non-infective gastro-enteritis and colitis	1.37	1.20	1.56	1.75E-06	250	12984	1.84E-05	TRUE
p131560	K04	diseases of pulp and periapical tissues	1.44	1.11	1.87	6.21E-03	57	3365	3.26E-02	TRUE
p130708	E11	non-insulin-dependent diabetes mellitus	1.47	1.33	1.63	1.19E-14	476	19048	4.18E-13	TRUE
p130828	E87	other disorders of fluid, electrolyte and acid-base balance	1.51	1.34	1.70	6.43E-12	312	11313	1.35E-10	TRUE
p130008	A04	other bacterial intestinal infections	1.51	1.21	1.89	2.35E-04	81	3585	1.76E-03	TRUE
p130714	E14	unspecified diabetes mellitus	1.53	1.33	1.75	1.21E-09	224	8733	1.58E-08	TRUE
p131640	K59	other functional intestinal disorders	1.55	1.40	1.73	3.55E-16	394	15596	1.86E-14	TRUE
p130770	E53	deficiency of other b group vitamins	1.78	1.40	2.27	2.61E-06	68	1939	2.49E-05	TRUE
p130826	E86	volume depletion	1.78	1.52	2.10	3.26E-12	153	4911	8.57E-11	TRUE
p130774	E55	vitamin d deficiency	1.95	1.57	2.41	9.97E-10	87	2405	1.50E-08	TRUE
p130718	E16	other disorders of pancreatic internal secretion	2.23	1.75	2.83	5.27E-11	69	1637	9.22E-10	TRUE
p130824	E85	amyloidosis	2.57	1.34	4.94	4.72E-03	9	182	2.61E-02	TRUE
p130706	E10	insulin-dependent diabetes mellitus	3.09	2.45	3.90	1.77E-21	73	1633	1.86E-19	TRUE

UKB: UK Biobank
AD: Alzheimer's disease
ci_min: Confidence Interval minimum
ci_max: Confidence Interval maximum
P_VAL: p-value
N_pairs: Number of individuals identified with both ICD-10 code and neurodegenerative disease outcome
n: Number of Individulas Identified with ICD10_code
P_VAL_FDR_CORRECTED: p-value after False Discovery Rate corrected
Model: ICD10 + Year_of_birth + Townsend_deprivation_index + sex

Cox proportional hazards regression analysis of Parkinson's disease and endocrine, nutritional, metabolic, and digestive system disorders ICD-10 codes adjusted for year of birth, Townsend deprivation index, and sex

UKB field corresponding to the ICD_10 code	ICD10_code	Definition of ICD10_code	Hazard Ratio	ci_min	ci_max	P_VAL	N_pairs	n	P_VAL_FDR_CORRECTED	rejected
p131650	K64	haemorrhoids and perianal venous thrombosis	0.59	0.50	0.71	3.62E-09	136	20006	7.10E-08	TRUE
p131654	K66	other disorders of peritoneum	0.63	0.45	0.87	5.81E-03	35	4612	4.42E-02	TRUE
p131636	K57	diverticular disease of intestine	0.69	0.62	0.77	6.24E-11	354	34801	2.04E-09	TRUE
p131648	K63	other diseases of intestine	0.69	0.59	0.80	1.34E-06	183	19139	2.20E-05	TRUE
p130792	E66	obesity	0.82	0.71	0.94	5.86E-03	211	22453	4.42E-02	TRUE
p130708	E11	non-insulin-dependent diabetes mellitus	1.21	1.08	1.36	1.36E-03	330	18815	1.33E-02	TRUE
p131600	K30	dyspepsia	1.34	1.13	1.60	8.51E-04	135	8811	9.26E-03	TRUE
p131640	K59	other functional intestinal disorders	1.56	1.38	1.76	4.81E-13	301	15431	4.71E-11	TRUE
p130714	E14	unspecified diabetes mellitus	1.61	1.39	1.86	1.81E-10	197	8658	4.44E-09	TRUE
p130770	E53	deficiency of other b group vitamins	1.72	1.30	2.29	1.88E-04	48	1907	2.30E-03	TRUE
p130718	E16	other disorders of pancreatic internal secretion	1.84	1.36	2.47	6.76E-05	44	1602	9.47E-04	TRUE
p130706	E10	insulin-dependent diabetes mellitus	2.65	2.02	3.48	2.44E-12	53	1608	1.20E-10	TRUE

UKB: UK Biobank
PD: Parkinson's disease
ci_min: Confidence Interval minimum
ci_max: Confidence Interval maximum
P_VAL: p-value
N_pairs: Number of individuals identified with both ICD-10 code and neurodegenerative disease outcome
n: Number of Individulas Identified with ICD10_code
P_VAL_FDR_CORRECTED: p-value after False Discovery Rate corrected
Model: ICD10 + Year_of_birth + Townsend_deprivation_index + sex

**Interaction terms between endocrine, nutritional, metabolic, digestive system disorders and Alzheimer’s disease polygenic risk score (excluding APOE)**													
**Interaction term**	**Odds Ratio**	**95% CI low**	**95% CI high**	**Beta**	**SE**	**95% CI low**	**95% CI high**	**z**	**P-value**	**N_pairs**	**n**	**P_VAL_FDR_CORRECTED**	**rejected**
zSCORE_without_apoe:(Other Bacterial Intestinal Infections)p130008	0.79	0.64	0.99	-0.23	0.11	-0.45	-0.01	-2.07	3.88E-02	81	3591	2.59E-01	FALSE
zSCORE_without_apoe:(Non-Insulin-Dependent Diabetes Mellitus)p130708	0.89	0.81	0.98	-0.11	0.05	-0.21	-0.02	-2.26	2.36E-02	476	19070	2.36E-01	FALSE
zSCORE_without_apoe:(Oesophagitis)p131582	0.82	0.70	0.95	-0.20	0.08	-0.35	-0.05	-2.55	1.08E-02	175	8668	2.16E-01	FALSE
AD: Alzheimer's disease													
CI: Confidence interval													
SE: Standard error													
N_pairs: Number of individuals identified with both ICD-10 code and neurodegenerative disease outcome													
n: Number of Individulas Identified with ICD10_code													
P_VAL_FDR_CORRECTED: p-value after False Discovery Rate corrected													

Interaction terms between endocrine, nutritional, metabolic, and digestive system disorders and Alzheimer’s disease polygenic risk score

Interaction term	Odds Ratio	95% CI low	95% CI high	Beta	SE	95% CI low	95% CI high	z	P-value	N_pairs	n	P_VAL_FDR_CORRECTED	rejected
zSCORE:(Non-Insulin-Dependent Diabetes Mellitus)p130708	0.91	0.84	0.99	-0.10	0.04	-0.18	-0.01	-2.27	2.30E-02	476	19070	6.57E-02	FALSE
zSCORE:(Obesity)p130792	0.84	0.75	0.93	-0.18	0.05	-0.28	-0.07	-3.24	1.18E-03	260	22645	7.89E-03	TRUE
zSCORE:(Disorders Of Lipoprotein Metabolism And Other Lipidaemias)p130814	0.92	0.87	0.98	-0.08	0.03	-0.14	-0.02	-2.52	1.16E-02	1090	53859	4.71E-02	TRUE
zSCORE:(Disorders Of Mineral Metabolism)p130820	0.74	0.61	0.88	-0.31	0.09	-0.49	-0.12	-3.29	9.95E-04	94	4440	7.89E-03	TRUE
zSCORE:(Volume Depletion)p130826	0.86	0.75	0.99	-0.15	0.07	-0.28	-0.01	-2.16	3.07E-02	153	4913	7.68E-02	FALSE
zSCORE:(Other Disorders Of Fluid, Electrolyte And Acid-Base Balance)p130828	0.88	0.80	0.97	-0.13	0.05	-0.23	-0.03	-2.52	1.18E-02	312	11321	4.71E-02	TRUE
zSCORE:(Oesophagitis)p131582	0.86	0.76	0.98	-0.15	0.07	-0.28	-0.02	-2.29	2.18E-02	175	8668	6.57E-02	FALSE
zSCORE:(Gastritis And Duodenitis)p131598	0.92	0.85	1.00	-0.08	0.04	-0.16	0.00	-2.00	4.58E-02	523	27949	1.02E-01	FALSE
zSCORE:(Other Functional Intestinal Disorders)p131640	0.81	0.74	0.89	-0.21	0.05	-0.30	-0.12	-4.52	6.09E-06	395	15620	1.22E-04	TRUE

AD: Alzheimer's disease
CI: Confidence interval
SE: Standard error
N_pairs: Number of individuals identified with both ICD-10 code and neurodegenerative disease outcome
n: Number of Individulas Identified with ICD10_code
P_VAL_FDR_CORRECTED: p-value after False Discovery Rate corrected

Interaction terms between endocrine, nutritional, metabolic, digestive system disorders and Parkinson's disease polygenic risk score

Interaction term	Odds Ratio	95% CI low	95% CI high	Beta	SE	95% CI low	95% CI high	z	P-value	N_pairs	n	P_VAL_FDR_CORRECTED	rejected
zSCORE:(insulin-dependent diabetes mellitus)p130706	0.76	0.58	0.99	-0.28	0.14	-0.55	-0.01	-2.02	4.36E-02	53	1613	1.05E-01	FALSE
zSCORE:(non-insulin-dependent diabetes mellitus)p130708	0.84	0.75	0.94	-0.17	0.06	-0.29	-0.06	-2.94	3.32E-03	333	18927	1.55E-02	TRUE
zSCORE:(deficiency of other b group vitamins)p130770	0.70	0.52	0.94	-0.36	0.15	-0.65	-0.06	-2.39	1.69E-02	48	1922	5.07E-02	FALSE
zSCORE:(other functional intestinal disorders)p131640	0.84	0.74	0.94	-0.18	0.06	-0.30	-0.06	-2.89	3.87E-03	303	15528	1.55E-02	TRUE
zSCORE:(other disorders of peritoneum)p131654	0.60	0.43	0.84	-0.52	0.17	-0.85	-0.18	-3.00	2.67E-03	36	4635	1.55E-02	TRUE

PD: Parkinson's disease
CI: Confidence interval
SE: Standard error
N_pairs: Number of individuals identified with both ICD-10 code and neurodegenerative disease outcome
n: Number of Individulas Identified with ICD10_code
P_VAL_FDR_CORRECTED: p-value after False Discovery Rate corrected

Biomarkers Associated with AD								
Olink_marker	Olink_marker_definition	Odds Ratio	ci_min	ci_max	P_VAL	Bonferroni_Significant	P_VAL_FDR_CORRECTED	rejected
tnfsf10	Tumor necrosis factor ligand superfamily member 10	0.56	0.41	0.77	3.37E-04	FALSE	2.68E-02	TRUE
nptxr	Neuronal pentraxin receptor	0.57	0.44	0.73	1.28E-05	TRUE	2.67E-03	TRUE
igfbp3	Insulin-like growth factor-binding protein 3	0.60	0.46	0.77	5.86E-05	FALSE	7.80E-03	TRUE
hpgds	Hematopoietic prostaglandin D synthase	0.65	0.52	0.82	2.27E-04	FALSE	2.07E-02	TRUE
bcan	Brevican core protein	0.65	0.52	0.82	2.04E-04	FALSE	1.99E-02	TRUE
cst5	Cystatin-D	0.67	0.57	0.78	2.08E-07	TRUE	7.75E-05	TRUE
adamts8	A disintegrin and metalloproteinase with thrombospondin motifs 8	0.70	0.59	0.84	7.00E-05	FALSE	7.88E-03	TRUE
psg1	Pregnancy-specific beta-1-glycoprotein 1	1.19	1.11	1.29	4.94E-06	TRUE	1.20E-03	TRUE
adgrg1	Adhesion G-protein coupled receptor G1	1.20	1.10	1.31	6.74E-05	FALSE	7.88E-03	TRUE
ren	Renin	1.22	1.11	1.34	2.33E-05	TRUE	3.41E-03	TRUE
timp4	Metalloproteinase inhibitor 4	1.38	1.15	1.67	6.74E-04	FALSE	4.49E-02	TRUE
pvr	Poliovirus receptor	1.50	1.22	1.85	1.51E-04	FALSE	1.58E-02	TRUE
calb1	Calbindin	1.50	1.24	1.82	2.29E-05	TRUE	3.41E-03	TRUE
tcn2	Transcobalamin-2	1.51	1.20	1.89	3.48E-04	FALSE	2.68E-02	TRUE
gdf15	Growth/differentiation factor 15	1.52	1.30	1.78	2.12E-07	TRUE	7.75E-05	TRUE
il1rl1	Interleukin-1 receptor-like 1	1.52	1.28	1.82	3.56E-06	TRUE	1.04E-03	TRUE
ltbp2	Latent-transforming growth factor beta-binding protein 2	1.67	1.32	2.11	1.53E-05	TRUE	2.80E-03	TRUE
il1r1	Interleukin-1 receptor type 1	1.87	1.31	2.66	5.13E-04	FALSE	3.57E-02	TRUE
igf2r	Cation-independent mannose-6-phosphate receptor	1.88	1.32	2.66	3.94E-04	FALSE	2.88E-02	TRUE
dcn	Decorin	1.89	1.34	2.67	2.75E-04	FALSE	2.36E-02	TRUE
nefl	Neurofilament light polypeptide	2.50	2.12	2.95	4.39E-28	TRUE	3.21E-25	TRUE
gfap	Glial fibrillary acidic protein	3.03	2.60	3.53	2.16E-45	TRUE	3.16E-42	TRUE

AD: Alzheimer's disease
ci_min: Confidence interval minimum
ci_max: Confidence interval maximum
P_VAL: p-value
P_VAL_FDR_CORRECTED: p-value after False Discovery Rate corrected
Model: AD ~ biomarker + zSCORE + Age_at_recruitment + Townsend_deprivation_index + sex + p22009_a1 + p22009_a2 + p22009_a3 + p22009_a4 + p22009_a5

Biomarkers Associated with PD								
**Olink_marker**	**Olink_marker_definition**	**Odds Ratio**	**ci_min**	**ci_max**	**P_VAL**	**Bonferroni_Significant**	**P_VAL_FDR_CORRECTED**	**rejected**
itgav	Integrin alpha-V	0.12	0.081	0.19	3.54E-23	TRUE	5.19E-20	TRUE
vat1	Synaptic vesicle membrane protein VAT-1 homolog	0.37	0.252	0.54	3.06E-07	TRUE	2.80E-05	TRUE
egfr	Epidermal growth factor receptor	0.37	0.240	0.57	6.45E-06	TRUE	4.10E-04	TRUE
megf9	Multiple epidermal growth factor-like domains protein 9	0.43	0.298	0.63	1.05E-05	TRUE	5.67E-04	TRUE
adgrg2	Adhesion G-protein coupled receptor G2	0.44	0.340	0.58	1.58E-09	TRUE	2.56E-07	TRUE
tnxb	Tenascin-X	0.45	0.345	0.59	1.18E-08	TRUE	1.57E-06	TRUE
itgam	Integrin alpha-M	0.45	0.358	0.58	7.87E-11	TRUE	1.64E-08	TRUE
il13ra1	Interleukin-13 receptor subunit alpha-1	0.46	0.335	0.64	3.57E-06	TRUE	2.49E-04	TRUE
itgb1	Integrin beta-1	0.47	0.332	0.67	2.63E-05	TRUE	1.07E-03	TRUE
cd99	CD99 antigen	0.47	0.324	0.69	9.33E-05	FALSE	2.90E-03	TRUE
clec10a	C-type lectin domain family 10 member A	0.48	0.390	0.59	4.72E-12	TRUE	2.30E-09	TRUE
itga11	Integrin alpha-11	0.49	0.400	0.61	2.75E-11	TRUE	1.01E-08	TRUE
setmar	Histone-lysine N-methyltransferase SETMAR	0.50	0.396	0.63	9.17E-09	TRUE	1.34E-06	TRUE
itgb2	Integrin beta-2	0.50	0.388	0.66	3.29E-07	TRUE	2.83E-05	TRUE
hpgds	Hematopoietic prostaglandin D synthase	0.51	0.416	0.62	5.45E-11	TRUE	1.55E-08	TRUE
bag3	BAG family molecular chaperone regulator 3	0.51	0.441	0.60	1.02E-17	TRUE	7.45E-15	TRUE
hyou1	Hypoxia up-regulated protein 1	0.53	0.387	0.73	7.55E-05	FALSE	2.57E-03	TRUE
f9	Coagulation factor IX	0.53	0.365	0.78	1.15E-03	FALSE	1.77E-02	TRUE
eps8l2	Epidermal growth factor receptor kinase substrate 8-like protein 2	0.54	0.425	0.70	1.26E-06	TRUE	9.19E-05	TRUE
nomo1	Nodal modulator 1	0.54	0.401	0.74	1.01E-04	FALSE	3.07E-03	TRUE
cdon	Cell adhesion molecule-related/down-regulated by oncogenes	0.55	0.419	0.72	1.15E-05	TRUE	5.98E-04	TRUE
ezr	Ezrin	0.55	0.406	0.74	9.30E-05	FALSE	2.90E-03	TRUE
cant1	Soluble calcium-activated nucleotidase 1	0.55	0.384	0.78	9.96E-04	FALSE	1.57E-02	TRUE
smad5	Mothers against decapentaplegic homolog 5	0.55	0.387	0.78	8.62E-04	FALSE	1.48E-02	TRUE
ptprn2	Receptor-type tyrosine-protein phosphatase N2	0.55	0.441	0.69	1.36E-07	TRUE	1.53E-05	TRUE
scg2	Secretogranin-2	0.55	0.441	0.69	1.68E-07	TRUE	1.76E-05	TRUE
erbb3	Receptor tyrosine-protein kinase erbB-3	0.56	0.381	0.82	2.79E-03	FALSE	3.18E-02	TRUE
klk8	Kallikrein-8	0.57	0.460	0.71	2.54E-07	TRUE	2.48E-05	TRUE
dctpp1	dCTP pyrophosphatase 1	0.57	0.457	0.71	9.16E-07	TRUE	7.10E-05	TRUE
crhbp	Corticotropin-releasing factor-binding protein	0.57	0.445	0.74	1.96E-05	TRUE	8.96E-04	TRUE
boc	Brother of CDO	0.58	0.423	0.79	6.99E-04	FALSE	1.31E-02	TRUE
ifnlr1	Interferon lambda receptor 1	0.59	0.473	0.74	5.47E-06	TRUE	3.64E-04	TRUE
tppp3	Tubulin polymerization-promoting protein family member 3	0.60	0.476	0.75	1.05E-05	TRUE	5.67E-04	TRUE
rtbdn	Retbindin	0.60	0.466	0.77	7.80E-05	FALSE	2.58E-03	TRUE
angptl3	Angiopoietin-related protein 3	0.60	0.491	0.74	9.22E-07	TRUE	7.10E-05	TRUE
tnfsf10	Tumor necrosis factor ligand superfamily member 10	0.60	0.457	0.80	4.58E-04	FALSE	1.01E-02	TRUE
ptprf	Receptor-type tyrosine-protein phosphatase F	0.61	0.472	0.78	7.39E-05	FALSE	2.57E-03	TRUE
hnmt	Histamine N-methyltransferase	0.61	0.526	0.71	6.35E-11	TRUE	1.55E-08	TRUE
st6gal1	Beta-galactoside alpha-2,6-sialyltransferase 1	0.62	0.483	0.79	1.05E-04	FALSE	3.10E-03	TRUE
erp44	Endoplasmic reticulum resident protein 44	0.62	0.464	0.82	9.54E-04	FALSE	1.55E-02	TRUE
cd99l2	CD99 antigen-like protein 2	0.62	0.461	0.84	1.71E-03	FALSE	2.32E-02	TRUE
comp	Cartilage oligomeric matrix protein	0.62	0.499	0.77	2.03E-05	TRUE	9.00E-04	TRUE
calb2	Calretinin	0.62	0.483	0.80	2.77E-04	FALSE	7.16E-03	TRUE
asgr1	Asialoglycoprotein receptor 1	0.62	0.495	0.79	7.09E-05	FALSE	2.53E-03	TRUE
furin	Furin	0.62	0.507	0.77	9.61E-06	TRUE	5.62E-04	TRUE
igfbp7	Insulin-like growth factor-binding protein 7	0.63	0.494	0.81	2.80E-04	FALSE	7.16E-03	TRUE
robo2	Roundabout homolog 2	0.63	0.463	0.87	4.29E-03	FALSE	4.24E-02	TRUE
apom	Apolipoprotein M	0.63	0.496	0.81	2.75E-04	FALSE	7.16E-03	TRUE
erbb2	Receptor tyrosine-protein kinase erbB-2	0.64	0.473	0.85	2.59E-03	FALSE	3.08E-02	TRUE
rgmb	Repulsive guidance molecule B	0.64	0.486	0.83	9.36E-04	FALSE	1.54E-02	TRUE
ptprs	Receptor-type tyrosine-protein phosphatase S	0.64	0.477	0.85	2.23E-03	FALSE	2.79E-02	TRUE
efna1	Ephrin-A1	0.64	0.493	0.83	6.92E-04	FALSE	1.31E-02	TRUE
colec12	Collectin-12	0.64	0.491	0.83	7.87E-04	FALSE	1.37E-02	TRUE
matn2	Matrilin-2	0.64	0.503	0.82	4.72E-04	FALSE	1.01E-02	TRUE
col18a1	Collagen alpha-1(XVIII) chain	0.64	0.478	0.87	3.91E-03	FALSE	3.99E-02	TRUE
tafa5	Chemokine-like protein TAFA-5	0.65	0.527	0.79	2.34E-05	TRUE	9.83E-04	TRUE
tgfbr3	Transforming growth factor beta receptor type 3	0.65	0.519	0.81	1.06E-04	FALSE	3.10E-03	TRUE
vasn	Vasorin	0.65	0.496	0.85	1.44E-03	FALSE	2.11E-02	TRUE
ggh	Gamma-glutamyl hydrolase	0.65	0.526	0.80	7.01E-05	FALSE	2.53E-03	TRUE
itgb6	Integrin beta-6	0.65	0.513	0.83	4.49E-04	FALSE	1.01E-02	TRUE
crtac1	Cartilage acidic protein 1	0.65	0.518	0.82	2.69E-04	FALSE	7.16E-03	TRUE
prcp	Lysosomal Pro-X carboxypeptidase	0.65	0.520	0.82	3.07E-04	FALSE	7.50E-03	TRUE
acvrl1	Serine/threonine-protein kinase receptor R3	0.66	0.493	0.88	4.91E-03	FALSE	4.60E-02	TRUE
dpp4	Dipeptidyl peptidase 4	0.66	0.509	0.86	2.12E-03	FALSE	2.67E-02	TRUE
lgals1	Galectin-1	0.66	0.540	0.82	1.17E-04	FALSE	3.28E-03	TRUE
ogfr	Opioid growth factor receptor	0.67	0.530	0.84	4.62E-04	FALSE	1.01E-02	TRUE
pamr1	Inactive serine protease PAMR1	0.67	0.517	0.86	1.93E-03	FALSE	2.55E-02	TRUE
fap	Prolyl endopeptidase FAP	0.67	0.518	0.87	2.72E-03	FALSE	3.18E-02	TRUE
il17d	Interleukin-17D	0.67	0.520	0.87	2.68E-03	FALSE	3.16E-02	TRUE

ntf4	Neurotrophin-4	0.68	0.533	0.86	1.54E-03	FALSE	2.15E-02	TRUE
efna4	Ephrin-A4	0.68	0.537	0.86	1.50E-03	FALSE	2.15E-02	TRUE
cd93	Complement component C1q receptor	0.68	0.536	0.87	2.28E-03	FALSE	2.82E-02	TRUE
thop1	Thimet oligopeptidase	0.69	0.539	0.87	2.09E-03	FALSE	2.67E-02	TRUE
tnfrsf13b	Tumor necrosis factor receptor superfamily member 13B	0.70	0.560	0.87	1.20E-03	FALSE	1.80E-02	TRUE
il6r	Interleukin-6 receptor subunit alpha	0.70	0.566	0.86	9.36E-04	FALSE	1.54E-02	TRUE
itgb5	Integrin beta-5	0.70	0.572	0.86	5.55E-04	FALSE	1.10E-02	TRUE
cxcl17	C-X-C motif chemokine 17	0.70	0.595	0.82	1.77E-05	TRUE	8.37E-04	TRUE
cdh2	Cadherin-2	0.70	0.574	0.86	5.43E-04	FALSE	1.09E-02	TRUE
clec14a	C-type lectin domain family 14 member A	0.70	0.558	0.89	2.78E-03	FALSE	3.18E-02	TRUE
tfpi	Tissue factor pathway inhibitor	0.71	0.554	0.90	4.65E-03	FALSE	4.39E-02	TRUE
slitrk6	SLIT and NTRK-like protein 6	0.71	0.570	0.88	1.54E-03	FALSE	2.15E-02	TRUE
mfap5	Microfibrillar-associated protein 5	0.71	0.599	0.83	3.68E-05	FALSE	1.42E-03	TRUE
enah	Protein enabled homolog	0.71	0.603	0.83	2.84E-05	TRUE	1.12E-03	TRUE
prss27	Serine protease 27	0.71	0.591	0.85	2.84E-04	FALSE	7.16E-03	TRUE
blmh	Bleomycin hydrolase	0.71	0.564	0.90	4.35E-03	FALSE	4.27E-02	TRUE
lama4	Laminin subunit alpha-4	0.71	0.575	0.89	2.56E-03	FALSE	3.08E-02	TRUE
art3	Ecto-ADP-ribosyltransferase 3	0.71	0.581	0.88	1.53E-03	FALSE	2.15E-02	TRUE
ctsd	Cathepsin D	0.72	0.596	0.88	1.18E-03	FALSE	1.79E-02	TRUE
myoc	Myocilin	0.73	0.617	0.85	1.19E-04	FALSE	3.28E-03	TRUE
il16	Pro-interleukin-16	0.73	0.609	0.87	3.88E-04	FALSE	9.15E-03	TRUE
nxph1	Neurexophilin-1	0.73	0.595	0.89	1.82E-03	FALSE	2.43E-02	TRUE
sost	Sclerostin	0.73	0.607	0.88	7.27E-04	FALSE	1.34E-02	TRUE
ret	Proto-oncogene tyrosine-protein kinase receptor Ret	0.73	0.607	0.88	7.48E-04	FALSE	1.34E-02	TRUE
ccl23	C-C motif chemokine 23	0.73	0.612	0.87	4.59E-04	FALSE	1.01E-02	TRUE
agrn	Agrin	0.73	0.594	0.90	3.68E-03	FALSE	3.82E-02	TRUE
crip2	Cysteine-rich protein 2	0.74	0.601	0.90	3.31E-03	FALSE	3.61E-02	TRUE
clec4c	C-type lectin domain family 4 member C	0.74	0.633	0.86	7.95E-05	FALSE	2.58E-03	TRUE
dsg3	Desmoglein-3	0.74	0.599	0.91	5.04E-03	FALSE	4.70E-02	TRUE
p4hb	Protein disulfide-isomerase	0.74	0.611	0.90	2.09E-03	FALSE	2.67E-02	TRUE
xg	Glycoprotein Xg	0.74	0.611	0.90	2.10E-03	FALSE	2.67E-02	TRUE
cdhr5	Cadherin-related family member 5	0.74	0.623	0.88	7.70E-04	FALSE	1.36E-02	TRUE
vwa1	von Willebrand factor A domain-containing protein 1	0.74	0.626	0.88	7.40E-04	FALSE	1.34E-02	TRUE
ism1	Isthmin-1	0.75	0.620	0.90	1.80E-03	FALSE	2.41E-02	TRUE
clec4a	C-type lectin domain family 4 member A	0.75	0.610	0.91	4.27E-03	FALSE	4.24E-02	TRUE
ly6d	Lymphocyte antigen 6D	0.75	0.622	0.92	4.48E-03	FALSE	4.31E-02	TRUE
vwc2	Brorin	0.76	0.631	0.91	2.77E-03	FALSE	3.18E-02	TRUE
fabp9	Fatty acid-binding protein 9	0.77	0.672	0.88	1.17E-04	FALSE	3.28E-03	TRUE
pcsk9	Proprotein convertase subtilisin/kexin type 9	0.77	0.645	0.92	4.04E-03	FALSE	4.08E-02	TRUE
fst	Follistatin	0.77	0.664	0.90	8.90E-04	FALSE	1.51E-02	TRUE
fcn2	Ficolin-2	0.77	0.655	0.91	2.29E-03	FALSE	2.82E-02	TRUE
cd70	CD70 antigen	0.78	0.655	0.92	3.23E-03	FALSE	3.57E-02	TRUE
ccl27	C-C motif chemokine 27	0.78	0.691	0.88	3.78E-05	FALSE	1.42E-03	TRUE
fcrl2	Fc receptor-like protein 2	0.78	0.672	0.90	9.94E-04	FALSE	1.57E-02	TRUE
s100a11	Protein S100-A11	0.79	0.663	0.93	5.12E-03	FALSE	4.71E-02	TRUE
sftpd	Pulmonary surfactant-associated protein D	0.79	0.708	0.88	1.31E-05	TRUE	6.41E-04	TRUE
fcrl1	Fc receptor-like protein 1	0.79	0.673	0.92	3.24E-03	FALSE	3.57E-02	TRUE
spink6	Serine protease inhibitor Kazal-type 6	0.79	0.692	0.90	6.09E-04	FALSE	1.18E-02	TRUE
adamts15	A disintegrin and metalloproteinase with thrombospondin motifs 15	0.79	0.682	0.92	2.83E-03	FALSE	3.21E-02	TRUE
stc1	Stanniocalcin-1	0.79	0.675	0.93	5.30E-03	FALSE	4.82E-02	TRUE
lamp3	Lysosome-associated membrane glycoprotein 3	0.80	0.701	0.91	1.08E-03	FALSE	1.68E-02	TRUE
dpy30	Protein dpy-30 homolog	0.80	0.707	0.91	4.76E-04	FALSE	1.01E-02	TRUE
ldlr	Low-density lipoprotein receptor	0.80	0.698	0.92	1.62E-03	FALSE	2.23E-02	TRUE
sumf2	Inactive C-alpha-formylglycine-generating enzyme 2	0.80	0.703	0.92	1.18E-03	FALSE	1.79E-02	TRUE
fabp4	Fatty acid-binding protein, adipocyte	0.80	0.713	0.91	3.37E-04	FALSE	8.08E-03	TRUE
krt5	Keratin, type II cytoskeletal 5	0.80	0.690	0.94	5.11E-03	FALSE	4.71E-02	TRUE
inpp1	Inositol polyphosphate 1-phosphatase	0.80	0.699	0.93	2.59E-03	FALSE	3.08E-02	TRUE
serpinb8	Serpin B8	0.81	0.705	0.92	1.46E-03	FALSE	2.12E-02	TRUE
tnfsf11	Tumor necrosis factor ligand superfamily member 11	0.81	0.716	0.91	5.19E-04	FALSE	1.05E-02	TRUE
ctsb	Cathepsin B	0.81	0.704	0.93	2.35E-03	FALSE	2.87E-02	TRUE
aif1	Allograft inflammatory factor 1	0.81	0.703	0.93	3.44E-03	FALSE	3.70E-02	TRUE
ccl16	C-C motif chemokine 16	0.81	0.713	0.92	1.71E-03	FALSE	2.32E-02	TRUE
slc39a5	Zinc transporter ZIP5	0.81	0.706	0.94	3.81E-03	FALSE	3.93E-02	TRUE
il11	Interleukin-11	0.82	0.716	0.94	3.93E-03	FALSE	3.99E-02	TRUE
selp	P-selectin	0.82	0.724	0.94	3.50E-03	FALSE	3.71E-02	TRUE
ces3	Carboxylesterase 3	0.83	0.738	0.92	9.03E-04	FALSE	1.52E-02	TRUE
fbp1	Fructose-1,6-bisphosphatase 1	0.84	0.756	0.92	4.64E-04	FALSE	1.01E-02	TRUE
clc	Galectin-10	0.84	0.746	0.94	2.08E-03	FALSE	2.67E-02	TRUE
gpr37	Prosaposin receptor GPR37	0.85	0.762	0.95	3.60E-03	FALSE	3.76E-02	TRUE
fabp5	Fatty acid-binding protein 5	0.85	0.763	0.95	3.41E-03	FALSE	3.70E-02	TRUE
crh	Corticoliberin	0.87	0.795	0.95	1.28E-03	FALSE	1.89E-02	TRUE
tcl1a	T-cell leukemia/lymphoma protein 1A	0.88	0.820	0.95	7.52E-04	FALSE	1.34E-02	TRUE

pspn	Persephin	0.90	0.830	0.97	4.46E-03	FALSE	4.31E-02	TRUE
ppy	Pancreatic prohormone	0.90	0.840	0.97	4.20E-03	FALSE	4.21E-02	TRUE
mep1b	Meprin A subunit beta	0.92	0.864	0.97	4.53E-03	FALSE	4.33E-02	TRUE
epcam	Epithelial cell adhesion molecule	1.12	1.039	1.20	2.93E-03	FALSE	3.29E-02	TRUE
prdx1	Peroxiredoxin-1	1.12	1.033	1.21	5.51E-03	FALSE	4.98E-02	TRUE
lxn	Latexin	1.16	1.050	1.28	3.54E-03	FALSE	3.73E-02	TRUE
padi2	Protein-arginine deiminase type-2	1.21	1.085	1.34	5.07E-04	FALSE	1.04E-02	TRUE
il1rl1	Interleukin-1 receptor-like 1	1.27	1.080	1.48	3.48E-03	FALSE	3.71E-02	TRUE
mmp13	Collagenase 3	1.39	1.106	1.74	4.65E-03	FALSE	4.39E-02	TRUE
dsg2	Desmoglein-2	1.49	1.145	1.94	2.98E-03	FALSE	3.32E-02	TRUE
ebi3_il27	Interleukin-27	1.50	1.189	1.89	6.14E-04	FALSE	1.18E-02	TRUE
ncam1	Neural cell adhesion molecule 1	1.51	1.209	1.89	2.96E-04	FALSE	7.34E-03	TRUE
cd276	CD276 antigen	1.58	1.278	1.95	2.35E-05	TRUE	9.83E-04	TRUE
mertk	Tyrosine-protein kinase Mer	1.60	1.210	2.12	9.84E-04	FALSE	1.57E-02	TRUE
nefl	Neurofilament light polypeptide	1.62	1.395	1.88	2.19E-10	TRUE	4.01E-08	TRUE
PD: Parkinson's disease								
ci_min: Confidence interval minimum								
ci_max: Confidence interval maximum								
P_VAL: p-value								
P_VAL_FDR_CORRECTED: p-value after False Discovery Rate corrected								
Model: PD ~ biomarker + zSCORE + Age_at_recruitment + Townsend_deprivation_index + sex + p22009_a1 + p22009_a2 + p22009_a3 + p22009_a4 + p22009_a5								

Performance summary of different feature sets for AD classification

Feature Set	Number of Features	Train AUC	Test AUC	Train Balanced Accuracy	Test Balanced Accuracy	Sensitivity	Specificity	Train AUC_CI	Test AUC_CI	Train Balanced Accuracy_CI	Test Balanced Accuracy_CI	Sensitivity_CI	Specificity_CI
Genetics	7	0.81	0.74	0.73	0.69	0.634	0.74	0.81 ± 0.01	0.74 ± 0.04	0.73 ± 0.02	0.69 ± 0.03	0.63 ± 0.11	0.74 ± 0.09
Clinical	17	0.66	0.61	0.64	0.60	0.506	0.69	0.66 ± 0.02	0.61 ± 0.01	0.64 ± 0.01	0.60 ± 0.02	0.51 ± 0.05	0.69 ± 0.05
Olink	10	0.94	0.87	0.87	0.79	0.776	0.80	0.94 ± 0.01	0.87 ± 0.02	0.87 ± 0.02	0.79 ± 0.03	0.78 ± 0.07	0.80 ± 0.05
Demographics	3	0.85	0.82	0.78	0.75	0.780	0.72	0.85 ± 0.02	0.82 ± 0.05	0.78 ± 0.02	0.75 ± 0.03	0.78 ± 0.06	0.72 ± 0.11
Combined without Clinical	20	0.94	0.89	0.87	0.82	0.820	0.82	0.94 ± 0.00	0.89 ± 0.02	0.87 ± 0.01	0.82 ± 0.04	0.82 ± 0.06	0.82 ± 0.04
Combined	37	0.95	0.90	0.88	0.83	0.820	0.83	0.95 ± 0.02	0.90 ± 0.02	0.88 ± 0.02	0.83 ± 0.03	0.82 ± 0.06	0.83 ± 0.04

AD: Alzheimer's Disease

AUC: area under the receiver operating characteristic (ROC) curve

Performance summary of different feature sets for PD classification

Feature Set	Number of Features	Train AUC	Test AUC	Train Balanced Accuracy	Test Balanced Accuracy	Sensitivity	Specificity	Train AUC_CI	Test AUC_CI	Train Balanced Accuracy_CI	Test Balanced Accuracy_CI	Sensitivity_CI	Specificity_CI
Genetics	10	0.71	0.58	0.66	0.56	0.571	0.56	0.71 ± 0.14	0.58 ± 0.04	0.66 ± 0.11	0.56 ± 0.02	0.57 ± 0.12	0.56 ± 0.10
Clinical	7	0.55	0.52	0.54	0.53	0.141	0.91	0.55 ± 0.02	0.52 ± 0.03	0.54 ± 0.01	0.53 ± 0.03	0.14 ± 0.04	0.91 ± 0.09
Olink	34	0.91	0.73	0.83	0.67	0.690	0.65	0.91 ± 0.05	0.73 ± 0.03	0.83 ± 0.08	0.67 ± 0.03	0.69 ± 0.05	0.65 ± 0.06
Demographics	3	0.80	0.77	0.73	0.72	0.822	0.61	0.80 ± 0.01	0.77 ± 0.03	0.73 ± 0.01	0.72 ± 0.04	0.82 ± 0.10	0.61 ± 0.10
Combined without Clinical	47	0.90	0.78	0.81	0.71	0.793	0.62	0.90 ± 0.07	0.78 ± 0.03	0.81 ± 0.10	0.71 ± 0.02	0.79 ± 0.03	0.62 ± 0.06
Combined	54	0.88	0.78	0.78	0.70	0.804	0.60	0.88 ± 0.03	0.78 ± 0.04	0.78 ± 0.04	0.70 ± 0.02	0.80 ± 0.04	0.60 ± 0.04

PD: Parkinson's Disease

AUC: area under the receiver operating characteristic (ROC) curve

ICD 10 codes used to create control cohort

ICD-10 Code	Description
G10	Huntington's Disease
G11	Hereditary Ataxia
G12	Spinal Muscular Atrophy and Related Syndromes
G13	Systemic Atrophies Primarily Affecting Central Nervous System in Diseases Classified Elsewhere
G14	Postpolio Syndrome
G20	Parkinson's Disease
G21	Secondary Parkinsonism
G22	Parkinsonism in Diseases Classified Elsewhere
G23	Other Degenerative Diseases of Basal Ganglia
G24	Dystonia
G25	Other Extrapyramidal and Movement Disorders
G30	Alzheimer's Disease
G31	Other Degenerative Diseases of Nervous System, Not Elsewhere Classified
G32	Other Degenerative Disorders of Nervous System in Diseases Classified Elsewhere
G35	Multiple Sclerosis
G36	Other Acute Disseminated Demyelination
G37	Other Demyelinating Diseases of Central Nervous System
G45	Transient Cerebral Ischaemic Attacks and Related Syndromes
G46	Vascular Syndromes of Brain in Cerebrovascular Diseases
G50	Disorders of Trigeminal Nerve
G52	Disorders of Other Cranial Nerves
G53	Cranial Nerve Disorders in Diseases Classified Elsewhere
G54	Nerve Root and Plexus Disorders
G55	Nerve Root and Plexus Compressions in Diseases Classified Elsewhere
G56	Mononeuropathies of Upper Limb
G57	Mononeuropathies of Lower Limb
G58	Other Mononeuropathies
G59	Mononeuropathy in Diseases Classified Elsewhere
G60	Hereditary and Idiopathic Neuropathy
G61	Inflammatory Polyneuropathy
G62	Other Polyneuropathies
G63	Polyneuropathy in Diseases Classified Elsewhere
G64	Other Disorders of Peripheral Nervous System
G70	Myasthenia Gravis and Other Myoneural Disorders
G71	Primary Disorders of Muscles
G72	Other Myopathies
G73	Disorders of Myoneural Junction and Muscle in Diseases Classified Elsewhere
G80	Infantile Cerebral Palsy
G81	Hemiplegia
G82	Paraplegia and Tetraplegia
G83	Other Paralytic Syndromes
G90	Disorders of Autonomic Nervous System

ICD 10 codes used to create control cohort

ICD-10 Code	Description
G91	Hydrocephalus
G92	Toxic Encephalopathy
G93	Other Disorders of Brain
G94	Other Disorders of Brain in Diseases Classified Elsewhere
G96	Other Disorders of Central Nervous System
G97	Postprocedural Disorders of Nervous System, Not Elsewhere Classified
G98	Other Disorders of Nervous System, Not Elsewhere Classified
G99	Other Disorders of Nervous System in Diseases Classified Elsewhere

Biobank cohort gender and Alzheimer’s disease/Parkinson’s disease demographics

	UKB			SAIL			Finngen		
**Neurodegenerative disease**	**All**	**Female**	**Male**	**All**	**Female**	**Male**	**All**	**Female**	**Male**
AD	3,308	1,721	1,587	38,973	24,743	14,230	15,617	6,875	8,742
PD	2,780	1,024	1,756	24,270	11,387	12,883	4,681	1,858	2,823
controls	261,814	138,666	123,148	2,106,924	1,129,391	977,533	412,181	230,310	181,871

UKB: UK Biobank
SAIL: Secure Anonymised Information Linkage Databank
AD: Alzheimer's disease
PD: Parkinson's disease

AD for UKB is G30 and F00 ICD10 codes
Finngen summary stat https://r10.risteys.finngen.fi/endpoints/G6_AD_WIDE
https://r10.risteys.finngen.fi/endpoints/G6_PARKINSON
https://www.finngen.fi/en/access_results

UKB diagnosis and disorders under study			
UKB field corresponding to the ICD_10 code	ICD10_code	Definition of ICD10_code	
p130000	A00	Cholera	
p130002	A01	Typhoid And Paratyphoid Fevers	
p130004	A02	Other Salmonella Infections	
p130006	A03	Shigellosis	
p130008	A04	Other Bacterial Intestinal Infections	
p130010	A05	Other Bacterial Foodborne Intoxications	
p130012	A06	Amoebiasis	
p130014	A07	Other Protozoal Intestinal Diseases	
p130016	A08	Viral And Other Specified Intestinal Infections	
p130018	A09	Diarrhoea And Gastro-Enteritis Of Presumed Infectious Origin	
p131552	K00	Disorders Of Tooth Development And Eruption	
p131554	K01	Embedded And Impacted Teeth	
p131556	K02	Dental Caries	
p131558	K03	Other Diseases Of Hard Tissues Of Teeth	
p131560	K04	Diseases Of Pulp And Periapical Tissues	
p131562	K05	Gingivitis And Periodontal Diseases	
p131564	K06	Other Disorders Of Gingiva And Edentulous Alveolar Ridge	
p131566	K07	Dentofacial Anomalies [Including Malocclusion]	
p131568	K08	Other Disorders Of Teeth And Supporting Structures	
p131570	K09	Cysts Of Oral Region, Not Elsewhere Classified	
p131572	K10	Other Diseases Of Jaws	
p131574	K11	Diseases Of Salivary Glands	
p131576	K12	Stomatitis And Related Lesions	
p131578	K13	Other Diseases Of Lip And Oral Mucosa	
p131580	K14	Diseases Of Tongue	
p131582	K20	Oesophagitis	
p131584	K21	Gastro-Oesophageal Reflux Disease	
p131586	K22	Other Diseases Of Oesophagus	
p131588	K23	Disorders Of Oesophagus In Diseases Classified Elsewhere	
p131590	K25	Gastric Ulcer	
p131592	K26	Duodenal Ulcer	
p131594	K27	Peptic Ulcer, Site Unspecified	
p131596	K28	Gastrojejunal Ulcer	
p131598	K29	Gastritis And Duodenitis	
p131600	K30	Dyspepsia	
p131602	K31	Other Diseases Of Stomach And Duodenum	
p131604	K35	Acute Appendicitis	
p131606	K36	Other Appendicitis	
p131608	K37	Unspecified Appendicitis	
p131610	K38	Other Diseases Of Appendix	
p131612	K40	Inguinal Hernia	
p131614	K41	Femoral Hernia	
p131616	K42	Umbilical Hernia	
p131618	K43	Ventral Hernia	
p131620	K44	Diaphragmatic Hernia	
p131622	K45	Other Abdominal Hernia	
p131624	K46	Unspecified Abdominal Hernia	
p131626	K50	Crohn'S Disease [Regional Enteritis]	
p131628	K51	Ulcerative Colitis	
p131630	K52	Other Non-Infective Gastro-Enteritis And Colitis	
p131632	K55	Vascular Disorders Of Intestine	
p131634	K56	Paralytic Ileus And Intestinal Obstruction Without Hernia	
p131636	K57	Diverticular Disease Of Intestine	
p131638	K58	Irritable Bowel Syndrome	
p131640	K59	Other Functional Intestinal Disorders	
p131642	K60	Fissure And Fistula Of Anal And Rectal Regions	
p131644	K61	Abscess Of Anal And Rectal Regions	
p131646	K62	Other Diseases Of Anus And Rectum	
p131648	K63	Other Diseases Of Intestine	
p131650	K64	Haemorrhoids And Perianal Venous Thrombosis	
p131652	K65	Peritonitis	

UKB diagnosis and disorders under study			
**UKB field corresponding to the ICD_10 code**	**ICD10_code**	**Definition of ICD10_code**	
p131654	K66	Other Disorders Of Peritoneum	
p131656	K67	Disorders Of Peritoneum In Infectious Diseases Classified Elsewhere	
p131658	K70	Alcoholic Liver Disease	
p131660	K71	Toxic Liver Disease	
p131662	K72	Hepatic Failure, Not Elsewhere Classified	
p131664	K73	Chronic Hepatitis, Not Elsewhere Classified	
p131666	K74	Fibrosis And Cirrhosis Of Liver	
p131668	K75	Other Inflammatory Liver Diseases	
p131670	K76	Other Diseases Of Liver	
p131672	K77	Liver Disorders In Diseases Classified Elsewhere	
p131674	K80	Cholelithiasis	
p131676	K81	Cholecystitis	
p131678	K82	Other Diseases Of Gallbladder	
p131680	K83	Other Diseases Of Biliary Tract	
p131682	K85	Acute Pancreatitis	
p131684	K86	Other Diseases Of Pancreas	
p131686	K87	Disorders Of Gallbladder, Biliary Tract And Pancreas In Diseases Classified Elsewhere	
p131688	K90	Intestinal Malabsorption	
p131690	K91	Postprocedural Disorders Of Digestive System, Not Elsewhere Classified	
p131692	K92	Other Diseases Of Digestive System	
p131694	K93	Disorders Of Other Digestive Organs In Diseases Classified Elsewhere	
p130690	E00	Congenital Iodine-Deficiency Syndrome	
p130692	E01	Iodine-Deficiency-Related Thyroid Disorders And Allied Conditions	
p130694	E02	Subclinical Iodine-Deficiency Hypothyroidism	
p130696	E03	Other Hypothyroidism	
p130698	E04	Other Non-Toxic Goitre	
p130700	E05	Thyrotoxicosis [Hyperthyroidism]	
p130702	E06	Thyroiditis	
p130704	E07	Other Disorders Of Thyroid	
p130706	E10	Insulin-Dependent Diabetes Mellitus	
p130708	E11	Non-Insulin-Dependent Diabetes Mellitus	
p130710	E12	Malnutrition-Related Diabetes Mellitus	
p130712	E13	Other Specified Diabetes Mellitus	
p130714	E14	Unspecified Diabetes Mellitus	
p130716	E15	Nondiabetic Hypoglycaemic Coma	
p130718	E16	Other Disorders Of Pancreatic Internal Secretion	
p130720	E20	Hypoparathyroidism	
p130722	E21	Hyperparathyroidism And Other Disorders Of Parathyroid Gland	
p130724	E22	Hyperfunction Of Pituitary Gland	
p130726	E23	Hypofunction And Other Disorders Of Pituitary Gland	
p130728	E24	Cushing'S Syndrome	
p130730	E25	Adrenogenital Disorders	
p130732	E26	Hyperaldosteronism	
p130734	E27	Other Disorders Of Adrenal Gland	
p130736	E28	Ovarian Dysfunction	
p130738	E29	Testicular Dysfunction	
p130740	E30	Disorders Of Puberty, Not Elsewhere Classified	
p130742	E31	Polyglandular Dysfunction	
p130744	E32	Diseases Of Thymus	
p130746	E34	Other Endocrine Disorders	
p130748	E35	Disorders Of Endocrine Glands In Diseases Classified Elsewhere	
p130750	E40	Kwashiorkor	
p130752	E41	Nutritional Marasmus	
p130754	E42	Marasmic Kwashiorkor	
p130756	E43	Unspecified Severe Protein-Energy Malnutrition	
p130758	E44	Protein-Energy Malnutrition Of Moderate And Mild Degree	
p130760	E45	Retarded Development Following Protein-Energy Malnutrition	
p130762	E46	Unspecified Protein-Energy Malnutrition	
p130764	E50	Vitamin A Deficiency	
p130766	E51	Thiamine Deficiency	
p130768	E52	Niacin Deficiency [Pellagra]	

UKB diagnosis and disorders under study		
**UKB field corresponding to the ICD_10 code**	**ICD10_code**	**Definition of ICD10_code**
p130770	E53	Deficiency Of Other B Group Vitamins
p130772	E54	Ascorbic Acid Deficiency
p130774	E55	Vitamin D Deficiency
p130776	E56	Other Vitamin Deficiencies
p130778	E58	Dietary Calcium Deficiency
p130780	E59	Dietary Selenium Deficiency
p130782	E60	Dietary Zinc Deficiency
p130784	E61	Deficiency Of Other Nutrient Elements
p130786	E63	Other Nutritional Deficiencies
p130788	E64	Sequelae Of Malnutrition And Other Nutritional Deficiencies
p130790	E65	Localised Adiposity
p130792	E66	Obesity
p130794	E67	Other Hyperalimentation
p130796	E68	Sequelae Of Hyperalimentation
p130798	E70	Disorders Of Aromatic Amino-Acid Metabolism
p130800	E71	Disorders Of Branched-Chain Amino-Acid Metabolism And Fatty-Acid Metabolism
p130802	E72	Other Disorders Of Amino-Acid Metabolism
p130804	E73	Lactose Intolerance
p130806	E74	Other Disorders Of Carbohydrate Metabolism
p130808	E75	Disorders Of Sphingolipid Metabolism And Other Lipid Storage Disorders
p130810	E76	Disorders Of Glycosaminoglycan Metabolism
p130812	E77	Disorders Of Glycoprotein Metabolism
p130814	E78	Disorders Of Lipoprotein Metabolism And Other Lipidaemias
p130816	E79	Disorders Of Purine And Pyrimidine Metabolism
p130818	E80	Disorders Of Porphyrin And Bilirubin Metabolism
p130820	E83	Disorders Of Mineral Metabolism
p130822	E84	Cystic Fibrosis
p130824	E85	Amyloidosis
p130826	E86	Volume Depletion
p130828	E87	Other Disorders Of Fluid, Electrolyte And Acid-Base Balance
p130830	E88	Other Metabolic Disorders
p130832	E89	Postprocedural Endocrine And Metabolic Disorders, Not Elsewhere Classified
p130834	E90	Nutritional And Metabolic Disorders In Diseases Classified Elsewhere
UKB: UK Biobank		

Proteomic biomarkers under study
**Olink proteomic targets**
aarsd1
abhd14b
abl1
acaa1
acan
ace2
acox1
acp5
acp6
acta2
actn4
acvrl1
acy1
ada
ada2
adam15
adam22
adam23
adam8
adamts13
adamts15
adamts16
adamts8
adcyap1r1
adgrb3
adgre2
adgre5
adgrg1
adgrg2
adh4
adm
afp
ager
agr2
agr3
agrn
agrp
agxt
ahcy
ahsp
aif1
aifm1

Proteomic biomarkers under study
**Olink proteomic targets**
ak1
akr1b1
akr1c4
akt1s1
akt3
alcam
aldh1a1
aldh3a1
alpp
ambn
ambp
amfr
amigo2
amn
amy2a
amy2b
ang
angpt1
angpt2
angptl1
angptl2
angptl3
angptl4
angptl7
ankrd54
anpep
anxa10
anxa11
anxa3
anxa4
anxa5
aoc1
aoc3
apbb1ip
apex1
aplp1
apoh
apom
app
aprt
areg
arg1

Proteomic biomarkers under study
**Olink proteomic targets**
arhgap1
arhgap25
arhgef12
arid4b
arnt
arsa
arsb
art3
artn
asah2
asgr1
atf2
atg4a
atox1
atp5if1
atp5po
atp6ap2
atp6v1d
atp6v1f
atxn10
axin1
axl
azu1
b4galt1
b4gat1
bach1
bag3
bag6
baiap2
bambi
bank1
bax
bcam
bcan
bcl2l11
bcr
bgn
bid
bin2
birc2
blmh
blvrb

Proteomic biomarkers under study
**Olink proteomic targets**
bmp4
bmp6
boc
bpifb1
brk1
bsg
bst1
bst2
btc
btn2a1
btn3a2
c19orf12
c1qa
c1qtnf1
c2
c2cd2l
c4bpb
ca1
ca11
ca12
ca13
ca14
ca2
ca3
ca4
ca5a
ca6
ca9
calb1
calb2
calca
calcoco1
camkk1
cant1
capg
carhsp1
casp1
casp10
casp2
casp3
casp8
cblif

Proteomic biomarkers under study
**Olink proteomic targets**
cbln4
cc2d1a
ccdc80
ccl11
ccl13
ccl14
ccl15
ccl16
ccl17
ccl18
ccl19
ccl2
ccl20
ccl21
ccl22
ccl23
ccl24
ccl25
ccl26
ccl27
ccl28
ccl3
ccl4
ccl5
ccl7
ccl8
ccn1
ccn2
ccn3
ccn4
ccn5
ccs
cct5
cd109
cd14
cd160
cd163
cd164
cd177
cd1c
cd200
cd200r1

Proteomic biomarkers under study
**Olink proteomic targets**
cd207
cd209
cd22
cd244
cd27
cd274
cd276
cd28
cd2ap
cd300c
cd300e
cd300lf
cd300lg
cd302
cd33
cd34
cd38
cd4
cd40
cd40lg
cd46
cd48
cd5
cd55
cd58
cd59
cd6
cd63
cd69
cd70
cd74
cd79b
cd83
cd84
cd8a
cd93
cd99
cd99l2
cdc27
cdc37
cdcp1
cdh1

Proteomic biomarkers under study
**Olink proteomic targets**
cdh15
cdh17
cdh2
cdh3
cdh5
cdh6
cdhr1
cdhr2
cdhr5
cdkn1a
cdkn2d
cdnf
cdon
cdsn
ceacam1
ceacam21
ceacam3
ceacam5
ceacam8
cebpb
cela3a
cep164
cep20
cep43
cep85
cert
ces1
ces2
ces3
cetn2
cfc1
cga
cgref1
chac2
chek2
chgb
chi3l1
chit1
chl1
chmp1a
chrdl1
chrdl2

Proteomic biomarkers under study
**Olink proteomic targets**
ciapin1
ckap4
ckmt1a_ckmt1b
clc
clec10a
clec11a
clec14a
clec1a
clec1b
clec4a
clec4c
clec4d
clec4g
clec5a
clec6a
clec7a
clip2
clmp
clpp
clps
clspn
clstn1
clstn2
clta
clul1
cndp1
cnpy2
cnpy4
cnst
cntn1
cntn2
cntn3
cntn4
cntn5
cntnap2
col18a1
col1a1
col4a1
col6a3
col9a1
colec12
comp

Proteomic biomarkers under study
**Olink proteomic targets**
comt
cope
coro1a
cox5b
cpa1
cpa2
cpb1
cpe
cpm
cpped1
cpvl
cpxm1
cr2
cracr2a
cradd
creg1
creld2
crh
crhbp
crhr1
crim1
crip2
crisp2
crkl
crlf1
crnn
crtac1
crtam
crx
csf1
csf2ra
csf3
cst3
cst5
cst6
cst7
cstb
ctf1
ctrb1
ctrc
ctsb
ctsc

Proteomic biomarkers under study
**Olink proteomic targets**
ctsd
ctsf
ctsh
ctsl
ctso
ctss
ctsv
ctsz
cx3cl1
cxadr
cxcl1
cxcl10
cxcl11
cxcl12
cxcl13
cxcl14
cxcl16
cxcl17
cxcl3
cxcl5
cxcl6
cxcl8
cxcl9
dab2
dag1
dapp1
dars1
dbi
dbnl
dcbld2
dcn
dctn1
dctn2
dctn6
dctpp1
dcxr
ddah1
ddc
ddr1
ddx58
decr1
defa1_defa1b

Proteomic biomarkers under study
**Olink proteomic targets**
defb4a_defb4b
dffa
dgkz
diablo
dkk1
dkk3
dkk4
dkkl1
dlk1
dll1
dnaja2
dnajb1
dnajb8
dner
dnmbp
dnph1
dok2
dpep1
dpep2
dpp10
dpp4
dpp6
dpp7
dpt
dpy30
draxin
drg2
dsc2
dsg2
dsg3
dsg4
dtx3
duox2
dusp3
ebag9
ebi3_il27
ece1
eda2r
edar
edil3
efemp1
efna1

Proteomic biomarkers under study
**Olink proteomic targets**
efna4
egf
egfl7
egfr
egln1
eif4b
eif4ebp1
eif4g1
eif5a
eloa
enah
eng
eno1
eno2
enpp2
enpp5
enpp7
entpd2
entpd5
entpd6
epcam
epha1
epha10
epha2
ephb4
ephb6
ephx2
epo
eps8l2
erbb2
erbb3
erbb4
erbin
ereg
erp44
esam
esm1
ezr
f11r
f2r
f3
f7

Proteomic biomarkers under study
**Olink proteomic targets**
f9
fabp1
fabp2
fabp4
fabp5
fabp6
fabp9
fadd
fam3b
fam3c
fap
fas
faslg
fbp1
fcar
fcer2
fcgr2a
fcgr2b
fcgr3b
fcn2
fcrl1
fcrl2
fcrl3
fcrl5
fcrl6
fcrlb
fen1
fes
fetub
fgf19
fgf2
fgf21
fgf23
fgf5
fgfbp1
fgfr2
fgr
fhit
fis1
fkbp1b
fkbp4
fkbp5

Proteomic biomarkers under study
**Olink proteomic targets**
fkbp7
fli1
flrt2
flt1
flt3
flt3lg
flt4
fmnl1
fmr1
folr1
folr2
folr3
fosb
foxo1
foxo3
frzb
fst
fstl3
fuca1
furin
fus
fut3_fut5
fut8
fxn
fxyd5
fyb1
gal
galnt10
galnt2
galnt3
galnt7
gas6
gbp2
gbp4
gcg
gcnt1
gdf15
gdf2
gdnf
gfap
gfer
gfod2

Proteomic biomarkers under study
**Olink proteomic targets**
gfra1
gfra2
gfra3
gga1
ggh
ggt1
ggt5
gh1
gh2
ghrhr
ghrl
gkn1
glb1
glo1
glod4
glrx
glt8d2
gmpr
gne
gnly
golm2
gopc
gp1ba
gp2
gp6
gpa33
gpc1
gpc5
gpkow
gpnmb
gpr37
grap2
grk5
grn
grpel1
gsap
gsta1
gsta3
gstp1
guca2a
gusb
gys1

Proteomic biomarkers under study
**Olink proteomic targets**
gzma
gzmb
gzmh
hagh
hao1
hars1
havcr1
havcr2
hbegf
hbq1
hcls1
hdgf
hebp1
hexim1
hgf
hgs
hk2
hla_dra
hla_e
hmbs
hmox1
hmox2
hnmt
hnrnpk
hpcal1
hpgds
hs3st3b1
hs6st1
hsd11b1
hsp90b1
hspa1a
hspb1
hspb6
hspg2
htra2
hyal1
hyou1
ica1
icam1
icam2
icam3
icam4

Proteomic biomarkers under study
**Olink proteomic targets**
icam5
icoslg
idi2
ids
idua
ifng
ifngr1
ifngr2
ifnl1
ifnlr1
igf1r
igf2r
igfbp1
igfbp2
igfbp3
igfbp4
igfbp6
igfbp7
igfbpl1
igsf3
igsf8
ikbkg
ikzf2
il10
il10ra
il10rb
il11
il12a_il12b
il12b
il12rb1
il13
il13ra1
il15
il15ra
il16
il17a
il17c
il17d
il17f
il17ra
il17rb
il18

Proteomic biomarkers under study
**Olink proteomic targets**
il18bp
il18r1
il18rap
il19
il1a
il1b
il1r1
il1r2
il1rap
il1rl1
il1rl2
il1rn
il2
il20
il20ra
il22ra1
il24
il2ra
il2rb
il32
il33
il34
il3ra
il4
il4r
il5
il5ra
il6
il6r
il6st
il7
il7r
ilkap
impa1
ing1
inhbc
inpp1
inppl1
ipcef1
iqgap2
irag2
irak1

Proteomic biomarkers under study
**Olink proteomic targets**
irak4
islr2
ism1
itga11
itga5
itga6
itgam
itgav
itgb1
itgb1bp1
itgb1bp2
itgb2
itgb5
itgb6
itgb7
itih3
itm2a
ivd
jam2
jchain
jun
kazald1
kcnip4
kdr
kel
kifbp
kir2dl3
kir3dl1
kirrel2
kit
kitlg
klb
klk1
klk10
klk11
klk12
klk13
klk14
klk4
klk6
klk8
klrb1

Proteomic biomarkers under study
**Olink proteomic targets**
klrd1
krt14
krt18
krt19
krt5
kyat1
kynu
l1cam
lactb2
lag3
lair1
lair2
lama4
lamp2
lamp3
lap3
lat
lat2
layn
lbp
lbr
lcn2
ldlr
lefty2
lep
lepr
lgals1
lgals3
lgals4
lgals7_lgals7b
lgals8
lgals9
lgmn
lhb
lhpp
lif
lifr
lilra2
lilra5
lilrb1
lilrb2
lilrb4

Proteomic biomarkers under study
**Olink proteomic targets**
lilrb5
lpcat2
lpl
lpo
lrig1
lrp1
lrp11
lrpap1
lrrc25
lrrn1
lsm1
lsp1
lta
lta4h
ltbp2
ltbp3
ltbr
lto1
lxn
ly6d
ly75
ly9
ly96
lyar
lyn
lypd1
lypd3
lypd8
mad1l1
maea
maged1
manf
mansc1
map2k6
map3k5
map4k5
mapk9
mapt
marco
masp1
matn2
matn3

Proteomic biomarkers under study
**Olink proteomic targets**
mavs
max
mb
mcam
mcfd2
mdga1
mdk
med18
megf10
megf9
mep1b
mepe
mertk
mesd
met
metap1
metap1d
metap2
mfap3
mfap5
mfge8
mgll
mgmt
mia
micb_mica
mif
milr1
mitd1
mln
mme
mmp1
mmp10
mmp12
mmp13
mmp3
mmp7
mmp8
mmp9
mnda
mog
mphosph8
mpi

Proteomic biomarkers under study
**Olink proteomic targets**
mpig6b
mpo
mrpl46
msln
msmb
msr1
msra
mstn
mtpn
muc13
muc16
mvk
myo9b
myoc
mzb1
mzt1
naaa
nadk
nampt
nbl1
nbn
ncam1
ncam2
ncan
ncf2
nck2
ncln
ncr1
ncs1
ndrg1
ndufs6
nectin2
nectin4
nefl
nell1
nell2
nfasc
nfatc1
nfatc3
nfkbie
ngf
nid1

Proteomic biomarkers under study
**Olink proteomic targets**
nid2
ninj1
nme3
nmnat1
nomo1
nos1
nos3
notch1
notch3
npdc1
npm1
nppb
nppc
nptn
nptx1
nptxr
npy
nrcam
nrp1
nrp2
nrtn
nsfl1c
nt5c3a
nt5e
ntf3
ntf4
ntprobnp
ntrk2
ntrk3
nub1
nucb2
nudc
nudt2
nudt5
nxph1
obp2b
odam
ogfr
ogn
olr1
omd
omg

**Proteomic biomarkers under study**
**Olink proteomic targets**
optc
oscar
osm
osmr
oxt
p4hb
padi2
padi4
paep
pag1
pak4
pam
pamr1
pappa
park7
parp1
pbld
pcdh1
pcdh17
pcolce
pcsk9
pdcd1
pdcd1lg2
pdcd5
pdcd6
pdgfa
pdgfb
pdgfc
pdgfra
pdgfrb
pdlim7
pdp1
pear1
pebp1
pecam1
pfdn2
pfkfb2
pgf
pglyrp1
phospho1
pi3
pigr

Proteomic biomarkers under study
**Olink proteomic targets**
pik3ap1
pik3ip1
pilra
pilrb
pklr
pla2g10
pla2g15
pla2g1b
pla2g2a
pla2g4a
pla2g7
plat
plau
plaur
plin1
plin3
plpbp
pltp
plxdc1
plxna4
plxnb2
plxnb3
pm20d1
pmvk
pnliprp2
pnpt1
podxl
podxl2
polr2f
pon2
pon3
ppcdc
ppib
ppm1a
ppme1
ppp1r12a
ppp1r2
ppp1r9b
ppp3r1
ppy
pqbp1
prcp

Proteomic biomarkers under study
**Olink proteomic targets**
prdx1
prdx3
prdx5
prdx6
preb
prelp
prkab1
prkar1a
prkcq
prkra
prl
proc
prok1
prss2
prss27
prss8
prtfdc1
prtg
prtn3
psg1
psip1
psma1
psmd9
psme1
psme2
psmg3
pspn
psrc1
pten
ptgds
pth1r
ptk7
ptn
ptpn1
ptpn6
ptprf
ptprm
ptprn2
ptprs
pts
ptx3
pvalb

Proteomic biomarkers under study
**Olink proteomic targets**
pvr
pxn
qdpr
qpct
rab37
rab6a
rab6b
rabepk
rabgap1l
rad23b
rangap1
rarres1
rarres2
rasa1
rassf2
rbks
rbp2
rbp5
rcor1
reg1a
reg1b
reg3a
reg4
relt
ren
ret
retn
rgma
rgmb
rgs8
rhoc
rilp
rnase3
rnaset2
rnf41
robo1
robo2
ror1
rp2
rrm2
rrm2b
rspo1

Proteomic biomarkers under study
**Olink proteomic targets**
rspo3
rtbdn
rtn4r
ruvbl1
rwdd1
s100a11
s100a12
s100a16
s100a4
s100p
samd9l
scamp3
scara5
scarb1
scarb2
scarf1
scarf2
scg2
scg3
scgb1a1
scgb3a2
scgn
scly
scp2
scrn1
sdc1
sdc4
sele
selp
selplg
sema3f
sema4c
sema4d
sema7a
septin9
serpina11
serpina12
serpina9
serpinb1
serpinb5
serpinb6
serpinb8

Proteomic biomarkers under study
**Olink proteomic targets**
serpinb9
serpine1
sestd1
setmar
sez6l
sez6l2
sf3b4
sfrp1
sftpa1
sftpa2
sftpd
sh2b3
sh2d1a
shmt1
siae
siglec1
siglec10
siglec15
siglec5
siglec6
siglec7
siglec9
sirpa
sirpb1
sirt2
sirt5
sit1
skap1
skap2
slamf1
slamf6
slamf7
slamf8
slc16a1
slc27a4
slc39a14
slc39a5
slit2
slitrk2
slitrk6
smad1
smad5

Proteomic biomarkers under study
**Olink proteomic targets**
smarca2
smoc1
smoc2
smpd1
smpdl3a
snap23
snap29
sncg
snx9
sod1
sod2
sorcs2
sord
sort1
sost
sparc
sparcl1
spink1
spink4
spink5
spink6
spint1
spint2
spock1
spon1
spon2
spp1
spry2
src
srp14
srpk2
ssb
ssc4d
ssc5d
st3gal1
st6gal1
stambp
stat5b
stc1
stc2
stip1
stk11

Proteomic biomarkers under study
**Olink proteomic targets**
stk24
stk4
stx16
stx4
stx6
stx8
stxbp3
sugt1
sult1a1
sult2a1
sumf2
susd1
susd2
tacc3
tacstd2
tafa5
tank
tarbp2
tbc1d17
tbc1d23
tbc1d5
tbcb
tbcc
tbl1x
tcl1a
tcl1b
tcn2
tdgf1
tdrkh
tek
tff1
tff2
tff3
tfpi
tfpi2
tfrc
tgfa
tgfb1
tgfbi
tgfbr2
tgfbr3
tgm2

Proteomic biomarkers under study
**Olink proteomic targets**
thbd
thbs2
thbs4
thop1
thpo
thy1
tia1
tie1
tigar
timd4
timp1
timp3
timp4
tinagl1
tjap1
tlr3
tmprss15
tmprss5
tmsb10
tnc
tnf
tnfaip8
tnfrsf10a
tnfrsf10b
tnfrsf10c
tnfrsf11a
tnfrsf11b
tnfrsf12a
tnfrsf13b
tnfrsf13c
tnfrsf14
tnfrsf19
tnfrsf1a
tnfrsf1b
tnfrsf21
tnfrsf4
tnfrsf6b
tnfrsf8
tnfrsf9
tnfsf10
tnfsf11
tnfsf12

Proteomic biomarkers under study
**Olink proteomic targets**
tnfsf13
tnfsf13b
tnfsf14
tnni3
tnr
tnxb
tp53
tp53inp1
tpmt
tpp1
tppp3
tpsab1
tpt1
traf2
trem2
treml2
triap1
trim21
trim5
tshb
tslp
tspan1
tst
txlna
txndc15
txndc5
txnrd1
tymp
tyro3
ubac1
ulbp2
umod
uso1
usp8
uxs1
vamp5
vash1
vasn
vat1
vcam1
vcan
vegfa

Proteomic biomarkers under study
**Olink proteomic targets**
vegfc
vegfd
vim
vmo1
vnn2
vps37a
vps53
vsig4
vsir
vstm1
vstm2l
vta1
vtcn1
vwa1
vwc2
vwf
wars
was
wasf1
wasf3
wfdc12
wfdc2
wfikkn1
wfikkn2
wif1
wnt9a
wwp2
xcl1
xg
xpnpep2
xrcc4
yes1
ythdf3
zbtb16
zbtb17

Cox proportional hazards regression analysis of Alzheimer's disease and endocrine, nutritional, metabolic, and digestive system disorders ICD-10 codes adjusted for APOE4/4 status, principal components 1-5, year of birth, Townsend deprivation index, and sex

UKB field corresponding to the ICD_10 code	ICD10_code	Definition of ICD10_code	Hazard Ratio	ci_min	ci_max	P_VAL	N_pairs	n	P_VAL_FDR_CORRECTED	rejected
p131654	K66	Other Disorders Of Peritoneum	0.63	0.46	0.85	2.84E-03	41	4636	1.75E-02	TRUE
p131650	K64	Haemorrhoids And Perianal Venous Thrombosis	0.70	0.60	0.81	2.14E-06	186	20162	2.25E-05	TRUE
p131598	K29	Gastritis And Duodenitis	1.23	1.12	1.35	1.42E-05	523	27914	1.24E-04	TRUE
p131582	K20	Oesophagitis	1.28	1.10	1.49	1.55E-03	175	8660	1.08E-02	TRUE
p130820	E83	Disorders Of Mineral Metabolism	1.33	1.09	1.64	5.90E-03	94	4435	3.44E-02	TRUE
p131630	K52	Other Non-Infective Gastro-Enteritis And Colitis	1.36	1.20	1.55	3.04E-06	250	12984	2.90E-05	TRUE
p131560	K04	Diseases Of Pulp And Periapical Tissues	1.44	1.11	1.87	6.67E-03	57	3365	3.68E-02	TRUE
p130828	E87	Other Disorders Of Fluid, Electrolyte And Acid-Base Balance	1.47	1.31	1.66	9.30E-11	312	11313	1.48E-09	TRUE
p130708	E11	Non-Insulin-Dependent Diabetes Mellitus	1.55	1.40	1.71	3.46E-18	476	19048	1.82E-16	TRUE
p131640	K59	Other Functional Intestinal Disorders	1.55	1.39	1.72	4.25E-16	394	15596	1.49E-14	TRUE
p130008	A04	Other Bacterial Intestinal Infections	1.59	1.27	1.98	4.07E-05	81	3585	3.29E-04	TRUE
p130714	E14	Unspecified Diabetes Mellitus	1.60	1.39	1.83	1.94E-11	224	8733	4.08E-10	TRUE
p130826	E86	Volume Depletion	1.71	1.45	2.02	9.89E-11	153	4911	1.48E-09	TRUE
p130770	E53	Deficiency Of Other B Group Vitamins	1.80	1.41	2.29	1.73E-06	68	1939	2.02E-05	TRUE
p130774	E55	Vitamin D Deficiency	1.84	1.49	2.28	1.99E-08	87	2405	2.62E-07	TRUE
p131658	K70	Alcoholic Liver Disease	2.14	1.31	3.50	2.49E-03	16	955	1.64E-02	TRUE
p130718	E16	Other Disorders Of Pancreatic Internal Secretion	2.34	1.84	2.97	3.49E-12	69	1637	9.16E-11	TRUE
p130706	E10	Insulin-Dependent Diabetes Mellitus	3.03	2.40	3.82	9.49E-21	73	1633	9.96E-19	TRUE

UKB: UK Biobank
AD: Alzheimer's disease
ci_min: Confidence Interval minimum
ci_max: Confidence Interval maximum
P_VAL: p-value
N_pairs: Number of individuals identified with both ICD-10 code and neurodegenerative disease outcome
n: Number of Individulas Identified with ICD10_code
P_VAL_FDR_CORRECTED: p-value after False Discovery Rate corrected
Model: ICD10 + Apo_E4E4 + p22009_a1 + p22009_a2 + p22009_a3 + p22009_a4 + p22009_a5 + Year_of_birth + Townsend_deprivation_index + sex

Cox proportional hazards regression analysis of Alzheimer's disease and endocrine, nutritional, metabolic, and digestive system disorders ICD-10 codes adjusted for polygenic risk Z-score excluding APOE, APOE4/4 status, principal components 1-5, year of birth, Townsend deprivation index, and sex

UKB field corresponding to the ICD_10 code	ICD10_code	Definition of ICD10_code	Hazard Ratio	ci_min	ci_max	P_VAL	N_pairs	n	P_VAL_FDR_CORRECTED	rejected
p131654	K66	Other Disorders Of Peritoneum	0.63	0.46	0.86	3.53E-03	41	4636	2.06E-02	TRUE
p131650	K64	Haemorrhoids And Perianal Venous Thrombosis	0.70	0.61	0.82	3.12E-06	186	20162	3.28E-05	TRUE
p131598	K29	Gastritis And Duodenitis	1.23	1.12	1.35	1.48E-05	523	27914	1.29E-04	TRUE
p131582	K20	Oesophagitis	1.27	1.09	1.48	2.17E-03	175	8660	1.52E-02	TRUE
p131630	K52	Other Non-Infective Gastro-Enteritis And Colitis	1.35	1.19	1.54	4.30E-06	250	12984	4.11E-05	TRUE
p130820	E83	Disorders Of Mineral Metabolism	1.36	1.11	1.67	3.34E-03	94	4435	2.06E-02	TRUE
p131560	K04	Diseases Of Pulp And Periapical Tissues	1.45	1.12	1.88	5.51E-03	57	3365	3.04E-02	TRUE
p130828	E87	Other Disorders Of Fluid, Electrolyte And Acid-Base Balance	1.47	1.30	1.65	1.58E-10	312	11313	2.37E-09	TRUE
p131640	K59	Other Functional Intestinal Disorders	1.55	1.39	1.72	6.59E-16	394	15596	2.31E-14	TRUE
p130708	E11	Non-Insulin-Dependent Diabetes Mellitus	1.55	1.40	1.71	3.00E-18	476	19048	1.58E-16	TRUE
p130714	E14	Unspecified Diabetes Mellitus	1.60	1.39	1.83	1.84E-11	224	8733	3.87E-10	TRUE
p130008	A04	Other Bacterial Intestinal Infections	1.62	1.30	2.02	1.69E-05	81	3585	1.36E-04	TRUE
p130826	E86	Volume Depletion	1.73	1.47	2.03	5.36E-11	153	4911	9.38E-10	TRUE
p130770	E53	Deficiency Of Other B Group Vitamins	1.80	1.42	2.29	1.70E-06	68	1939	1.98E-05	TRUE
p130774	E55	Vitamin D Deficiency	1.84	1.48	2.27	2.50E-08	87	2405	3.29E-07	TRUE
p131658	K70	Alcoholic Liver Disease	2.08	1.27	3.41	3.48E-03	16	955	2.06E-02	TRUE
p130718	E16	Other Disorders Of Pancreatic Internal Secretion	2.34	1.84	2.97	3.14E-12	69	1637	8.24E-11	TRUE
p130706	E10	Insulin-Dependent Diabetes Mellitus	3.09	2.45	3.90	1.88E-21	73	1633	1.97E-19	TRUE

PRS: Polygenic risk score
UKB: UK Biobank
AD: Alzheimer's disease
ci_min: Confidence Interval minimum
ci_max: Confidence Interval maximum
P_VAL: p-value
N_pairs: Number of individuals identified with both ICD-10 code and neurodegenerative disease outcome
n: Number of Individulas Identified with ICD10_code
P_VAL_FDR_CORRECTED: p-value after False Discovery Rate corrected
Model: ICD10 + zSCORE_without_apoe + Apo_E4E4 + p22009_a1 + p22009_a2 + p22009_a3 + p22009_a4 + p22009_a5 + Year_of_birth + Townsend_deprivation_index + sex

Cox proportional hazards regression analysis of Alzheimer's disease and endocrine, nutritional, metabolic, and digestive system disorders ICD-10 codes adjusted for polygenic risk Z-scores excluding APOE, principal components 1-5, year of birth, Townsend deprivation index, and sex

UKB field corresponding to the ICD_10 code	ICD10_code	Neurodegenera	Definition of ICD10_code	Hazard Ratio	ci_min	ci_max	P_VAL	N_pairs	n	P_VAL_FDR_CC	rejected
p131654	K66	AD	Other Disorders Of Peritoneum	0.59	0.44	0.81	8.85E-04	41	4636	6.19E-03	TRUE
p131650	K64	AD	Haemorrhoids And Perianal Venous Thrombosis	0.69	0.59	0.80	6.54E-07	186	20162	7.63E-06	TRUE
p131636	K57	AD	Diverticular Disease Of Intestine	0.89	0.81	0.97	1.01E-02	573	35207	4.80E-02	TRUE
p130814	E78	AD	Disorders Of Lipoprotein Metabolism And Other Lipidaemias	1.18	1.09	1.27	1.63E-05	1090	53797	1.43E-04	TRUE
p131598	K29	AD	Gastritis And Duodenitis	1.20	1.09	1.32	1.61E-04	523	27914	1.20E-03	TRUE
p131582	K20	AD	Oesophagitis	1.26	1.08	1.47	2.85E-03	175	8660	1.66E-02	TRUE
p130820	E83	AD	Disorders Of Mineral Metabolism	1.32	1.07	1.62	8.20E-03	94	4435	4.10E-02	TRUE
p131630	K52	AD	Other Non-Infective Gastro-Enteritis And Colitis	1.36	1.20	1.55	2.50E-06	250	12984	2.52E-05	TRUE
p131560	K04	AD	Diseases Of Pulp And Periapical Tissues	1.44	1.11	1.87	6.61E-03	57	3365	3.65E-02	TRUE
p130708	E11	AD	Non-Insulin-Dependent Diabetes Mellitus	1.48	1.34	1.63	8.67E-15	476	19048	3.04E-13	TRUE
p130828	E87	AD	Other Disorders Of Fluid, Electrolyte And Acid-Base Balance	1.51	1.34	1.70	6.83E-12	312	11313	1.43E-10	TRUE
p130714	E14	AD	Unspecified Diabetes Mellitus	1.53	1.33	1.75	1.11E-09	224	8733	1.58E-08	TRUE
p130008	A04	AD	Other Bacterial Intestinal Infections	1.53	1.23	1.91	1.54E-04	81	3585	1.20E-03	TRUE
p131640	K59	AD	Other Functional Intestinal Disorders	1.55	1.39	1.72	6.02E-16	394	15596	3.16E-14	TRUE
p130770	E53	AD	Deficiency Of Other B Group Vitamins	1.78	1.40	2.27	2.64E-06	68	1939	2.52E-05	TRUE
p130826	E86	AD	Volume Depletion	1.78	1.52	2.10	3.27E-12	153	4911	8.59E-11	TRUE
p130774	E55	AD	Vitamin D Deficiency	1.94	1.57	2.40	1.20E-09	87	2405	1.58E-08	TRUE
p130718	E16	AD	Other Disorders Of Pancreatic Internal Secretion	2.23	1.75	2.83	5.29E-11	69	1637	9.26E-10	TRUE
p130824	E85	AD	Amyloidosis	2.42	1.26	4.66	8.08E-03	9	182	4.10E-02	TRUE
p130706	E10	AD	Insulin-Dependent Diabetes Mellitus	3.13	2.48	3.95	6.06E-22	73	1633	6.36E-20	TRUE

PRS: Polygenic risk score
UKB: UK Biobank
AD: Alzheimer's disease
ci_min: Confidence Interval minimum
ci_max: Confidence Interval maximum
P_VAL: p-value
N_pairs: Number of individuals identified with both ICD-10 code and neurodegenerative disease outcome
n: Number of Individulas Identified with ICD10_code
P_VAL_FDR_CORRECTED: p-value after False Discovery Rate corrected
Model: ICD10 + zSCORE_without_apoe + p22009_a1 + p22009_a2 + p22009_a3 + p22009_a4 + p22009_a5 + Year_of_birth + Townsend_deprivation_index + sex

Cox proportional hazards regression analysis of Alzheimer's disease and endocrine, nutritional, metabolic, and digestive system disorders ICD-10 codes adjusted for polygenic risk Z-score, APOE4/4 status, principal components 1-5, year of birth, Townsend deprivation index, and sex

UKB field corresponding to the ICD_10 code	ICD10_code	Definition of ICD10_code	Hazard Ratio	ci_min	ci_max	P_VAL	N_pairs	n	P_VAL_FDR_CORRECTED	rejected
p131654	K66	Other Disorders Of Peritoneum	0.63	0.46	0.86	3.39E-03	41	4636	2.02E-02	TRUE
p131650	K64	Haemorrhoids And Perianal Venous Thrombosis	0.70	0.61	0.82	3.13E-06	186	20162	3.28E-05	TRUE
p131598	K29	Gastritis And Duodenitis	1.23	1.12	1.35	1.47E-05	523	27914	1.29E-04	TRUE
p131582	K20	Oesophagitis	1.27	1.09	1.48	2.16E-03	175	8660	1.51E-02	TRUE
p131630	K52	Other Non-Infective Gastro-Enteritis And Colitis	1.35	1.19	1.54	4.28E-06	250	12984	4.08E-05	TRUE
p130820	E83	Disorders Of Mineral Metabolism	1.36	1.11	1.67	3.34E-03	94	4435	2.02E-02	TRUE
p131560	K04	Diseases Of Pulp And Periapical Tissues	1.45	1.12	1.88	5.50E-03	57	3365	3.04E-02	TRUE
p130828	E87	Other Disorders Of Fluid, Electrolyte And Acid-Base Balance	1.47	1.30	1.65	1.56E-10	312	11313	2.34E-09	TRUE
p131640	K59	Other Functional Intestinal Disorders	1.54	1.39	1.72	7.10E-16	394	15596	2.48E-14	TRUE
p130708	E11	Non-Insulin-Dependent Diabetes Mellitus	1.55	1.40	1.71	3.18E-18	476	19048	1.67E-16	TRUE
p130714	E14	Unspecified Diabetes Mellitus	1.60	1.39	1.83	1.82E-11	224	8733	3.83E-10	TRUE
p130008	A04	Other Bacterial Intestinal Infections	1.62	1.30	2.02	1.68E-05	81	3585	1.36E-04	TRUE
p130826	E86	Volume Depletion	1.73	1.47	2.03	5.33E-11	153	4911	9.32E-10	TRUE
p130770	E53	Deficiency Of Other B Group Vitamins	1.80	1.42	2.29	1.69E-06	68	1939	1.97E-05	TRUE
p130774	E55	Vitamin D Deficiency	1.84	1.48	2.27	2.49E-08	87	2405	3.27E-07	TRUE
p131658	K70	Alcoholic Liver Disease	2.08	1.27	3.41	3.47E-03	16	955	2.02E-02	TRUE
p130718	E16	Other Disorders Of Pancreatic Internal Secretion	2.34	1.84	2.97	3.13E-12	69	1637	8.21E-11	TRUE
p130706	E10	Insulin-Dependent Diabetes Mellitus	3.09	2.45	3.90	1.87E-21	73	1633	1.96E-19	TRUE

PRS: Polygenic risk score
UKB: UK Biobank
AD: Alzheimer's disease
ci_min: Confidence Interval minimum
ci_max: Confidence Interval maximum
P_VAL: p-value
N_pairs: Number of individuals identified with both ICD-10 code and neurodegenerative disease outcome
n: Number of Individulas Identified with ICD10_code
P_VAL_FDR_CORRECTED: p-value after False Discovery Rate corrected
Model: ICD10 + zSCORE + Apo_E4E4 + p22009_a1 + p22009_a2 + p22009_a3 + p22009_a4 + p22009_a5 + Year_of_birth + Townsend_deprivation_index + sex

Cox proportional hazards regression analysis of Alzheimer's disease and endocrine, nutritional, metabolic, and digestive system disorders ICD-10 codes adjusted for polygenic risk Z-scores, principal components 1-5, year of birth, Townsend deprivation index, and sex

UKB field corresponding to the ICD_10 code	ICD10_code	Definition of ICD10_code	Hazard Ratio	ci_min	ci_max	P_VAL	N_pairs	n	P_VAL_FDR_CORRECTED	rejected
p131654	K66	Other Disorders Of Peritoneum	0.63	0.46	0.86	3.26E-03	41	4636	2.01E-02	TRUE
p131650	K64	Haemorrhoids And Perianal Venous Thrombosis	0.70	0.61	0.82	3.18E-06	186	20162	3.34E-05	TRUE
p131598	K29	Gastritis And Duodenitis	1.23	1.12	1.35	1.55E-05	523	27914	1.30E-04	TRUE
p131582	K20	Oesophagitis	1.27	1.09	1.48	2.23E-03	175	8660	1.56E-02	TRUE
p131630	K52	Other Non-Infective Gastro-Enteritis And Colitis	1.35	1.19	1.54	4.28E-06	250	12984	4.08E-05	TRUE
p130820	E83	Disorders Of Mineral Metabolism	1.36	1.11	1.67	3.22E-03	94	4435	2.01E-02	TRUE
p131560	K04	Diseases Of Pulp And Periapical Tissues	1.45	1.12	1.89	5.37E-03	57	3365	2.97E-02	TRUE
p130828	E87	Other Disorders Of Fluid, Electrolyte And Acid-Base Balance	1.47	1.30	1.65	1.54E-10	312	11313	2.31E-09	TRUE
p131640	K59	Other Functional Intestinal Disorders	1.54	1.39	1.72	8.16E-16	394	15596	2.86E-14	TRUE
p130708	E11	Non-Insulin-Dependent Diabetes Mellitus	1.55	1.40	1.71	3.75E-18	476	19048	1.97E-16	TRUE
p130714	E14	Unspecified Diabetes Mellitus	1.60	1.39	1.83	2.02E-11	224	8733	4.23E-10	TRUE
p130008	A04	Other Bacterial Intestinal Infections	1.62	1.30	2.03	1.62E-05	81	3585	1.30E-04	TRUE
p130826	E86	Volume Depletion	1.73	1.47	2.04	4.48E-11	153	4911	7.85E-10	TRUE
p130770	E53	Deficiency Of Other B Group Vitamins	1.80	1.41	2.29	1.71E-06	68	1939	1.99E-05	TRUE
p130774	E55	Vitamin D Deficiency	1.84	1.48	2.27	2.41E-08	87	2405	3.16E-07	TRUE
p131658	K70	Alcoholic Liver Disease	2.07	1.27	3.39	3.75E-03	16	955	2.19E-02	TRUE
p130718	E16	Other Disorders Of Pancreatic Internal Secretion	2.34	1.84	2.97	3.21E-12	69	1637	8.43E-11	TRUE
p130706	E10	Insulin-Dependent Diabetes Mellitus	3.10	2.46	3.91	1.55E-21	73	1633	1.63E-19	TRUE

PRS: Polygenic risk score
UKB: UK Biobank
AD: Alzheimer's disease
ci_min: Confidence Interval minimum
ci_max: Confidence Interval maximum
P_VAL: p-value
N_pairs: Number of individuals identified with both ICD-10 code and neurodegenerative disease outcome
n: Number of Individulas Identified with ICD10_code
P_VAL_FDR_CORRECTED: p-value after False Discovery Rate corrected
Model: ICD10 + zSCORE + p22009_a1 + p22009_a2 + p22009_a3 + p22009_a4 + p22009_a5 + Year_of_birth + Townsend_deprivation_index + sex

Cox proportional hazards regression analysis of Alzheimer's disease and endocrine, nutritional, metabolic, and digestive system disorders ICD-10 codes adjusted for principal components 1-5, year of birth, Townsend deprivation index, and sex

UKB field corresponding to the ICD_10 code	ICD10_code	Definition of ICD10_code	Hazard Ratio	ci_min	ci_max	P_VAL	N_pairs	n	P_VAL_FDR_CORRECTED	rejected
p131654	K66	Other Disorders Of Peritoneum	0.59	0.44	0.81	8.75E-04	41	4636	5.74E-03	TRUE
p131650	K64	Haemorrhoids And Perianal Venous Thrombosis	0.69	0.59	0.80	6.73E-07	186	20162	7.85E-06	TRUE
p130792	E66	Obesity	0.84	0.74	0.96	9.27E-03	260	22619	4.43E-02	TRUE
p131636	K57	Diverticular Disease Of Intestine	0.89	0.81	0.97	1.08E-02	573	35207	4.94E-02	TRUE
p130814	E78	Disorders Of Lipoprotein Metabolism And Other Lipidaemias	1.18	1.09	1.27	1.68E-05	1090	53797	1.47E-04	TRUE
p131598	K29	Gastritis And Duodenitis	1.20	1.09	1.32	1.59E-04	523	27914	1.28E-03	TRUE
p131582	K20	Oesophagitis	1.28	1.10	1.49	1.68E-03	175	8660	1.03E-02	TRUE
p130820	E83	Disorders Of Mineral Metabolism	1.32	1.08	1.62	8.00E-03	94	4435	4.00E-02	TRUE
p131630	K52	Other Non-Infective Gastro-Enteritis And Colitis	1.37	1.20	1.56	1.91E-06	250	12984	2.00E-05	TRUE
p131560	K04	Diseases Of Pulp And Periapical Tissues	1.44	1.11	1.87	6.17E-03	57	3365	3.24E-02	TRUE
p130708	E11	Non-Insulin-Dependent Diabetes Mellitus	1.47	1.33	1.62	1.47E-14	476	19048	5.16E-13	TRUE
p130828	E87	Other Disorders Of Fluid, Electrolyte And Acid-Base Balance	1.51	1.34	1.70	6.72E-12	312	11313	1.41E-10	TRUE
p130008	A04	Other Bacterial Intestinal Infections	1.51	1.21	1.89	2.31E-04	81	3585	1.73E-03	TRUE
p130714	E14	Unspecified Diabetes Mellitus	1.53	1.33	1.75	1.19E-09	224	8733	1.56E-08	TRUE
p131640	K59	Other Functional Intestinal Disorders	1.55	1.40	1.73	3.41E-16	394	15596	1.79E-14	TRUE
p130770	E53	Deficiency Of Other B Group Vitamins	1.77	1.39	2.26	3.15E-06	68	1939	3.00E-05	TRUE
p130826	E86	Volume Depletion	1.78	1.52	2.10	3.47E-12	153	4911	9.12E-11	TRUE
p130774	E55	Vitamin D Deficiency	1.95	1.57	2.41	9.22E-10	87	2405	1.38E-08	TRUE
p130718	E16	Other Disorders Of Pancreatic Internal Secretion	2.23	1.76	2.84	4.79E-11	69	1637	8.39E-10	TRUE
p130824	E85	Amyloidosis	2.58	1.34	4.97	4.46E-03	9	182	2.46E-02	TRUE
p130706	E10	Insulin-Dependent Diabetes Mellitus	3.09	2.45	3.90	1.80E-21	73	1633	1.89E-19	TRUE

UKB: UK Biobank
AD: Alzheimer's disease
ci_min: Confidence Interval minimum
ci_max: Confidence Interval maximum
P_VAL: p-value
N_pairs: Number of individuals identified with both ICD-10 code and neurodegenerative disease outcome
n: Number of Individulas Identified with ICD10_code
P_VAL_FDR_CORRECTED: p-value after False Discovery Rate corrected
Model: ICD10 + p22009_a1 + p22009_a2 + p22009_a3 + p22009_a4 + p22009_a5 + Year_of_birth + Townsend_deprivation_index + sex

Cox proportional hazards regression analysis of Parkinson's disease and endocrine, nutritional, metabolic, and digestive system disorders ICD-10 codes adjusted for principal components 1-5, year of birth, Townsend deprivation index, and sex

UKB field corresponding to the ICD_10 code	ICD10_code	Definition of ICD10_code	Hazard Ratio	ci_min	ci_max	P_VAL	N_pairs	n	P_VAL_FDR_CORRECTED	rejected
p131650	K64	haemorrhoids and perianal venous thrombosis	0.59	0.50	0.71	3.25E-09	136	20006	6.36E-08	TRUE
p131654	K66	other disorders of peritoneum	0.62	0.45	0.87	5.59E-03	35	4612	4.21E-02	TRUE
p131636	K57	diverticular disease of intestine	0.69	0.62	0.77	7.26E-11	354	34801	2.37E-09	TRUE
p131648	K63	other diseases of intestine	0.69	0.59	0.80	1.38E-06	183	19139	2.25E-05	TRUE
p130792	E66	obesity	0.82	0.71	0.94	4.91E-03	211	22453	4.21E-02	TRUE
p130708	E11	non-insulin-dependent diabetes mellitus	1.21	1.08	1.36	1.40E-03	330	18815	1.37E-02	TRUE
p131600	K30	dyspepsia	1.34	1.13	1.59	9.03E-04	135	8811	9.83E-03	TRUE
p131640	K59	other functional intestinal disorders	1.56	1.38	1.76	5.23E-13	301	15431	5.12E-11	TRUE
p130714	E14	unspecified diabetes mellitus	1.61	1.39	1.86	2.06E-10	197	8658	5.04E-09	TRUE
p130770	E53	deficiency of other b group vitamins	1.73	1.30	2.31	1.62E-04	48	1907	1.98E-03	TRUE
p130718	E16	other disorders of pancreatic internal secretion	1.82	1.35	2.46	8.06E-05	44	1602	1.13E-03	TRUE
p130706	E10	insulin-dependent diabetes mellitus	2.64	2.01	3.46	2.96E-12	53	1608	1.45E-10	TRUE

UKB: UK Biobank
PD: Parkinson's disease
ci_min: Confidence Interval minimum
ci_max: Confidence Interval maximum
P_VAL: p-value
N_pairs: Number of individuals identified with both ICD-10 code and neurodegenerative disease outcome
n: Number of Individulas Identified with ICD10_code
P_VAL_FDR_CORRECTED: p-value after False Discovery Rate corrected
Model: ICD10 + p22009_a1 + p22009_a2 + p22009_a3 + p22009_a4 + p22009_a5 +Year_of_birth + Townsend_deprivation_index + sex

Cox proportional hazards regression analysis of Parkinson's disease and endocrine, nutritional, metabolic, and digestive system disorders ICD-10 codes adjusted for polygenic risk Z-scores, principal components 1-5, Townsend deprivation index, and sex										
**UKB field corresponding to the ICD_10 code**	**ICD10_code**	**Definition of ICD10_code**	**Hazard Ratio**	**ci_min**	**ci_max**	**P_VAL**	**N_pairs**	**n**	**P_VAL_FDR_CORRECTED**	**rejected**
p131650	K64	haemorrhoids and perianal venous thrombosis	0.59	0.50	0.71	3.30E-09	136	20006	6.47E-08	TRUE
p131654	K66	other disorders of peritoneum	0.62	0.45	0.87	5.63E-03	35	4612	4.59E-02	TRUE
p131648	K63	other diseases of intestine	0.69	0.59	0.80	1.12E-06	183	19139	1.83E-05	TRUE
p131636	K57	diverticular disease of intestine	0.69	0.62	0.77	8.13E-11	354	34801	2.66E-09	TRUE
p130792	E66	obesity	0.82	0.71	0.95	6.35E-03	211	22453	4.78E-02	TRUE
p130708	E11	non-insulin-dependent diabetes mellitus	1.21	1.08	1.36	1.04E-03	330	18815	1.12E-02	TRUE
p131600	K30	dyspepsia	1.33	1.12	1.59	1.14E-03	135	8811	1.12E-02	TRUE
p131640	K59	other functional intestinal disorders	1.55	1.38	1.75	8.76E-13	301	15431	7.99E-11	TRUE
p130714	E14	unspecified diabetes mellitus	1.61	1.39	1.86	1.67E-10	197	8658	4.09E-09	TRUE
p130770	E53	deficiency of other b group vitamins	1.73	1.30	2.30	1.70E-04	48	1907	2.09E-03	TRUE
p130718	E16	other disorders of pancreatic internal secretion	1.81	1.34	2.44	9.86E-05	44	1602	1.38E-03	TRUE
p130706	E10	insulin-dependent diabetes mellitus	2.67	2.03	3.50	1.63E-12	53	1608	7.99E-11	TRUE

PRS: Polygenic risk score
UKB: UK Biobank
PD: Parkinson's disease
ci_min: Confidence Interval minimum
ci_max: Confidence Interval maximum
P_VAL: p-value
N_pairs: Number of individuals identified with both ICD-10 code and neurodegenerative disease outcome
n: Number of Individulas Identified with ICD10_code
P_VAL_FDR_CORRECTED: p-value after False Discovery Rate corrected
Model: ICD10 + zSCORE + p22009_a1 + p22009_a2 + p22009_a3 + p22009_a4 + p22009_a5 + Year_of_birth + Townsend_deprivation_index + sex

Cox proportional hazards regression analysis of Parkinson's disease and endocrine, nutritional, metabolic, and digestive system disorders ICD-10 codes adjusted for GBA1 (G_1_155162560 + T_1_155235843) status, principal components 1-5, year of birth, Townsend deprivation index, and sex

UKB field corresponding to the ICD_10 code	ICD10_code	Definition of ICD10_code	Hazard Ratio	ci_min	ci_max	P_VAL	N_pairs	n	P_VAL_FDR_CORRECTED	rejected
p131650	K64	haemorrhoids and perianal venous thrombosis	0.59	0.50	0.71	3.24E-09	136	20006	6.36E-08	TRUE
p131654	K66	other disorders of peritoneum	0.62	0.45	0.87	5.62E-03	35	4612	4.33E-02	TRUE
p131636	K57	diverticular disease of intestine	0.69	0.62	0.77	7.52E-11	354	34801	2.46E-09	TRUE
p131648	K63	other diseases of intestine	0.69	0.59	0.80	1.38E-06	183	19139	2.25E-05	TRUE
p130792	E66	obesity	0.82	0.71	0.94	5.02E-03	211	22453	4.33E-02	TRUE
p130708	E11	non-insulin-dependent diabetes mellitus	1.21	1.07	1.36	1.58E-03	330	18815	1.55E-02	TRUE
p131600	K30	dyspepsia	1.34	1.12	1.59	1.04E-03	135	8811	1.13E-02	TRUE
p131640	K59	other functional intestinal disorders	1.56	1.38	1.76	4.76E-13	301	15431	4.67E-11	TRUE
p130714	E14	unspecified diabetes mellitus	1.60	1.38	1.85	2.99E-10	197	8658	7.32E-09	TRUE
p130770	E53	deficiency of other b group vitamins	1.73	1.30	2.30	1.70E-04	48	1907	2.08E-03	TRUE
p130718	E16	other disorders of pancreatic internal secretion	1.81	1.34	2.44	9.93E-05	44	1602	1.39E-03	TRUE
p130706	E10	insulin-dependent diabetes mellitus	2.63	2.00	3.45	3.44E-12	53	1608	1.68E-10	TRUE

UKB: UK Biobank
PD: Parkinson's disease
ci_min: Confidence Interval minimum
ci_max: Confidence Interval maximum
P_VAL: p-value
N_pairs: Number of individuals identified with both ICD-10 code and neurodegenerative disease outcome
n: Number of Individulas Identified with ICD10_code
P_VAL_FDR_CORRECTED: p-value after False Discovery Rate corrected
Model: ICD10 +G_1_155162560 + T_1_155235843 + p22009_a1 + p22009_a2 + p22009_a3 + p22009_a4 + p22009_a5 + Year_of_birth + Townsend_deprivation_index + sex

Cox proportional hazards regression analysis of Parkinson's disease and endocrine, nutritional, metabolic, and digestive system disorders ICD-10 codes adjusted for polygenic risk Z-scores, GBA1 (G_1_155162560 + T_1_155235843) status, principal components 1-5, year of birth, Townsend deprivation index, and sex

UKB field corresponding to the ICD_10 code	ICD10_code	Definition of ICD10_code	Hazard Ratio	ci_min	ci_max	P_VAL	N_pairs	n	P_VAL_FDR_CORRECTED	rejected
p131650	K64	haemorrhoids and perianal venous thrombosis	0.59	0.50	0.71	3.34E-09	136	20006	6.55E-08	TRUE
p131654	K66	other disorders of peritoneum	0.62	0.45	0.87	5.63E-03	35	4612	4.60E-02	TRUE
p131648	K63	other diseases of intestine	0.69	0.59	0.80	1.12E-06	183	19139	1.83E-05	TRUE
p131636	K57	diverticular disease of intestine	0.69	0.62	0.77	8.19E-11	354	34801	2.68E-09	TRUE
p130792	E66	obesity	0.82	0.71	0.95	6.37E-03	211	22453	4.80E-02	TRUE
p130708	E11	non-insulin-dependent diabetes mellitus	1.21	1.08	1.36	1.10E-03	330	18815	1.17E-02	TRUE
p131600	K30	dyspepsia	1.33	1.12	1.58	1.19E-03	135	8811	1.17E-02	TRUE
p131640	K59	other functional intestinal disorders	1.55	1.38	1.75	8.57E-13	301	15431	8.40E-11	TRUE
p130714	E14	unspecified diabetes mellitus	1.61	1.39	1.86	1.91E-10	197	8658	4.68E-09	TRUE
p130770	E53	deficiency of other b group vitamins	1.73	1.30	2.30	1.71E-04	48	1907	2.10E-03	TRUE
p130718	E16	other disorders of pancreatic internal secretion	1.81	1.34	2.43	1.05E-04	44	1602	1.47E-03	TRUE
p130706	E10	insulin-dependent diabetes mellitus	2.66	2.03	3.50	1.78E-12	53	1608	8.70E-11	TRUE

PRS: Polygenic risk score
UKB: UK Biobank
PD: Parkinson's disease
ci_min: Confidence Interval minimum
ci_max: Confidence Interval maximum
P_VAL: p-value
N_pairs: Number of individuals identified with both ICD-10 code and neurodegenerative disease outcome
n: Number of Individulas Identified with ICD10_code
P_VAL_FDR_CORRECTED: p-value after False Discovery Rate corrected
Model: ICD10 + zSCORE + G_1_155162560 + T_1_155235843 + p22009_a1 + p22009_a2 + p22009_a3 + p22009_a4 + p22009_a5 + Year_of_birth + Townsend_deprivation_index + sex

Cox proportional hazards regression analysis of Parkinson's disease and endocrine, nutritional, metabolic, and digestive system disorders ICD-10 codes adjusted for LRRK2 (C_12_40220632 + G_12_40340400) status, principal components 1-5, year of birth, Townsend deprivation index, and sex

UKB field corresponding to the ICD_10 code	ICD10_code	Definition of ICD10_code	Hazard Ratio	ci_min	ci_max	P_VAL	N_pairs	n	P_VAL_FDR_CORRECTED	rejected
p131650	K64	haemorrhoids and perianal venous thrombosis	0.59	0.50	0.70	2.83E-09	136	20006	5.56E-08	TRUE
p131654	K66	other disorders of peritoneum	0.62	0.45	0.87	5.54E-03	35	4612	4.17E-02	TRUE
p131636	K57	diverticular disease of intestine	0.69	0.62	0.77	7.83E-11	354	34801	2.56E-09	TRUE
p131648	K63	other diseases of intestine	0.69	0.59	0.80	1.40E-06	183	19139	2.29E-05	TRUE
p130792	E66	obesity	0.82	0.71	0.94	4.46E-03	211	22453	3.97E-02	TRUE
p130708	E11	non-insulin-dependent diabetes mellitus	1.21	1.07	1.36	1.49E-03	330	18815	1.46E-02	TRUE
p131600	K30	dyspepsia	1.34	1.13	1.59	9.28E-04	135	8811	1.01E-02	TRUE
p131640	K59	other functional intestinal disorders	1.56	1.38	1.76	5.89E-13	301	15431	5.77E-11	TRUE
p130714	E14	unspecified diabetes mellitus	1.60	1.38	1.85	2.41E-10	197	8658	5.90E-09	TRUE
p130770	E53	deficiency of other b group vitamins	1.73	1.30	2.31	1.63E-04	48	1907	1.99E-03	TRUE
p130718	E16	other disorders of pancreatic internal secretion	1.83	1.36	2.46	7.69E-05	44	1602	1.08E-03	TRUE
p130706	E10	insulin-dependent diabetes mellitus	2.63	2.00	3.45	3.48E-12	53	1608	1.70E-10	TRUE

UKB: UK Biobank
PD: Parkinson's disease
ci_min: Confidence Interval minimum
ci_max: Confidence Interval maximum
P_VAL: p-value
N_pairs: Number of individuals identified with both ICD-10 code and neurodegenerative disease outcome
n: Number of Individulas Identified with ICD10_code
P_VAL_FDR_CORRECTED: p-value after False Discovery Rate corrected
Model: ICD10 + C_12_40220632 + G_12_40340400 + p22009_a1 + p22009_a2 + p22009_a3 + p22009_a4 + p22009_a5 + Year_of_birth + Townsend_deprivation_index + sex

Cox proportional hazards regression analysis of Parkinson's disease and endocrine, nutritional, metabolic, and digestive system disorders ICD-10 codes adjusted for polygenic risk Z-scores, LRRK2 (C_12_40220632 + G_12_40340400) status, principal components 1-5, year of birth, Townsend deprivation index, and sex										
**UKB field corresponding to the ICD_10 code**	**ICD10_code**	**Definition of ICD10_code**	**Hazard Ratio**	**ci_min**	**ci_max**	**P_VAL**	**N_pairs**	**n**	**P_VAL_FDR_CORRECTED**	**rejected**
p131650	K64	haemorrhoids and perianal venous thrombosis	0.59	0.50	0.70	3.02E-09	136	20006	5.91E-08	TRUE
p131654	K66	other disorders of peritoneum	0.62	0.45	0.87	5.56E-03	35	4612	4.53E-02	TRUE
p131648	K63	other diseases of intestine	0.69	0.59	0.80	1.08E-06	183	19139	1.77E-05	TRUE
p131636	K57	diverticular disease of intestine	0.69	0.62	0.77	8.22E-11	354	34801	2.69E-09	TRUE
p130792	E66	obesity	0.82	0.71	0.95	6.01E-03	211	22453	4.53E-02	TRUE
p130708	E11	non-insulin-dependent diabetes mellitus	1.21	1.08	1.36	1.07E-03	330	18815	1.13E-02	TRUE
p131600	K30	dyspepsia	1.33	1.12	1.58	1.16E-03	135	8811	1.13E-02	TRUE
p131640	K59	other functional intestinal disorders	1.55	1.37	1.75	9.20E-13	301	15431	8.97E-11	TRUE
p130714	E14	unspecified diabetes mellitus	1.61	1.39	1.86	1.80E-10	197	8658	4.40E-09	TRUE
p130770	E53	deficiency of other b group vitamins	1.73	1.30	2.31	1.66E-04	48	1907	2.03E-03	TRUE
p130718	E16	other disorders of pancreatic internal secretion	1.81	1.34	2.44	9.66E-05	44	1602	1.35E-03	TRUE
p130706	E10	insulin-dependent diabetes mellitus	2.66	2.03	3.50	1.83E-12	53	1608	8.97E-11	TRUE

PRS: Polygenic risk score
UKB: UK Biobank
PD: Parkinson's disease
ci_min: Confidence Interval minimum
ci_max: Confidence Interval maximum
P_VAL: p-value
N_pairs: Number of individuals identified with both ICD-10 code and neurodegenerative disease outcome
n: Number of Individulas Identified with ICD10_code
P_VAL_FDR_CORRECTED: p-value after False Discovery Rate corrected
Model: ICD10 + zSCORE + C_12_40220632 + G_12_40340400 + p22009_a1 + p22009_a2 + p22009_a3 + p22009_a4 + p22009_a5 + Year_of_birth + Townsend_deprivation_index + sex
